# Investigating Penetration and Antimicrobial Activity of Vector-Bicycle Conjugates

**DOI:** 10.1021/acsinfecdis.3c00427

**Published:** 2024-06-12

**Authors:** Andreas Hadjicharalambous, Hector Newman, Nick Lewis, Catherine Rowland, Nikolaos Bournakas, Steven J. Stanway, Michael Dawson, Michael J. Skynner, Paul Beswick

**Affiliations:** † Department of Biochemistry, University of Cambridge, Cambridge CB2 1QN, U.K.; ‡ BicycleTx Limited, Portway Building, Granta Park, Cambridge CB21 6GS, U.K.; § School of Life Sciences, University of Warwick, Coventry CV4 7AL, U.K.

**Keywords:** bicycle, antimicrobial resistance, membrane active peptides, antimicrobial peptides, outer membrane, antibiotics

## Abstract

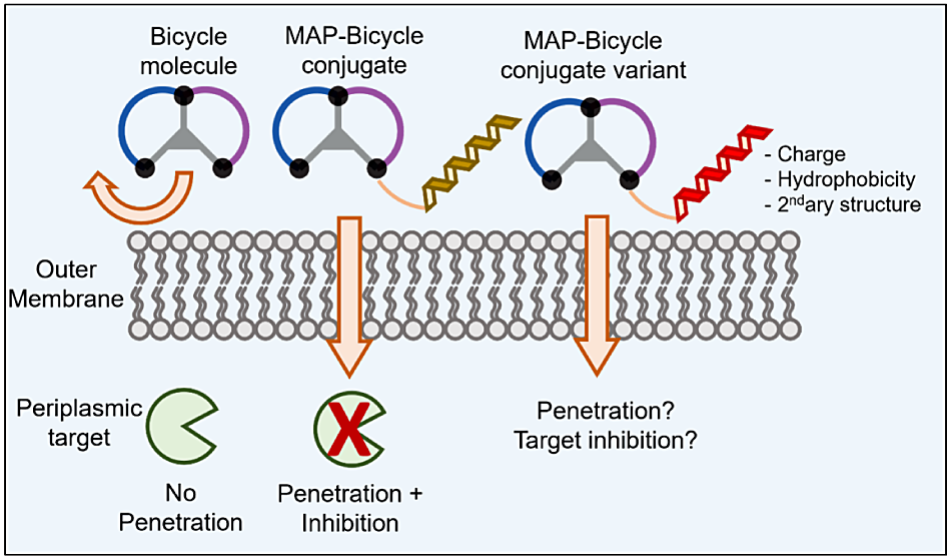

Growing antibiotic resistance is rapidly threatening the efficacy of treatments for Gram-negative infections. Bicycle molecules, constrained bicyclic peptides from diverse libraries generated by bacteriophage display that bind with high affinity to a chosen target are a potential new class of antibiotics. The generally impermeable bacterial outer membrane currently limits the access of peptides to bacteria. The conjugation of membrane active peptides offers an avenue for outer membrane penetration. Here, we investigate which physicochemical properties of a specific membrane active peptide (MAP), derived from ixosin-B, could be tweaked to enhance the penetration of conjugates by generating multiple MAP-Bicycle conjugate variants. We demonstrate that charge and hydrophobicity are important factors, which enhance penetration and, therefore, antimicrobial potency. Interestingly, we show that induction of secondary structure, but not a change in amphipathicity, is vital for effective penetration of the Gram-negative outer membrane. These results offer insights into the ways vectors could be designed to deliver Bicycle molecules (and other cargos) through biological membranes.

Antimicrobial resistance continues to be a rapidly advancing global healthcare threat.^1^ The increasing difficulty in treating Gram-negative multidrug resistant pathogens using current antibiotics means there is a pressing need to develop new classes of antibiotics.^2,3^ Constrained peptides have shown therapeutic potential as a novel modality in many disease areas, including as antimicrobials where they structurally resemble many naturally occurring ligands.^4,5^ Naturally occurring bicyclic compounds with various biological activities, such as inhibition of eukaryotic cell division and antitumor activity, have been previously isolated.^6,7^ Bicycle^Ⓡ^ molecules are a specific class of constrained peptides where a central small molecule scaffold is covalently reacted to cysteines within a linear peptide sequence. This provides structural constraint and creates a bicyclic structure.^4,8,9^ Enormously diverse libraries of these Bicycle molecules can rapidly be screened, using modified bacteriophage display, to identify high-affinity binders against a variety of prokaryotic targets. These can be rapidly optimized using simple peptide and medicinal chemistry approaches to build in desired drug-like properties, which makes Bicycle molecules a potentially promising new group of constrained peptides for antibiotic discovery.^8^ Bicycle molecules also structurally resemble many classes of antibiotics, many of which are derived from natural products. However, in contrast to naturally occurring antibiotics, Bicycle molecules are readily chemically modified and can be easily “tuned” to a particular pharmacology or drug-like property.

To effectively treat Gram-negative pathogens, a novel antibiotic must first cross the bacterial outer membrane (OM) to reach its therapeutic target in the periplasmic or cytoplasmic space. The generally impermeable OM of Gram-negative bacteria is the major factor, which prevents entry of most molecules larger than 700 Da,^10^ including Bicycle molecules. The OM is an asymmetric lipid bilayer, with the inner leaflet composed of phospholipids, while the outer leaflet is made of lipopolysaccharides (LPS). The major components of LPS are Lipid A, a polysaccharide core, and the cationic O-antigen. Lipid A molecules interface with the inner leaflet by using their hydrophobic tails. The polysaccharide core extends outward from Lipid A, connecting to repeating oligosaccharide units, the O-antigen. Divalent cations bind between LPS molecules, partly neutralizing the negatively charged phosphate groups of the LPS. The OM also contains porins: barrel-like protein channels of limited diameter, which allow the diffusion of small molecules.^10^ The well-hydrated carbohydrate chains of the outer leaflet, combined with the hydrophobic nature of a bilayer, grant high impermeability to the OM.^11^

Some groups of larger molecules can translocate across the bacterial OM, one of which is the group of membrane active peptides (MAPs).^12^ MAPs are short synthetic or natural oligopeptides that can penetrate biological membranes, including the bacterial OM. MAPs include antimicrobial peptides^13^ and cell penetrating peptides.^14^ The mechanisms employed by MAPs in crossing membranes are varied and dependent on the specific MAP and the target membrane.^12^ However, their OM penetrating ability has been exploited through conjugation as “vectors” to various “cargoes” for delivery into Gram-negative bacteria.^12,15−17^ For example, a synthetic MAP has been used to deliver antisense peptide-nucleic acids for gene silencing into the cytoplasm of Gram-negative bacteria^15^ while various MAP-vancomycin conjugates have been used to repurpose vancomycin (a classically Gram-positive specific antibiotic that cannot cross the Gram-negative OM) into a Gram-negative antibiotic.^16^ MAP-Bicycle conjugates could therefore also be used to overcome the OM barrier.

In previous work, we showed that by covalently conjugating a MAP to a Bicycle molecule directed at a periplasmic target involved in peptidoglycan biogenesis, we enhanced the antimicrobial activity of the Bicycle molecule in pathogenic strains of a range of Gram-negative species, specifically: *E. coli*, *A. baumannii* and *P. aeruginosa*.^5^ The peptide used as the “vector” was a synthetic derivative of a natural MAP from the salivary glands of the tick *I. sinensis*.^18^ This MAP, referred to here as AV1, has also previously been shown to exhibit limited penetration into human red blood cells, making it a promising “vector” selective for the bacterial OM.^5,18^ However, it is not clear which properties of AV1 drive the OM penetration. It is well established that MAPs often contain a high number of positively charged and hydrophobic residues, while others assume a secondary structure when in contact with a membrane, such as the OM.^12,19^ In this study, we set out to explore which physicochemical factors of AV1 drive OM penetration when conjugated to an *E. coli* PBP3-targeting Bicyclic peptide.^9^ By varying amino acids within the “vector” component of a MAP-Bicycle conjugate, we show that variants with higher positive charge and hydrophobicity enhanced antibacterial activity. Interestingly, altering the amphipathicity did not affect the antimicrobial activity of the conjugate but impaired the ability of the “vector” to adopt a secondary structure.

## Results

**Figure 1:**
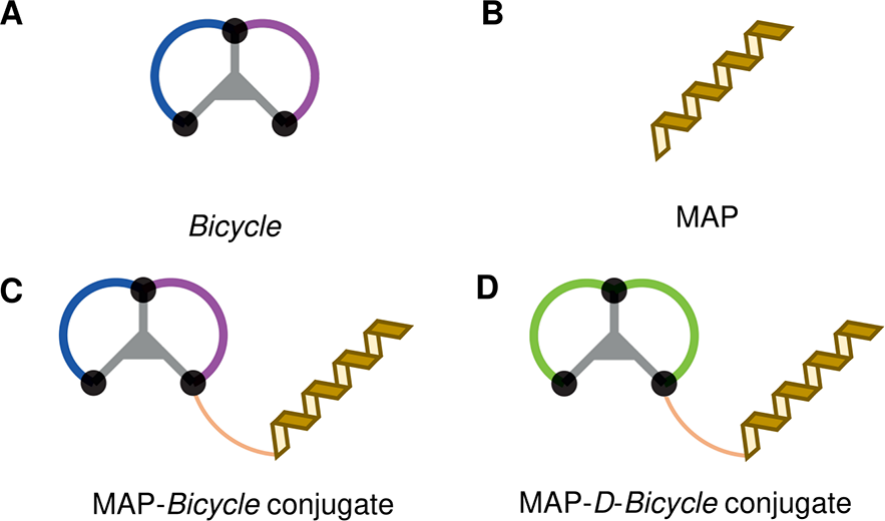
Schematic representation of the molecules used in this study. (A) Bicycle molecules are bicyclic peptides (blue–purple) constrained by a central scaffold (gray). (B) Membrane active peptides (MAPs) are peptides that can penetrate biological membranes. MAPs in this study were made from d-amino acids. (C) MAP-Bicycle conjugates (blue) are molecules in which the MAP is covalently attached to the Bicycle molecule. (D) MAP-d*-*Bicycle conjugates (green) have the same Bicycle molecule sequence but with d-amino acids.

To synthesize MAP-Bicycle conjugates, different variants of a previously tested MAP (DRAMP18563 in Wagstaff *et al.*, 2020)^5^ were generated with a C-terminal 3-azido-l-alanine (CAzal) group. A previously identified Bicycle molecule, which inhibits its periplasmic target (described previously as peptide **2**) was also generated with a C-terminal Lys(pentynoyl)-CONH (**AB1**).^9^ Copper(I)-catalyzed azide–alkyne cycloaddition (CuAAC) was used to “click” the vector variant, with the Bicycle molecule generating the MAP-Bicycle conjugates (Table 1). The MAPs, whether conjugated to the original Bicycle molecule or not, were in the retro-inverso form (i.e., sequence inversion and d*-*amino acids). Retro-inverso MAPs have been shown to be more efficient at penetration, possibly due to proteolytic resistance.^20,21^Figure 1 shows the types of molecules used in this study.

**Table 1:**
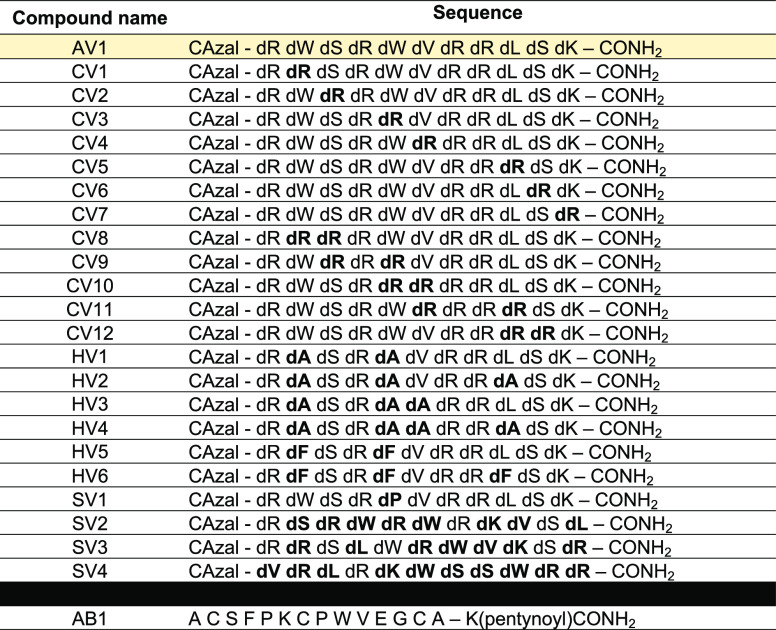
Sequence of the MAP Vector Variants^a^

a The original MAP vector is shown in light-yellow. “d″ denotes d-amino acids. Bicycle conjugates were generated as discussed in the text and methods using the CAzal moiety, while the other C-terminus of the peptide was amidated (CONH_2_). For each variant, the amino acids changed compared to the original sequence (AV1) are shown in bold. All vectors were conjugated to AB1 via click chemistry.

### Antimicrobial Activity

To determine how the cell killing ability of the vector-Bicycle variants changed when compared to the original vector, their antimicrobial activity was examined against a clinical strain of *E. coli* (ATCC25922) in cation-adjusted Mueller Hinton broth (CaMHB). The minimum inhibitory concentration (MIC) is defined as the lowest concentration of compound that completely prevents bacterial growth (Table 2). The unconjugated Bicycle molecule (AB1) and the original unconjugated vector (AV1) were used to make the MAP-Bicycle conjugate A1. All MAP-Bicycle conjugates contain the AB1 Bicycle molecule, linked to a variant of the original MAP (e.g., C2, S2, H6). The unconjugated MAP variants are labeled with the equivalent letter and number but with a “V” included in the name (e.g., CV2, SV2, HV6, see Table 1 for all vector variants).

**Table 2:** MIC Values for the Tested Molecules^a^

Bicycle molecule								
compound name	**MIC(μg/mL)**	**MIC(μM)**						
AB1	>64	>32.7						
**MAP-Bicycleconjugate**			MAP			**MAP-**d**-bicycle**		
compound name	**MIC(μg/mL)**	**MIC(μM)**	compound name	**MIC(μg/mL)**	**MIC(μM)**	compound name	**MIC(μg/mL)**	**MIC(μM)**
A1	8–16	2.2–4.5	AV1	32	19.5	-	**-**	**-**
C1	4–16	1.1–4.5	CV1	>64	>39.7	-	**-**	**-**
C2	4–16	1.1–4.4	CV2	16	9.4	CD2	>64	>17.5
C3	4–8	1.1–2.2	CV3	>64	>39.7	-	-	**-**
C4	4–8	1.1–2.2	CV4	32	18.8	CD4	>64	>17.5
C5	4–8	1.1–2.2	CV5	32	19	CD5	>64	>17.6
C6	4–32	1.1–8.7	CV6	16	18.7	-	**-**	**-**
C7	8–32	2.2–8.8	-	-	-	-	**-**	**-**
C8	4	1.1	CV8	64–>64	38.1–>38.1	-	**-**	**-**
C9	4–8	1.1–2.2	CV9	64 −>64	38.1–>38.1	-	**-**	**-**
C10	4	1.1	CV10	>64	38.3–>38.3	-	**-**	**-**
C11	2–4	0.5–1.1	CV11	32–64	18.4–36.8	CD11	>64	>17.3
C12	2–32	0.5–8.6	CV12	16	36.5	-	**-**	**-**
H1	64–>64	19.0–>19.0	HV1	>64	>45.4	-	**-**	**-**
H2	>64	>19.2	HV2	>64	>46.8	-	**-**	**-**
H3	32–>64	4.8–>9.6	HV3	>64	>46.3	-	**-**	**-**
H4	64–>64	19.4–>19.4	HV4	>64	>47.7	-	**-**	**-**
H5	16–32	4.5–9.1	HV5	>64	>41.0	-	**-**	**-**
H6	8	2.3	HV6	>64	>40.1	-	**-**	**-**
S1	32	9.1	SV1	>64	>41.2	-	**-**	**-**
S2	8	2.2	SV2	32	19.5	SD2	>64	>17.8
S3	4–8	1.1–2.2	SV3	16	9.8	SD3	>64	>17.8
S4	8	2.2	SV4	32–64	19.5–39.0	SD4	>64	>17.8

a AB1 at the top of the table refers to the unconjugated Bicycle molecule, which is used in all MAP-Bicycle conjugates. The original MAP-Bicycle conjugate (A1) and variants are shown on the left. The corresponding standalone MAPs are in the middle and have a “V” in their designation (e.g., AV1, CV1). For some MAP variants, corresponding MAP-d-Bicycle conjugates are on the right of the table and have a “D” designation (e.g., CD2, CD4). MIC values are shown in μg/mL and μM.

The unconjugated Bicycle molecule (AB1) shows an MIC of more than 32.7 μM while the vector-Bicycle conjugate containing the original MAP peptide (A1)^18^ shows a 10-fold lower MIC, when adjusting for the change in molecular weight, suggesting that conjugation of a MAP vector allows penetration and therefore more efficient access to the target, leading to cell death (compare μM MICs, Table 2). Furthermore, in all cases, the standalone MAP variant had very low or lower antimicrobial activity than the conjugate (Table 2). For certain unconjugated MAPs that showed vector-mediated activity, a conjugation to an all-d-amino acid variant of the Bicycle molecule was made (e.g., CD2, SD2). We could not detect antimicrobial activity in these all-d conjugated molecules (Table 2). Therefore, although in some cases, there is some antimicrobial activity by the MAP vector, penetration is a consequence of the vector while the antimicrobial efficacy lies in the ability of the Bicycle molecule to inhibit its periplasmic target.

To examine how changing the location and number of charged residues in the MAP vector affects antimicrobial activity of the conjugate, an “arginine scan” was conducted using either one or two arginine residues for positions, which did not already contain an arginine residue (Table 1, CV1–CV12). Compared to the original MAP-Bicycle conjugate (A1), variants with a single arginine addition (C1–C7) showed either had narrower MIC range (C3, C4, and C5), or an MIC range with a smaller lower bound (C1, C2, C6), which could suggest more effective killing. The exception was C7, which showed a broader MIC range. This could be due to the terminal lysine being important for effective penetration. Conjugates with a double arginine substitution (C8–C12) had generally lower MIC values (C8 and C10) or narrower MIC ranges (C9 and C11) than the original MAP-Bicycle conjugate (A1). This could indicate that they are more effective at cell killing than the original MAP-Bicycle conjugate (A1). The only exception is C12 showing a higher upper bound than A1, which could be due to its inconsistency in one of its MIC assay repeats (see Table S1). Overall, as expected, we observed that increasing the positive charge of the MAP vector enhances the antimicrobial activity of the vector-Bicycle conjugate.

Hydrophobicity has also been shown to enhance penetration of MAPs.^22^ The original ixosin B-derived MAP (AV1) contains hydrophobic leucine, valine, and two tryptophan residues.^18^ Various alanine substitutions were made to decrease the hydrophobicity of the MAP vector to determine how the tryptophan residues, in combination with leucine and valine residues, contributed to the penetration efficiency of the conjugate (H1–H4). In addition, the tryptophan residues were substituted with phenylalanine (which has a hydrophobicity between tryptophan and leucine) in H5. In H6, both tryptophan and leucine residues were replaced by phenylalanine. The MIC values of these variants are higher than the original compound (Table 2). This indicates that the antimicrobial activity of all of the alanine variants was abolished. The substitution of tryptophan for phenylalanine residues (H5) increased the MIC compared with the original conjugate but lowered the MIC compared with the alanine variants (H1–H4). The additional substitution of leucine with phenylalanine (H6) restored the MIC value to the level seen with the original MAP-Bicycle conjugate (A1). These results suggest that the overall hydrophobicity of the vector is an important component to consider when designing MAP-Bicycle conjugates, as it enhances antimicrobial activity.

The ability of a MAP to transition from a random coil conformation in solution to a well-defined secondary structure is thought to be important for its interaction with and ability to penetrate the OM.^12,23,24^ To test if the secondary structure of the vector affected the cell killing ability of the conjugate, a proline was introduced in the sequence (S1). Proline is a known secondary structure disruptor^25^ as it does not have hydrogen on its peptide bond nitrogen to participate in intrahelix hydrogen bonding. S1 showed diminished antimicrobial activity compared to the original conjugate (A1), suggesting that when the ability of the MAP to form a secondary structure is abolished, the penetration is also lowered. However, this could be due to the substitution of a tryptophan residue with proline, lowering the hydrophobicity of the MAP variant. To investigate whether the specific amino acid sequence is important for penetration (because it facilitates the formation of secondary structures by the interaction of neighboring amino acids), three “scrambled” variants, with the same amino acids but randomly placed along the primary sequence, were created (S2–S4). Interestingly, the “scrambled” variants had similar, if not slightly improved, MIC values compared to the original sequence. From these results, it was unclear whether the secondary structure was important in allowing penetration of the MAP-Bicycle conjugate.

### Secondary Structure Studies

**Figure 2:**
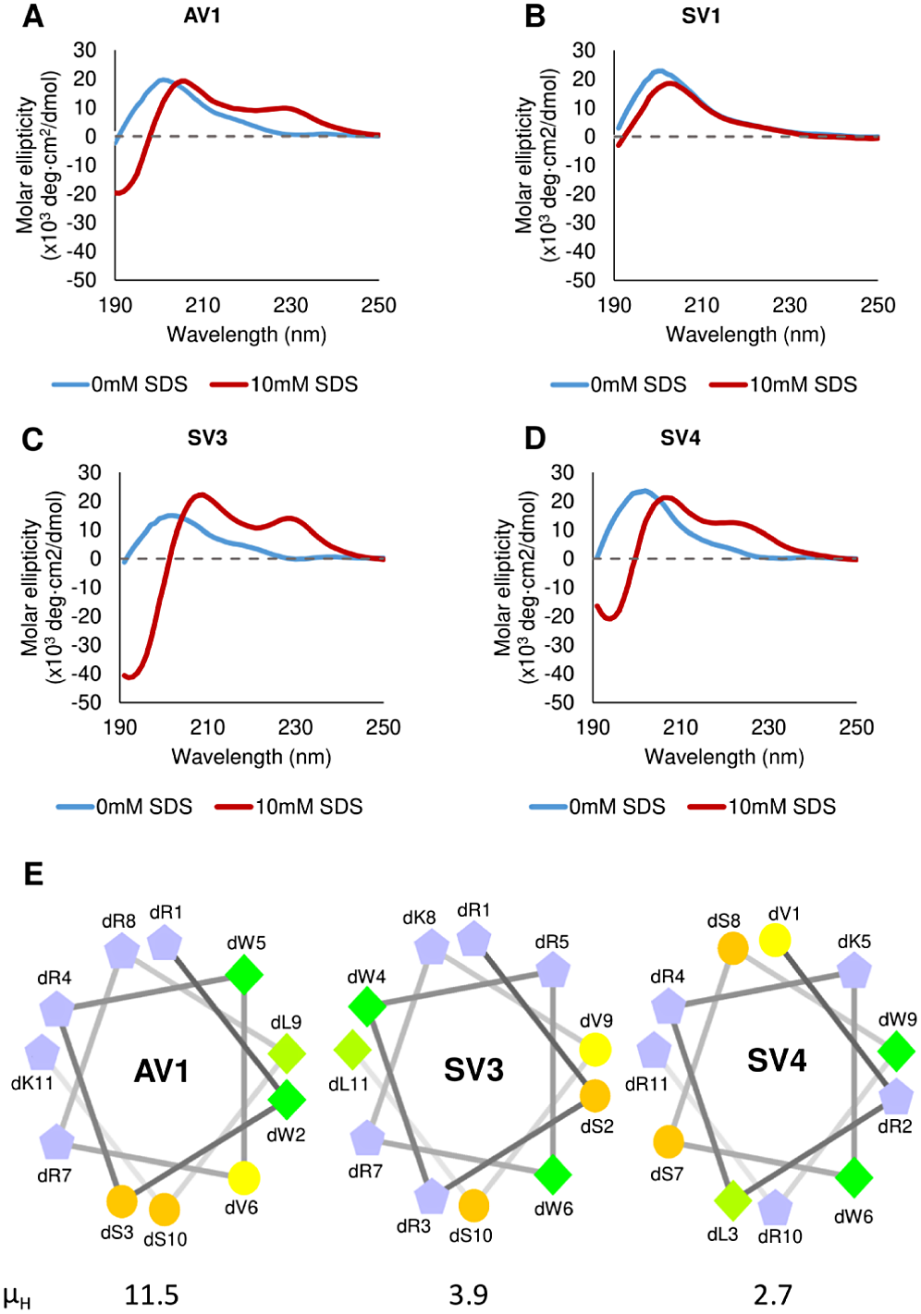
CD spectra and helical projection wheels of MAPs investigated. (A–D) CD spectra for the MAP vectors AV1 (A), SV1 (B), SV3 (C), and SV4 (D) measured at a concentration of 0.15 mg/mL in a 10 mM sodium phosphate buffer, pH 7.0. The spectra were measured in either 0 mM (blue line) or 10 mM SDS (red line). (E) Helical projection wheels for MAP vectors AV1, SV3, and SV4. The d*-*amino acid residue and its number are shown close to its corresponding position. Positively charged residues are shown in blue pentagons, charged residues are shown in orange circles, and hydrophobic residues are shown in deep or light green squares or yellow circles, with higher hydrophobicity residues being closer to green and lower hydrophobicity residues being closer to yellow. μ_Η_ corresponds to the hydrophobic moment of each peptide, a measure of the amphipathicity of the helix (the greater the value, the more amphipathic the helix).

We hypothesized that “scrambled” variants might not have a disrupted secondary structure, explaining why they have similar antimicrobial activities to the original sequence. To explore this, the circular dichroism (CD) spectra of unconjugated “vectors” were analyzed. To test if the secondary structure of vectors changes in the presence of a membrane, spectra were collected in buffers with or without sodium dodecyl sulfate (SDS), a surfactant that forms micelles, mimicking biological membranes (Figure 2). Note that the spectra generated from these MAPs are reflections on the horizontal axis of “typical” (l*-*amino acid) secondary structures because they are made of d*-*amino acids.

As expected, the original vector (AV1) adopted a different secondary structure in the presence of SDS compared to buffer alone, with positive bands present at around the 208 and 230 nm wavelength, suggesting the formation of an α helix (Figure 2A, Table 3). Vectors SV3 and SV4 showed a similar change in secondary structure (Figure 2C,D, Table 3) while vector SV1, containing a proline in the middle of the vector sequence, had a minimal change in its secondary structure (Figure 2B). Interestingly, although the secondary structure of vector SV2 did change, it did not resemble the CD spectrum of an α helix but of a polyproline II (PP-II) helix (Figure S3). Proline-rich antimicrobial peptides have been shown to assume this structure.^27,28^ Since vectors AV1, SV3, and SV4 seem to assume an α-helical structure, their helical projection wheels (Figure 2E), representing their amphipathicity (the degree of separation of hydrophobic and hydrophilic residues on opposing sides of a helix), were generated to determine whether these properties were conserved during “scrambling” of their amino acids. The hydrophobic moment (μ_H_) of each MAP variant was also calculated. As shown in Figure 2E, the amphipathicity is not conserved between the original MAP vector and the two “scrambled” variants. Together, these data suggest that, for these MAPs, the ability to form secondary structures in the presence of the OM is an important factor for efficient penetration. At the same time, a decrease in amphipathicity does not seem to affect the ability of these MAPs to penetrate the OM.

**Table 3:** Predicted Percentage Helicity of MAP Variants SV1–SV4 Generated Using the Online Tool K2D3^26^

	% helicity	
MAP	0 mM SDS	10 mM SDS
AV1	18.04	82.53
SV1	10.29	19.57
SV2	9.33	12.54
SV3	11.21	95.27
SV4	10.31	86.65

### Outer Membrane Penetration

We then sought to understand how conjugation of Bicycle molecules to MAPs and adjustments of the physicochemical properties of the vector in a MAP-Bicycle conjugate affect penetration. Permeation of the OM was followed using *N*-phenyl-1-naphthylamine (NPN) fluorescence in the presence of various MAP variants and their equivalent MAP-Bicycle conjugates, representing variations in charge magnitude, hydrophobicity, and secondary structure. NPN is a chemical that fluoresces weakly in an aqueous environment but strongly in a hydrophobic environment. NPN is normally excluded from an undamaged bacterial OM but, upon permeabilization, NPN associates with phospholipids, resulting in fluorescence.

**Figure 3:**
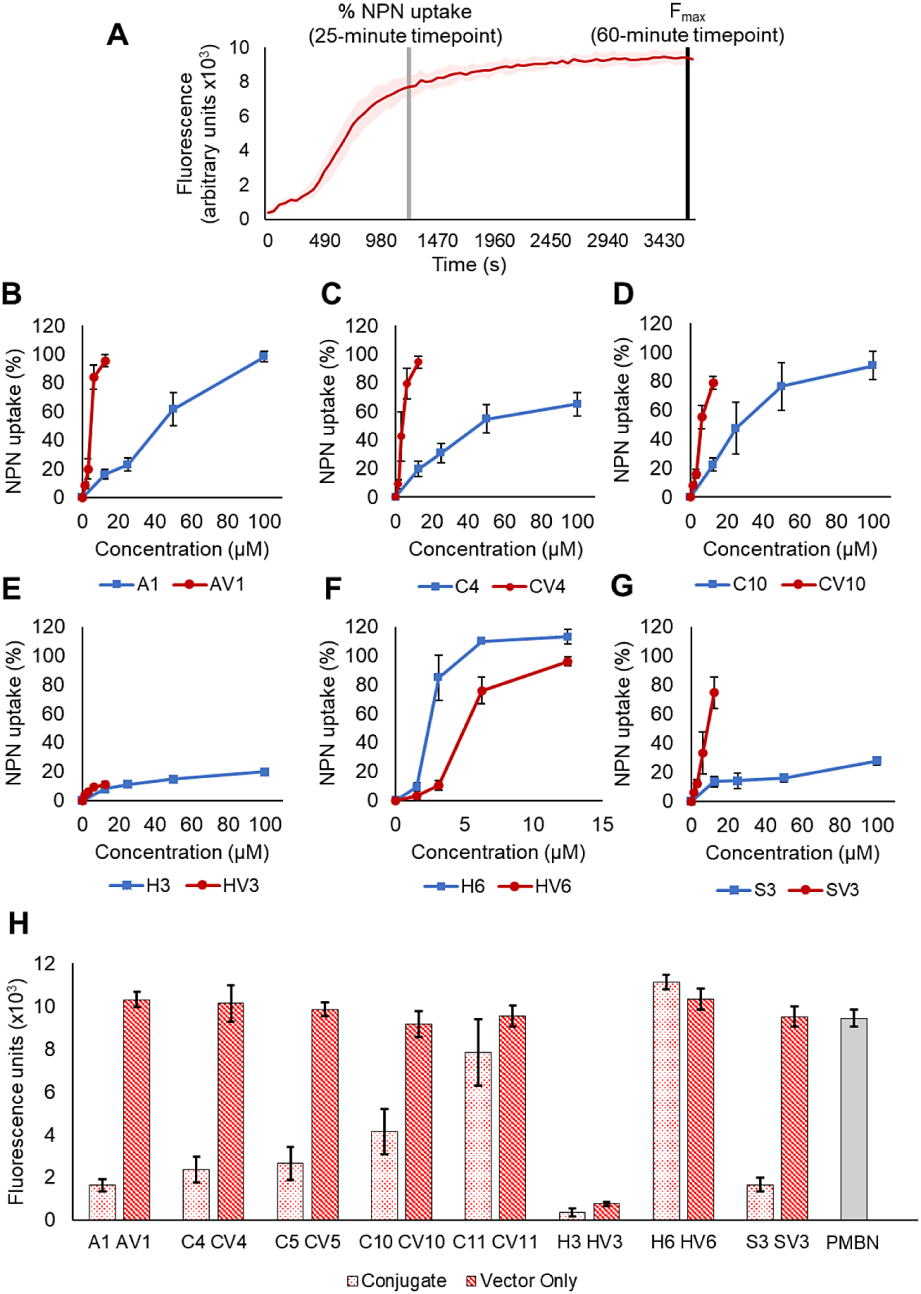
NPN uptake of different MAP vectors and MAP-Bicycle conjugates. (A) Representation of fluorescence timecourse experiment data generated using the NPN assay. An increase in fluorescence represents NPN incorporation in a lipid environment and, therefore, OM penetration. Percentage (%) NPN uptake (B–G) was calculated using the fluorescence at 25 min while comparison for the maximum fluorescence (*F*_max_, H) was done using fluorescence after 1 h. %NPN uptake for MAP vectors or their equivalent MAP-Bicycle conjugate; (B) A1/AV1, (C) C4/CV4, (D) C10/CV10, (E) H3/HV3, (F) H6/HV6, (G) S3/SV3. The standalone MAP vector is in red, while the MAP-Bicycle conjugate is in blue. Error bars represent the standard deviation (*n* = 3). (H) Maximum fluorescence (*F*_max_) for the MAP-Bicycle conjugate (pink) and its corresponding standalone MAP vector (red). In gray, the *F*_max_ of PMBN, a known OM penetrator,^29^ is shown for comparison. Error bars represent standard deviation (*n* = 3).

To investigate how changes in the MAP vector affect penetration, each MAP and MAP-Bicycle was incubated with bacteria, and the change in fluorescence was monitored over time (Figure 3A; Figure S1 shows all the timecourse graphs analyzed). From these fluorescence time course graphs, %NPN uptake (Figures 3B–G, S2) and the maximum fluorescence reached (*F*_max_, Figure 3H) were compared between free MAP and the corresponding MAP-Bicycle conjugate.

Replacing arginine with alanine residues abolished the ability of the MAP-Bicycle conjugate to penetrate the OM (compare blue lines in Figure 3B,E), while increasing the charge of the MAP by two additional arginine residues (compare blue lines in Figure 3B,D) enhanced penetration of the MAP-Bicycle conjugate. This agrees with the antimicrobial activity observed in Table 1. Interestingly, a “scrambled” MAP-Bicycle variant showed diminished penetration efficiency compared to the original MAP-Bicycle conjugate (Figure 3B,G, blue lines) despite exhibiting a similar antimicrobial activity (Table 1). This suggests that although the “scrambled” variant is slower in penetration (Figure S1A,G), it is an equally potent antimicrobial.

In most cases, the MAP-Bicycle conjugate had a lower efficiency of penetration than the free MAP (Figure 3B–E,G). This suggests that the conjugation of a Bicycle molecule near the MAP affects its penetration efficiency and kinetics (Figure S1). An explanation could be that attachment of a bulky moiety, such as a Bicycle, prevents close proximity between different MAP molecules which, in many cases, is necessary for the generation of a pore, which allows their translocation through a membrane barrier.^12,13^ Unexpectedly, H6, a conjugate variant where three tryptophan residues are replacing the two tryptophan and one leucine residue, causes a higher %NPN uptake at any given concentration compared to its free MAP. In addition, it seems that the penetration kinetics of H6 compared to HV6 are faster (Figure S1G), indicating that the MAP-Bicycle conjugate penetrates faster and more efficiently than its unconjugated vector as the *F*_max_ reached is higher.

## Discussion

**Figure 4:**
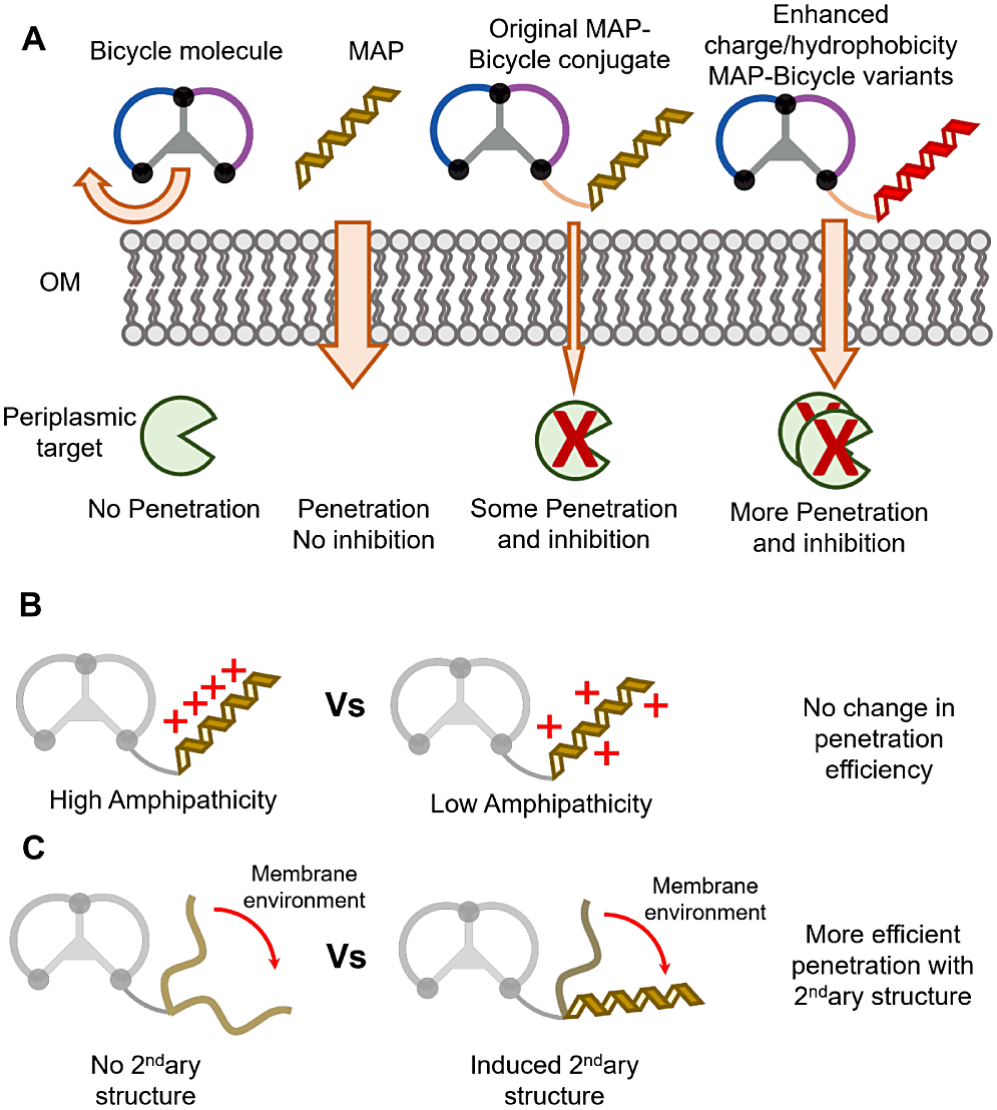
Aspects which affect penetration of the OM and inhibition of a periplasmic target for enhanced antimicrobial activity. (A) Bicycle molecules without a MAP vector are unable to penetrate the OM. MAP vectors can penetrate the OM but do not target the periplasmic target. MAP-Bicycle conjugates penetrate (albeit less efficiently) and inhibit the periplasmic target. Increased hydrophobicity and charge enhance penetration of the OM. (B) MAP amphipathicity did not affect penetration. (C) Secondary structure induction by MAP is important for penetration.

There is an urgent need for a new class of antibiotics due to the increasing threat of antimicrobial resistance worldwide. Bicycle molecules offer a potentially attractive solution for treating diseases arising from Gram-negative antibiotic-resistant pathogens if the challenge of OM penetration can be addressed. In this study, we conjugated a previously identified MAP^18^ to a Bicycle molecule selected against a periplasmic target and generated variations based on amino acid changes of the vector (Table 1).^9^ The MAP-Bicycle conjugate shows enhanced antimicrobial activity compared to either the standalone vector or unconjugated Bicycle molecule, suggesting that MAP conjugation enhances OM penetration and delivery of the Bicycle molecule to its periplasmic target (Table 2, Figure 4A).

In addition, as shown in this study and elsewhere,^22,30^ increasing the positive charge or hydrophobicity of the MAP vector enhances the antimicrobial activity of the conjugate Surprisingly, a random rearrangement of amino acids from the original MAP sequence does not seem to decrease the potency of the conjugate (Table 2, compare A1 with S2–4). This observation suggests that the primary amino acid sequence of this specific MAP is not crucial for penetration but rather the physicochemical characteristics granted by its constituent amino acids.^12,14^

The rearrangement of the amino acid sequence can lead to two changes in a peptide: its secondary structure and its amphipathicity. Moving from an aqueous buffer to a membrane mimicking environment, CD spectra of MAPs SV2–4 show secondary structure transitions, like the original AV1 sequence but unlike the proline-disrupted SV1 variant (Figure 2). This observation agrees with studies showing that the greater the extend of structural change from a disordered to an ordered conformation, the more likely the MAP will be a good penetrator.^24,31^ Secondary structure transition for these MAP-Bicycle conjugates is necessary for their translocation across the outer membrane.

The CD spectra of SV3 and SV4 suggest some helicity in their secondary structure. Although they are probably not perfect helices, comparing projection wheels of their sequences to that of the original AV1 MAP, there is a lower degree of separation between positively charged and hydrophobic side chains (Figure 2). Therefore, it seems that a decrease in amphipathicity does not diminish the ability of this MAP to deliver the Bicycle to its periplasmic target, since the MIC values between SV3, SV4, and AV1 are similar. This is, however, antithetic to some studies, which have shown that amphipathicity is an important aspect of penetration.^14,32,33^ Amphipathicity could be an important factor for membrane translocation for some MAPs but not others.

The penetration kinetics of a standalone MAP are affected by the conjugation of a Bicycle molecule. In most cases, conjugates exhibit slower penetration kinetics and lower penetration efficiency at 25 min compared to their MAP vectors (Figures 3 and 4). Membrane penetration of some MAPs has been shown to be less effective when conjugated with various cargoes.^34−36^ Interestingly, H6 showed faster penetration kinetics and higher penetration efficiency compared to its standalone MAP vector HV6 (Figure 3). Furthermore, the antimicrobial activity of H6 was marginally superior to the original MAP-Bicycle conjugate (Figure S4). It could be that the interactions between the MAP vector and the Bicycle molecule in the H6 conjugate somehow enhance the ability of the MAP to penetrate the OM effectively. The mechanism by which this is achieved is, however, unknown. Nevertheless, this study builds upon the unexplored area of how the conjugation of a cargo affects the penetration efficiency of MAPs. This is achieved by showcasing how the penetration kinetics of this MAP, and its variants, change with the addition of a Bicycle molecule.

## Conclusion

In summary, this study investigates how different physicochemical abilities of a MAP vector can affect the OM penetration and antimicrobial activity of Bicycles (Figure 4). A variety of MAP*-*Bicycle conjugates generated with differing vector charge, hydrophobicity, and secondary structure were investigated. The results indicate that MAP-Bicycle conjugates can penetrate the OM, and optimization of the vector moiety is an attractive strategy for the development of a novel antibiotic class.

## Materials and Methods

### Peptide Synthesis

All peptides were synthesized on Rink amide resin using standard Fmoc (9-fluorenylmethyloxycarbonyl) solid phase peptide synthesis using 2 automated systems. Peptide synthesis at 25 μmol was run on a Biotage SyroII automated synthesizer. Peptide synthesis (80–240 μmol) was carried out with a Gyros Symphony X automated synthesizer. Vector sequences were synthesized with an N-terminal 3-azidopropanoic acid (Iris Biotech), and Bicycle molecules were synthesized with a C-terminal lysine (pentynoyl) for use in copper(I)-catalyzed azide alkyne cycloaddition (CuAAC). Following cleavage from the resin using a cocktail of 95% TFA, 2.5% triisopropylsilane, and 2.5% H_2_O with 25 mg of dithiothreitol (DTT) per mL, peptides were precipitated with diethyl ether and dissolved in 50:50 acetonitrile/water. Linear vector peptides were lyophilized after cleavage. Peptides for bicyclization were diluted to 2 mM in 50:50 acetonitrile:water, 2.6 mM scaffold solution, and 200 mM ammonium bicarbonate to give final concentrations of 1, 1.3, and 100 mM respectively. Completion of cyclization was determined by matrix assisted laser desorption ionization time-of-flight (MALDI-TOF) or LC–MS. Once complete, the cyclization reaction was quenched using *N*-acetyl cysteine (10 equiv of 1 M solution over peptide) and lyophilized.

Standard Fmoc amino acids, as well as nonproteinogenic Fmoc amino acids, were obtained from Sigma-Aldrich Iris Biotech GmbH, Apollo Scientific, ,ChemImpex and Fluorochem.

Linear vector peptides were synthesized and purified by SB-Peptide (France).

### CuAAC Conjugation

All crude peptides were purified by RP-HPLC prior to conjugation. The alkyne-bearing bicyclic peptide was dissolved in anhydrous DMSO (Sigma-Aldrich) at 18 mM and added at equal volume to a solution of the corresponding azide-bearing vector compound in anhydrous DMSO at 15 mM to give 1.2 equiv of alkyne species over azide. Nitrogen was bubbled through HPLC grade water (Fisher Scientific) to degas and used to make 200 mM solutions of copper(II) sulfate pentahydrate (Alfa Aesar) and l-ascorbic acid (Sigma-Aldrich), which were added as 5 and 10 equiv, respectively, over alkyne. The reaction progression was measured by LC–MS analysis at 15 min intervals by quenching a small aliquot with 0.5 M EDTA (Lonza) as 2 equiv over Cu (II) and diluting to a final concentration of 100 μM with LC–MS grade acetonitrile:water (10:90) (Fisher Scientific). Upon completion of click conjugation, the bulk reaction solution was fully quenched before loading onto HPLC.

### Peptide Purification

Crude peptides, following lyophilization, were dissolved in an appropriate solvent system and filtered through a 0.45 μm PES filter before loading onto a 5 μm, 100 Å, 21.2 × 100 mm Kinetex XB-C18 column (Phenomenex). Prep HPLC gradients using 0.1% TFA in H_2_O (solvent A) and 0.1% TFA in acetonitrile (solvent B) were selected based on the retention time of samples analyzed either after cleavage or during cyclization on a 2.6 μm, 100 Å, 2.1 × 50 mm Kinetex XB-C18 analytical column on a gradient of 5–95% 0.1% TFA in acetonitrile.

Bicycle-vector peptide conjugates were loaded from quenched and diluted solutions onto a 5 μm, 100 Å, 21.2 × 100 mm Kinetex Biphenyl column (Phenomenex). Prep HPLC gradients using 0.1% TFA in H_2_O (solvent A) and 0.1% TFA in acetonitrile (solvent B) were selected based on retention time of samples analyzed during click conjugation on a 2.6 μm, 100 Å, 2.1 × 50 mm Kinetex Biphenyl analytical column.

Peptide fractions of sufficient purity and correct molecular weight (verified MALDI-TOF and HPLC or LC–MS) were pooled and lyophilized. Concentrations were determined by UV absorption using the extinction coefficient at 280 nm, which was based on Trp/Tyr content.

### Minimum Inhibitory Concentration Growth Inhibition Assay

Bacterial stock was streaked onto LB agar plates and incubated at 37 °C for 16–18 h. Using a sterile swab, single colonies were picked and resuspended in 2 mL of 0.9% saline solution, visually matching the turbidity of a 0.5 McFarland standard and having an OD_600 nm_ of 0.18–0.25 (which corresponds to ∼5 × 10^5^ CFU/mL). The suspension was diluted 200-fold in cation-adjusted Mueller–-Hinton Broth (Ca-MHB), and 100 μL of the diluted culture was pipetted in wells of a 96-well plate containing 100 μL of a 2-factor dilution series of a compound per row of wells. The plate was incubated at 37 °C for 19 h. Afterward, the OD_600 nm_ of each well was read using a Pherastar FSX plate reader. MIC values were determined, with the MIC being the lowest concentration of the compound at which growth was absent (i.e., OD_600 nm_ was equivalent to no growth, as observed in carbenicillin control wells). This was also verified visually. All MIC ranges given are the result of values recorded by at least 3 biological repeats.

### *N*-Phenyl-1-naphthylamine (NPN) Assay

NPN assay was carried out following the outline of previously published methods.^5^ From a stock, bacteria were streaked onto LB agar plates and incubated at 37 °C for 16–18 h. Colonies were picked and incubated overnight in 5 mL of Ca-MHB at 37 °C. Next day, the overnight bacterial culture was diluted 1/100 and incubated (37 °C, 180 rpm, 3–3.5 h) to OD_600 nm_ of 1.0 to reach exponential growth phase. The culture was then pelleted, washed twice in HMG-Cl (50 mM HEPES-NaOH, 1 mM MgCl_2_, 0.4 mM glucose, pH 7.0), and resuspended in HMG-Cl containing 800 μM NPN (Acros). Finally, 50 μL of the bacterial suspension was added to black polysterene, clear bottom, 96-well plates (Corning, CellBIND 3340), with each well containing 50 μL of a compound. Fluorescence was measured using a Pherastar FSX plate reader (optic module FI 360 460, top optic), with readings occurring every 49 s for 150 cycles. The equation: NPN uptake (%) = (*F*_obs_*– F*_0_)/(*F*_PMBN_*– F*_0_) × 100% was used to calculate the %NPN uptake (see Figure 3). *F*_obs_ represents the fluorescence observed at a given peptide concentration, *F*_0_ represents the initial fluorescence in the absence of a compound, and *F*_PMBN_ represents the fluorescence observed for 50 μM polymyxin B nonapeptide (PMBN). All fluorescence values were recorded at 25 min. All fluorescence parameters were averages of three biological repeats.

### Circular Dichroism

Compounds dissolved in 50% (v/v) acetonitrile–water were diluted to 0.15 mg/mL in 10 mM sodium phosphate buffer, pH 7.0, with or without 10 mM SDS. 400 μL was then added to a 1 mm cuvette. Spectra were collected using the Aviv 410 circular dichroism instrument (25 °C, 5 s averaging time, 3 scans per compound, 1 nm wavelength step, wavelength range 190–250 nm). Using the accompanying software (Aviv Biomedical Inc., CDS version 3.20), the traces of each compound were averaged, the buffer signal was subtracted, and the resulting trace was subjected to smoothing (degree: 2, window width: 11). Percentage helicity (% helicity, Table 3) was generated by inputting the negative (correction for the d*-*amino acid ellipticity) of the molar circular dichroism values (Δε) of each molecule into the online secondary structure prediction tool K2D3 (http://cbdm-01.zdv.uni-mainz.de/ ∼ andrade/k2d3)^26^ for a wavelength range between 190 and 240 nm.

### Helical Wheel Projections and Hydrophobic Moment Calculations

Projections seen in Figure 2 were generated using the helical wheel projection software created by analyzing it with the online tool created by Don Armstrong and Raphael Zidovetzki (https://pss.sjtu.edu.cn/cgi-bin/wheel.cgi). Hydrophobic moments (μ_H_) were calculated using the Eisenberg equation:^37^
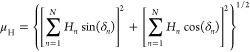


where *H*_*n*_ is the hydrophobicity index of residue *n,*(37) and δ is the angle, in radians, of the side chain of residue *n* when the helix is viewed down its axis. To make these calculations, a perfect helical structure was assumed, with each successive side chain rotating 100°.

## Supporting Information Available

NPN uptake results for MAP-Bicycle conjugates and their equivalent standalone MAP vectors, expressed in fluorescent units and %NPN uptake; comparison of NPN uptake to MIC values; CD spectra of SV2 MIC values recorded for all repeats for all compounds tested; details of bacterial strains used LCMS traces for compounds tested (PDF)
