# Supplementary material for: Investigating Penetration and Antimicrobial Activity of Vector-Bicycle Conjugates
Source: ACS Infect Dis. 2024 Jun 12;10(7):2381–9. doi: 10.1021/acsinfecdis.3c00427 (PMC11249977; doi:10.1021/acsinfecdis.3c00427)
Supplement: Supplementary file 1 — id3c00427_si_001.pdf [file id3c00427_si_001.pdf]

Supporting information

Investigating penetration and antimicrobial activity of vector-Bicycle conjugates

Andreas Hadjicharalambous[1,2], Hector Newman[2,3], Nick Lewis[2], Catherine Rowland[2], Nikolaos Bournakas[2], Steven J. Stanway[2], Michael Dawson[2], Michael J. Skynner[2] and Paul Beswick[2*]

[1] Department of Biochemistry, University of Cambridge, Cambridge CB2 1QN, UK

[2] BicycleTx Limited, Portway Building, Granta Park, Cambridge CB21 6GS, UK

[3] School of Life Sciences, University of Warwick, Coventry CV4 7AL, UK

**Corresponding author* (paul.beswick@bicycletx.com)

Supporting Figures

Figure S1 - Fluorescence timecourse experiments for MAP-Bicycle conjugates and their equivalent standalone MAP vectors

Figure S2 - NPN uptake of different MAP vectors and MAP-Bicycle conjugates. %NPN uptake for MAP vectors or their equivalent MAP-Bicycle conjugate

Figure S3 - CD spectra for the MAP vector SV2

Figure S4 - Comparison of the average MIC and %NPN uptake of MAP-Bicycle conjugates

Supporting Tables

Table S1 - All MIC values recorded for all repeats for all compounds tested

Table S2 – Strains Used

Compound Quality control data

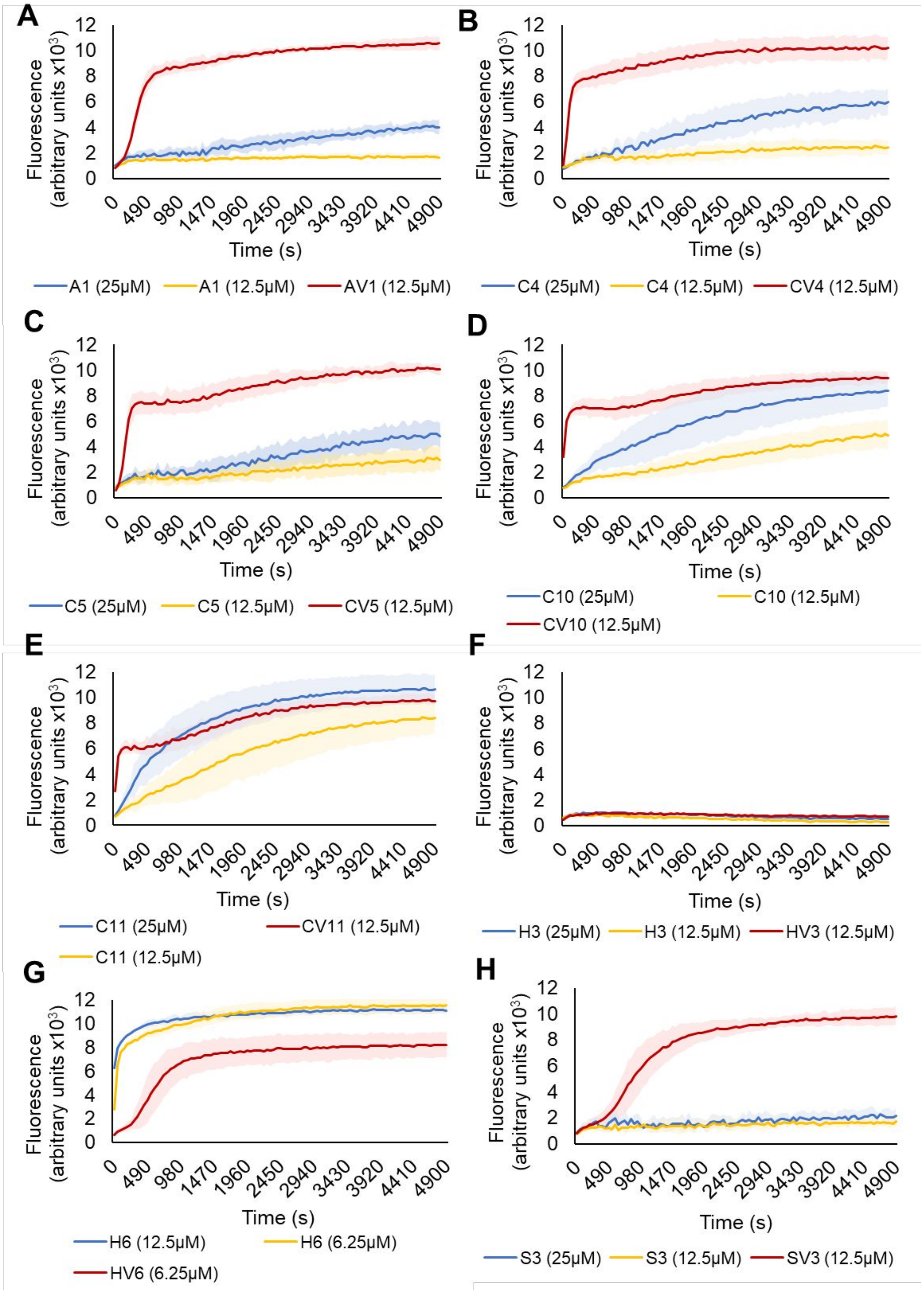

Figure S1: Fluorescence timecourse of an NPN uptake experiment for MAP-Bicycle conjugates and their equivalent standalone MAP vectors; **(A)** A1/AV1, **(B)** C4/CV4, **(C)** C5/CV5, **(D)** C10/CV10, **(E)** C11/CV11, **(F)** H3/HV3, **(G)** H6/HV6, **(H)** S3/SV3. The standalone vector is shown in red, while the MAP-Bicycle conjugate is shown in blue (same concentration as the MAP vector) or in yellow (double the concentration as the vector). Error bars surrounding each line represent standard deviation (n=3).

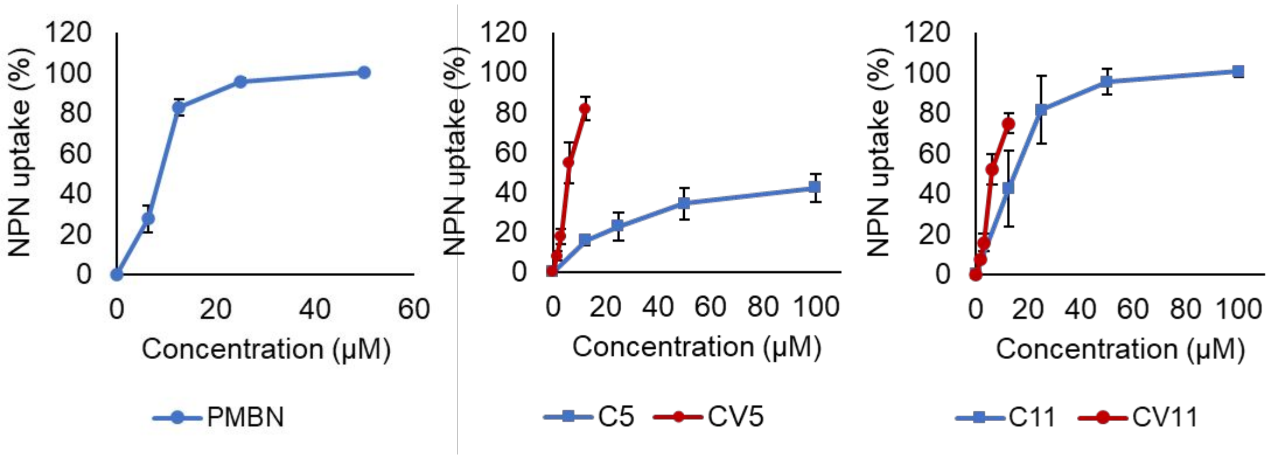

Figure S2: NPN uptake of different MAP vectors and MAP-Bicycle conjugates. %NPN uptake for MAP vectors or their equivalent MAP-Bicycle conjugate; **(Left)** PMBN, **(Centre)** C5/CV5, **(Right)** C10/CV10. The standalone MAP vector is in red while the MAP-Bicycle conjugate is in blue. Error bars represent the standard deviation (n=3).

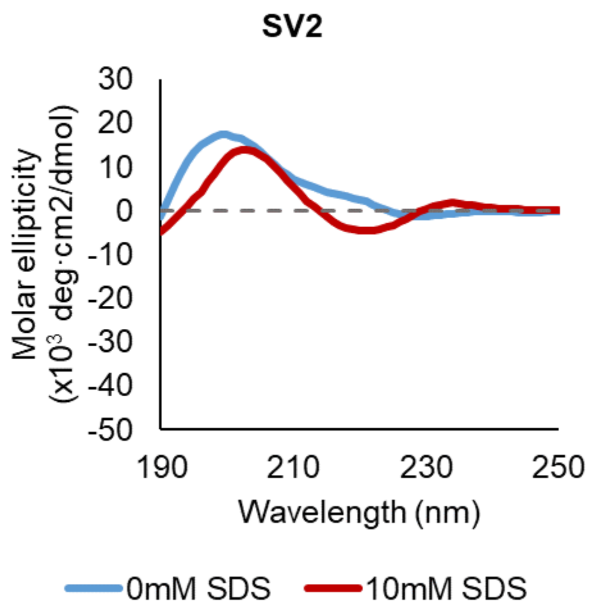

Figure S3: CD spectra for the MAP vector SV2 measured at a concentration of 0.15mg/ml in a 10Mm Sodium Phosphate buffer, Ph 7.0. The spectra were measured in either 0mM (blue line) or 10mM SDS (red line). (blue line) or 10mM SDS (red line).

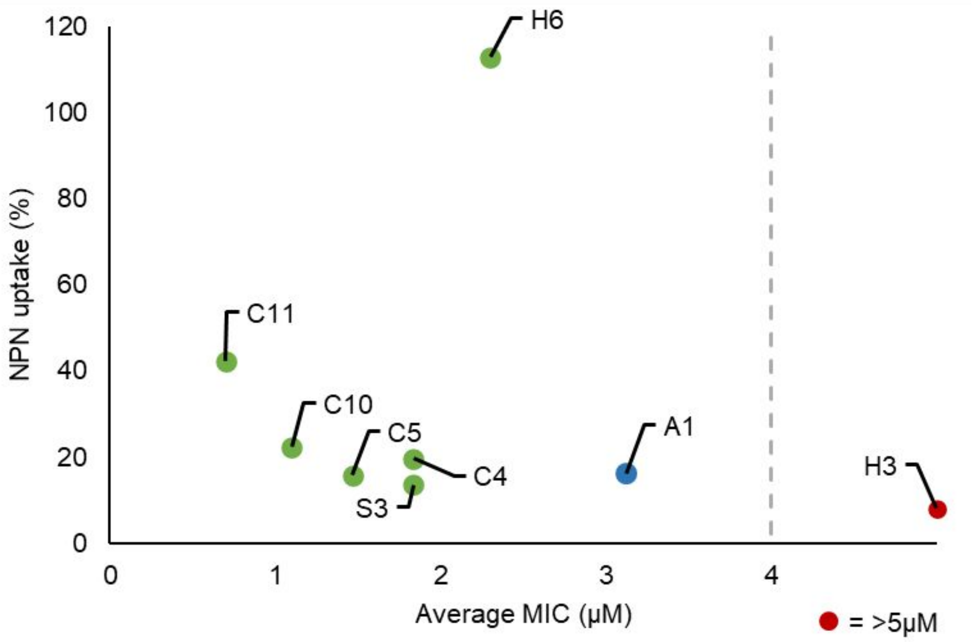

Figure S4: Comparison of the average MIC and %NPN uptake of MAP-Bicycle conjugates (n=3). The parent MAP-Bicycle conjugate is shown in blue while the variants are shown in green. H3 has an MIC beyond 5µM and is shown in red.

Table S1: All MIC values recorded for all repeats for all compounds tested.

Compound name			MIC (µg/ml)		
			**Bicycle**		
AB1			**>64**		
**Compound name**	**MIC (µg/ml)**	**Compound name**	**MIC (µg/ml)**	**Compound name**	**MIC (µg/ml)**
**MAP-Bicycle conjugate**		**MAP**		**MAP-*D*-Bicycle**	
A1	**8,8,16,8,16**	AV1	**32,32,32**	**-**	**-**
C1	**4,4,16**	CV1	**>64,>64,>64**	**-**	**-**
C2	**4,4,16**	CV2	**16,16,16**	CD2	**>64,>64,>64**
C3	**8,8,4**	CV3	**>64,>64,>64**	**-**	-
C4	**4,8,8**	CV4	**32,32,32**	CD4	**>64,>64,>64**
C5	**8,4,4**	CV5	**32,32,32**	CD5	**>64,>64,>64**
C6	**4,8,32**	CV6	**16,16,16**	**-**	**-**
C7	**32,32,8**	-	-	**-**	**-**
C8	**4,4,4**	CV8	**64,64,>64**	**-**	**-**
C9	**8,8,4**	CV9	**64,64,>64**	**-**	**-**
C10	**4,4,4**	CV10	**>64,>64,>64**	**-**	**-**
C11	**2,4,2**	CV11	**64,32,64**	CD11	**>64,>64,>64**
C12	**2,4,32**	CV12	**16,16,16**	**-**	**-**
H1	**64,>64,>64**	HV1	**>64,>64,>64**	**-**	**-**
H2	**>64,>64,>64**	HV2	**>64,>64,>64**	**-**	**-**
H3	**>64,32,64**	HV3	**>64,>64,>64**	**-**	**-**
H4	**64,>64,>64**	HV4	**>64,>64,>64**	**-**	**-**
H5	**32,16,16**	HV5	**>64,>64,>64**	**-**	**-**
H6	**8,8,8**	HV6	**>64,>64,>64**	**-**	**-**
S1	**32,32,32**	SV1	**>64,>64,>64**	**-**	**-**
S2	**8,8,8**	SV2	**32,32,32**	SD2	**>64,>64,>64**
S3	**4,8,8**	SV3	**16,16,16**	SD3	**>64,>64,>64**
S4	**8,8,8**	SV4	**32,32,64**	SD4	**>64,>64,>64**

Table S2: Strains used

Name	Species	Description	Source
ATCC25922	Escherichia coli	Clinical Isolate	ATCC LGC STANDARDS

Compound Quality control data

Linear vectors chromatographic and mass spectrometry data

SV4

Area Percent Report

Agilent Technologies

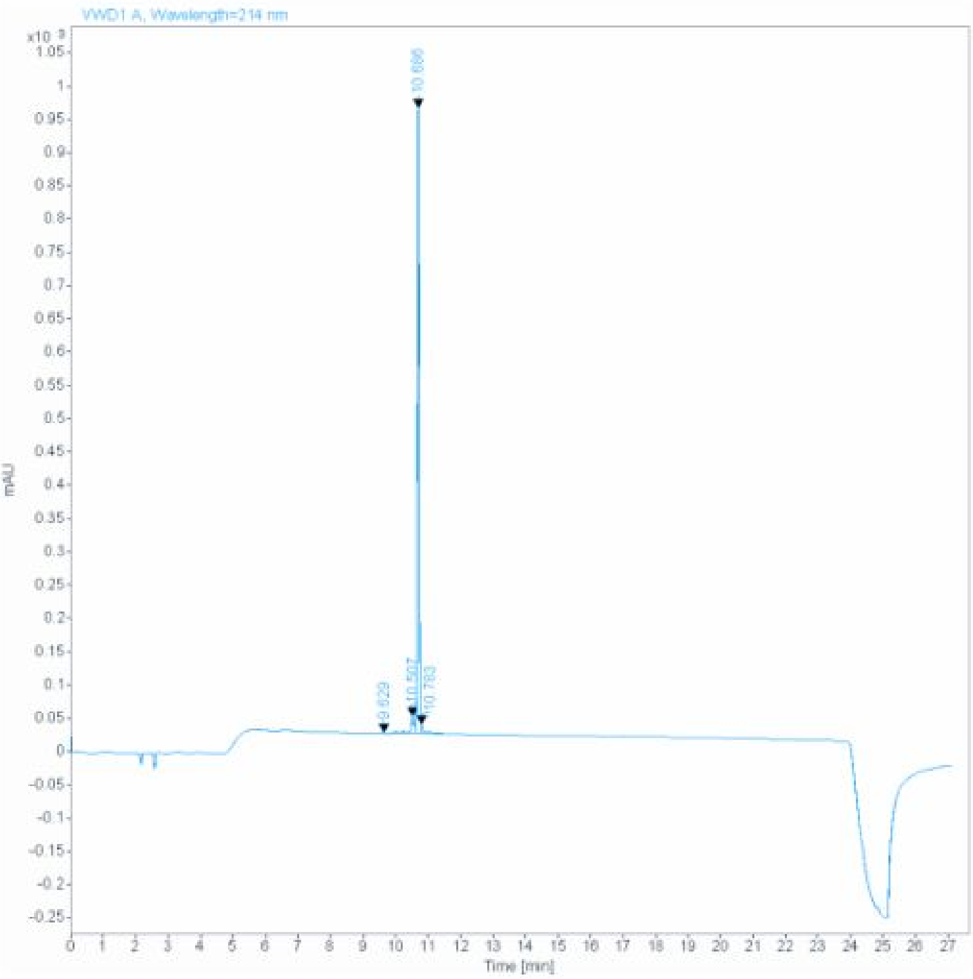

Signal: VWD1 A, Wavelength=214 nm

RT [min]	Type	Width [min]	Area	Height	Area%	Name
9.629	MM	0.0544	2.8665	0.8783	0.0718	
10.507	MF	0.1621	256.1739	26.3446	6.4169	
10.686	MF	0.0646	3652.2065	942.3068	91.4848	
10.783	FM	0.0945	80.8990	14.2708	2.0265	
		Sum	3992.1459			

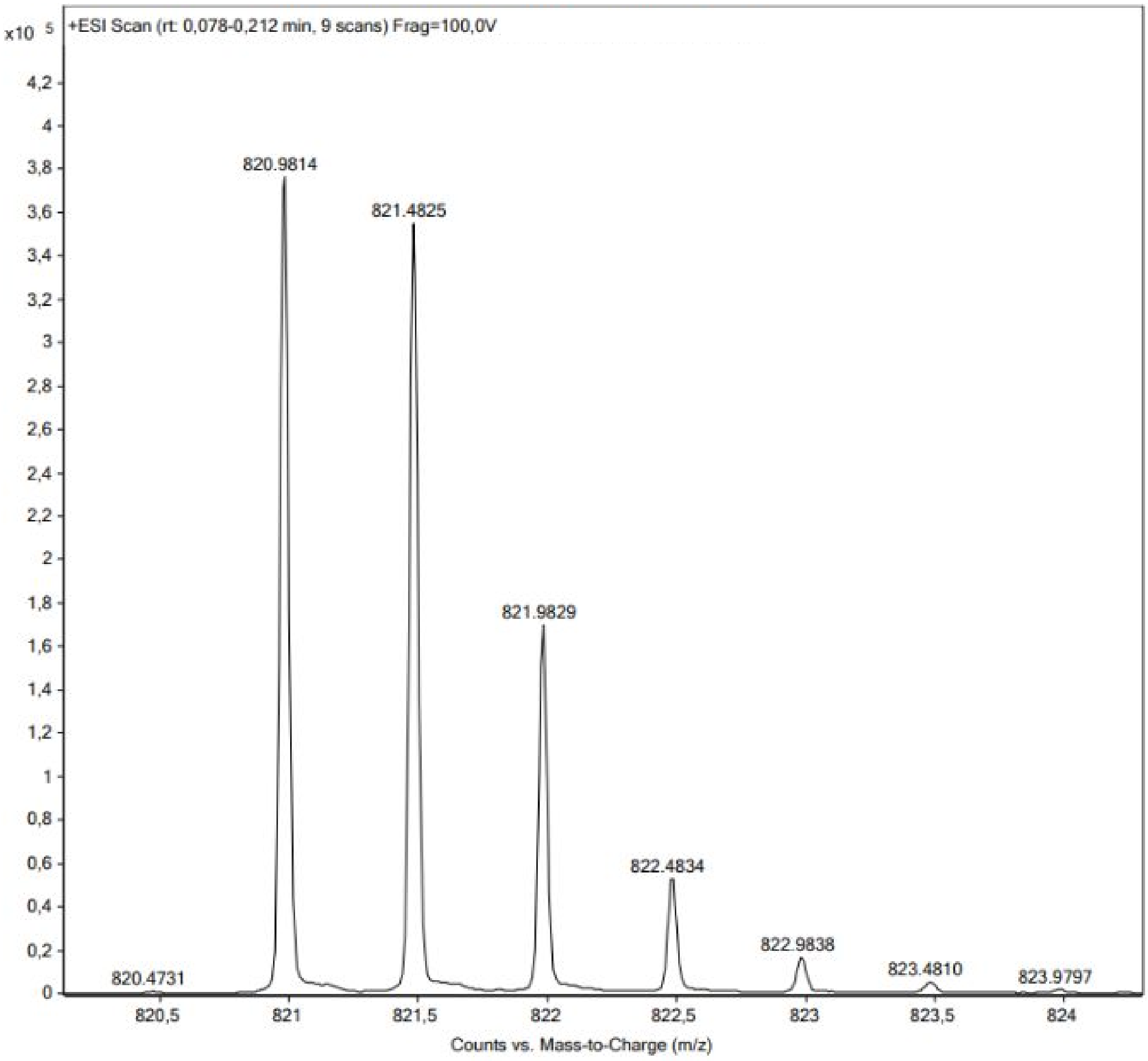
x10 5
+ESI Scan (rt: 0,078-0,212 min, 9 scans) Frag=100,0V
4,2
4
3,8
3,6
3,4
3,2
3
2,8
2,6
2,4
2,2
2
1,8
1,6
1,4
1,2
1
0,8
0,6
0,4
0,2
0
820.9814
821.4825
821.9829
822.4834
822.9838
823.4810
823.9797
820.4731
820,5
821
821,5
822
822,5
823
823,5
824
Counts vs. Mass-to-Charge (m/z)

SV3

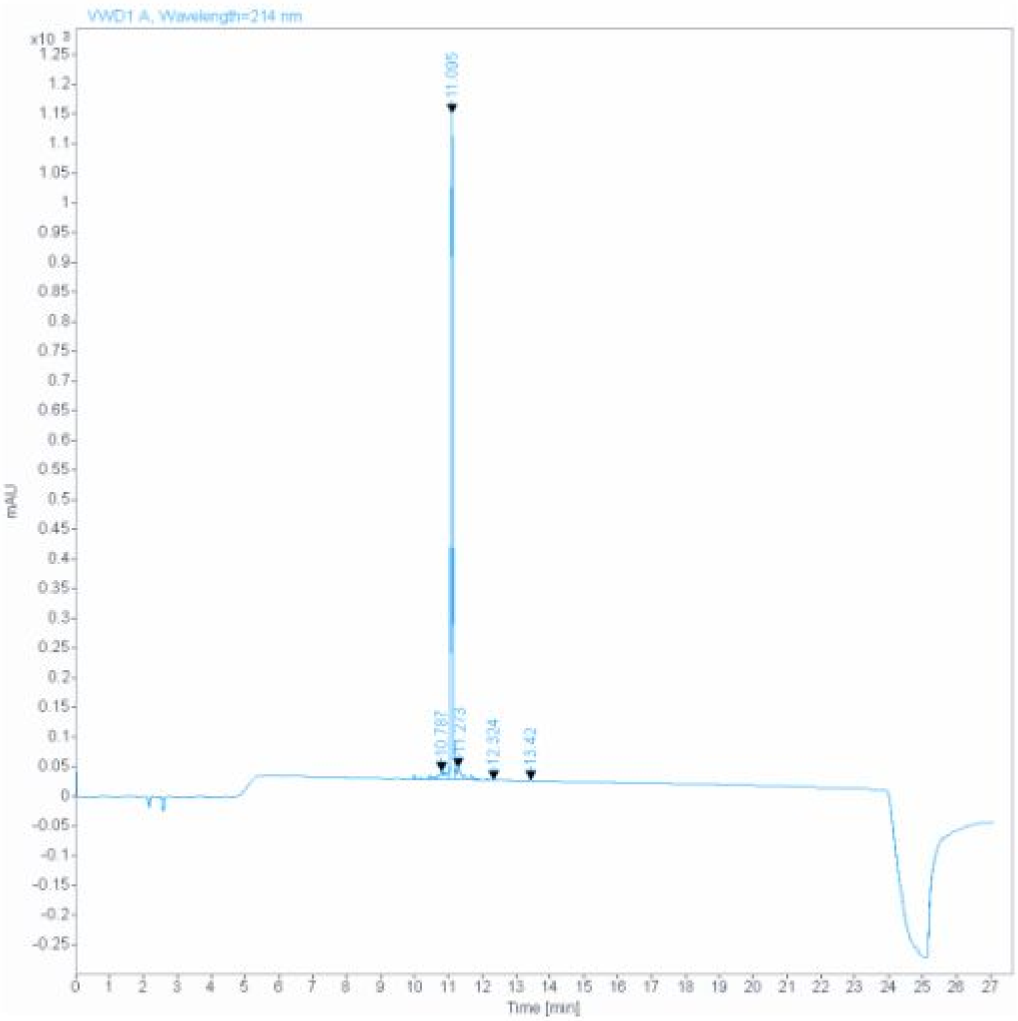

Signal: VWD1 A, Wavelength=214 nm

RT [min]	Type	Width [min]	Area	Height	Area%	Name
10.787	MF	0.3460	289.7846	13.9576	5.7788	
11.095	FM	0.0656	4437.8496	1127.2886	88.4982	
11.273	FM	0.2166	275.3800	21.1894	5.4915	
12.324	MM	0.1433	6.3193	0.7348	0.1260	
13.420	MM	0.0553	5.2894	1.5941	0.1055	
		Sum	5014.6230			

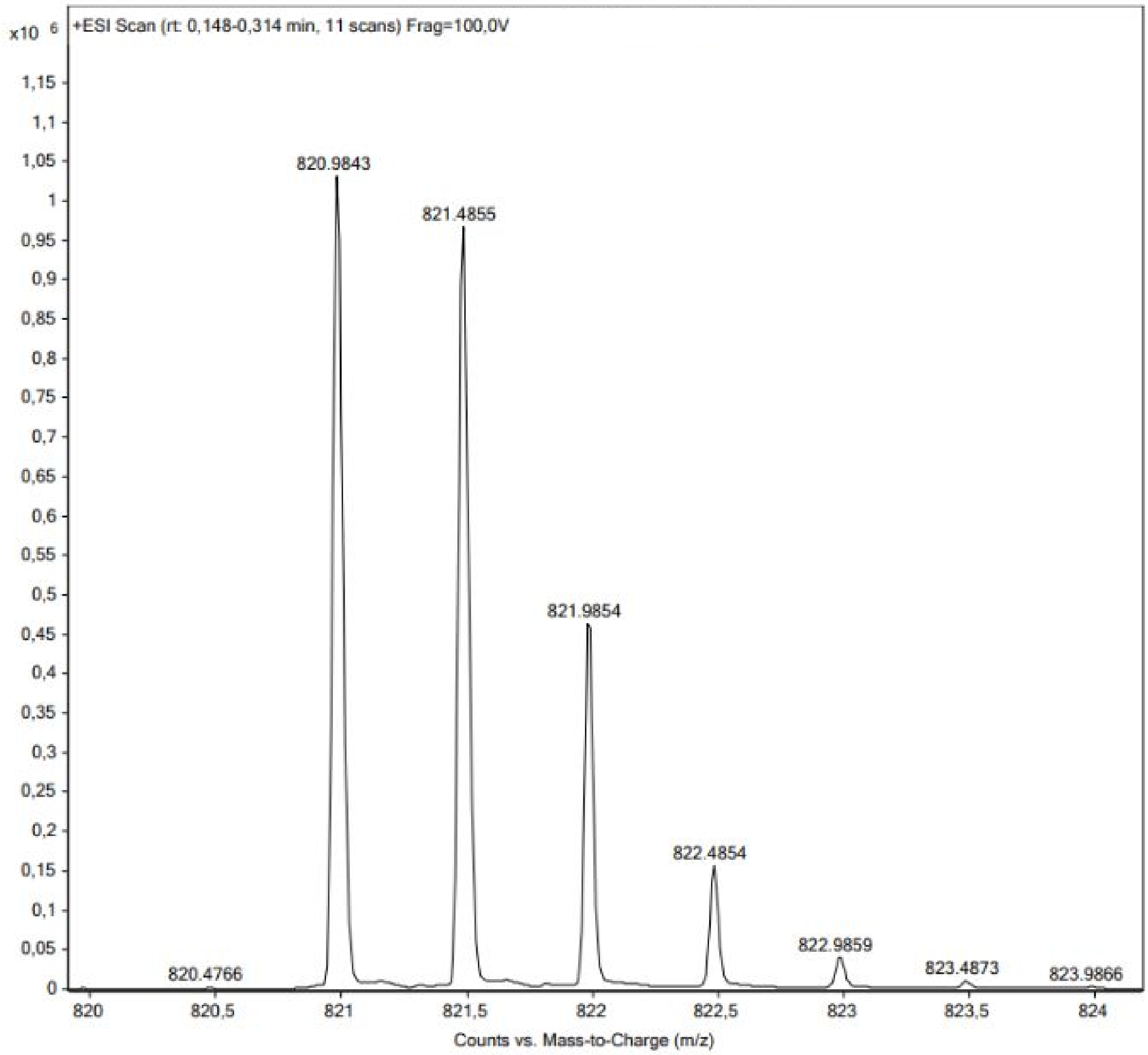

x10 6
+ESI Scan (rt: 0,148-0,314 min, 11 scans) Frag=100,0V
820.9843
821.4855
821.9854
822.4854
822.9859
823.4873
823.9866
820.4766
Counts vs. Mass-to-Charge (m/z)

SV2

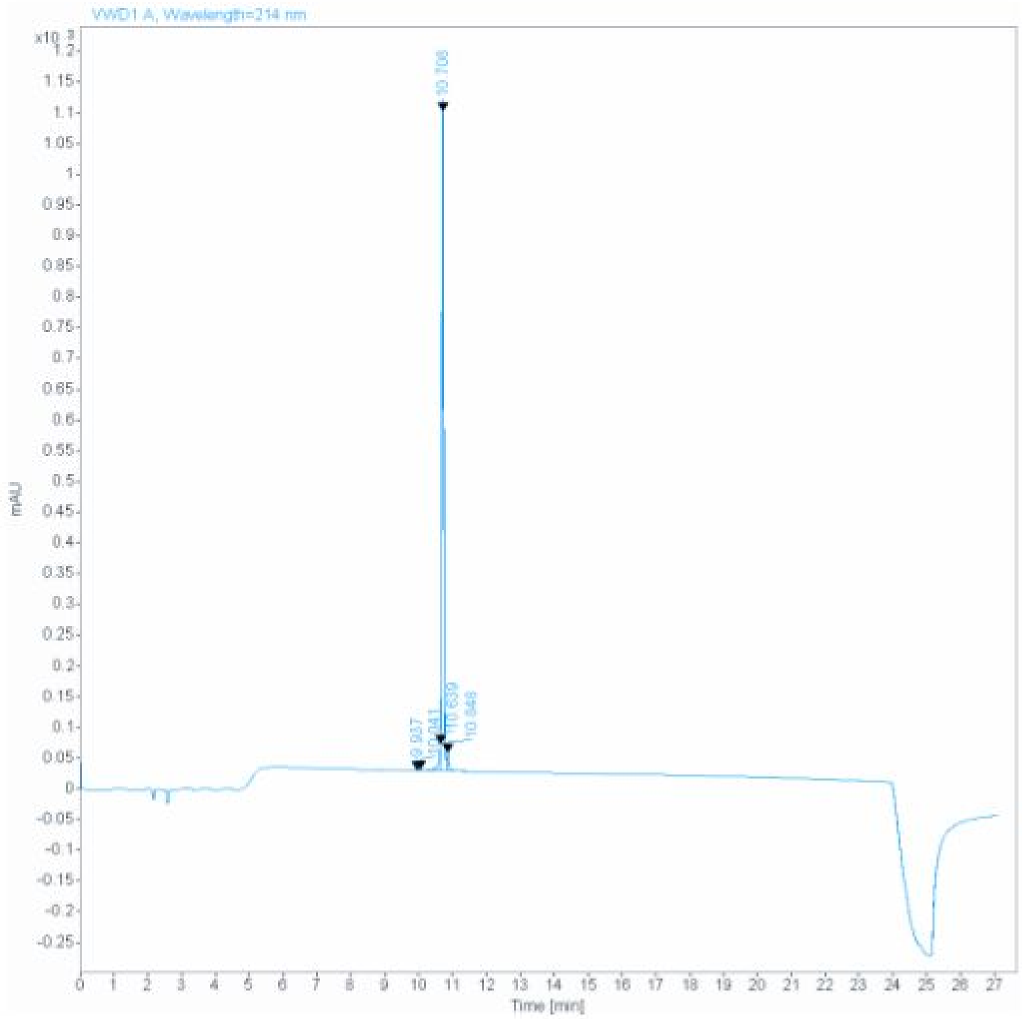

Signal: VWD1 A, Wavelength=214 nm

RT [min]	Type	Width [min]	Area	Height	Area%	Name
9.937	MM	0.0400	1.4446	0.6017	0.0336	
10.041	MM	0.1010	10.3592	1.7089	0.2407	
10.639	MF	0.0571	150.2152	43.8494	3.4896	
10.708	MF	0.0621	4007.5161	1075.7657	93.0971	
10.848	FM	0.0754	135.1254	29.8521	3.1390	
		Sum	4304.6606			

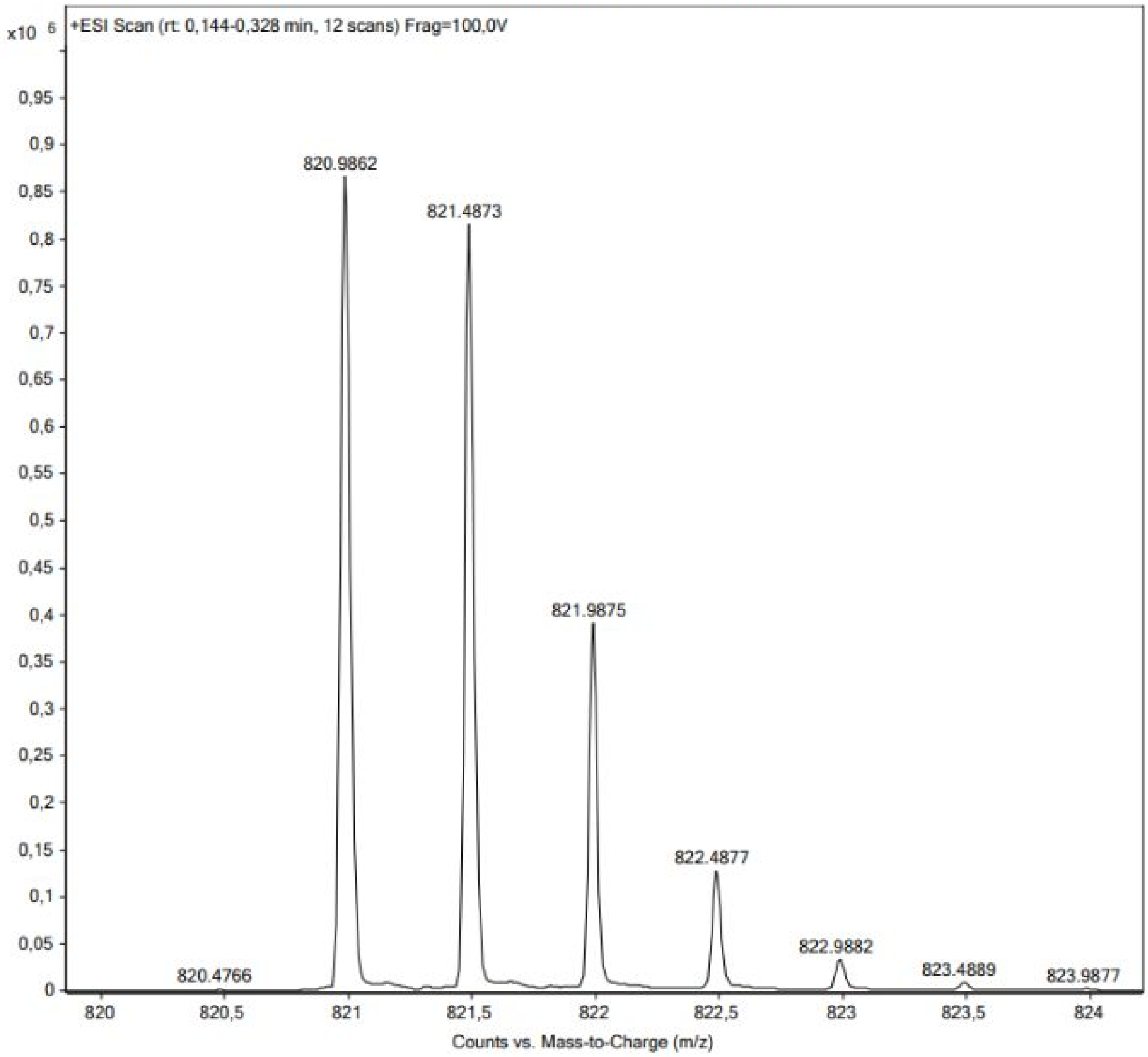
x10 6
+ESI Scan (rt: 0,144-0,328 min, 12 scans) Frag=100,0V
0,95
0,9
0,85
0,8
0,75
0,7
0,65
0,6
0,55
0,5
0,45
0,4
0,35
0,3
0,25
0,2
0,15
0,1
0,05
0
820.9862
821.4873
821.9875
822.4877
822.9882
823.4889
823.9877
820.4766
820
820,5
821
821,5
822
822,5
823
823,5
824
Counts vs. Mass-to-Charge (m/z)

CV7

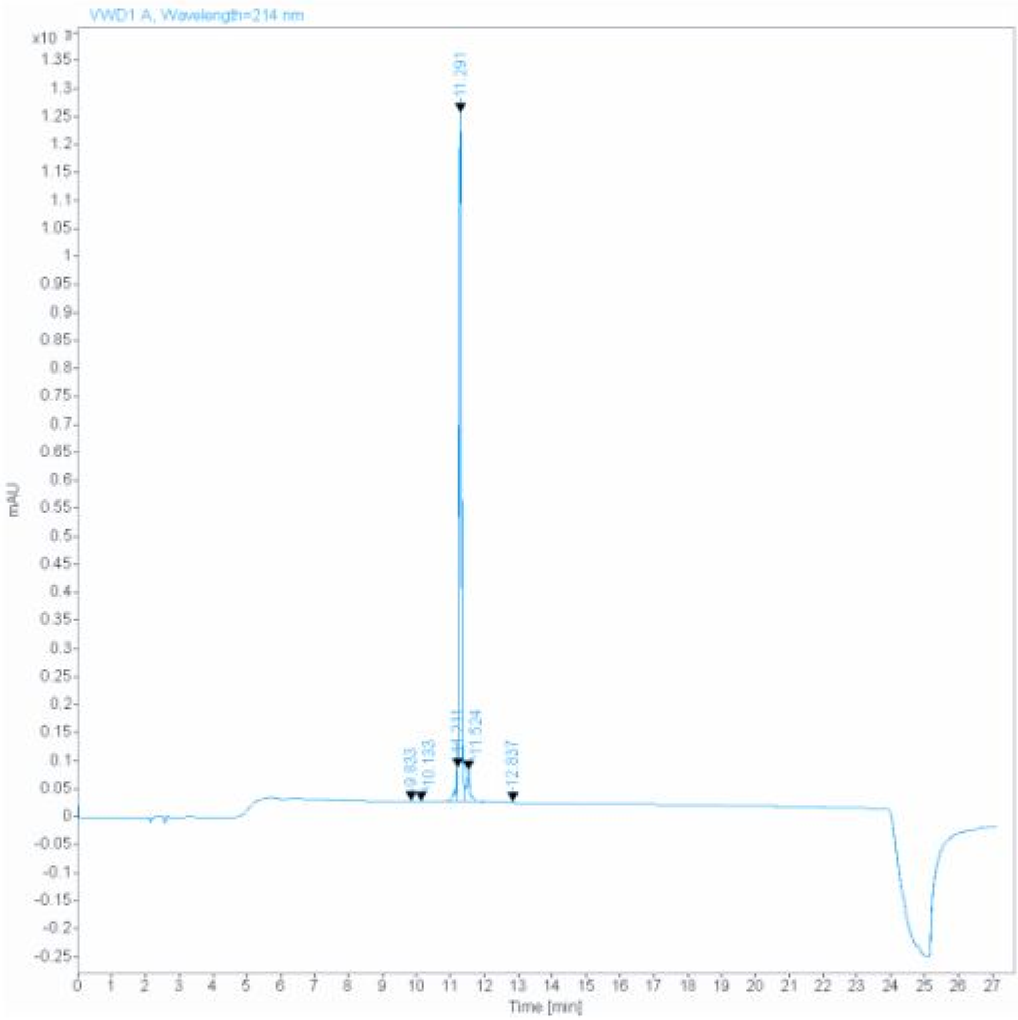

Signal: VWD1 A, Wavelength=214 nm

RT [min]	Type	Width [min]	Area	Height	Area%	Name
9.833	MM	0.0722	7.1187	1.6424	0.1069	
10.133	MM	0.1096	9.2356	1.4042	0.1387	
11.211	MF	0.0837	310.7613	61.8692	4.6655	
11.291	MF	0.0796	5891.5591	1233.7303	88.4509	
11.524	FM	0.1254	434.1534	57.7074	6.5180	
12.837	MM	0.0528	7.9975	2.5231	0.1201	
		Sum	6660.8256			

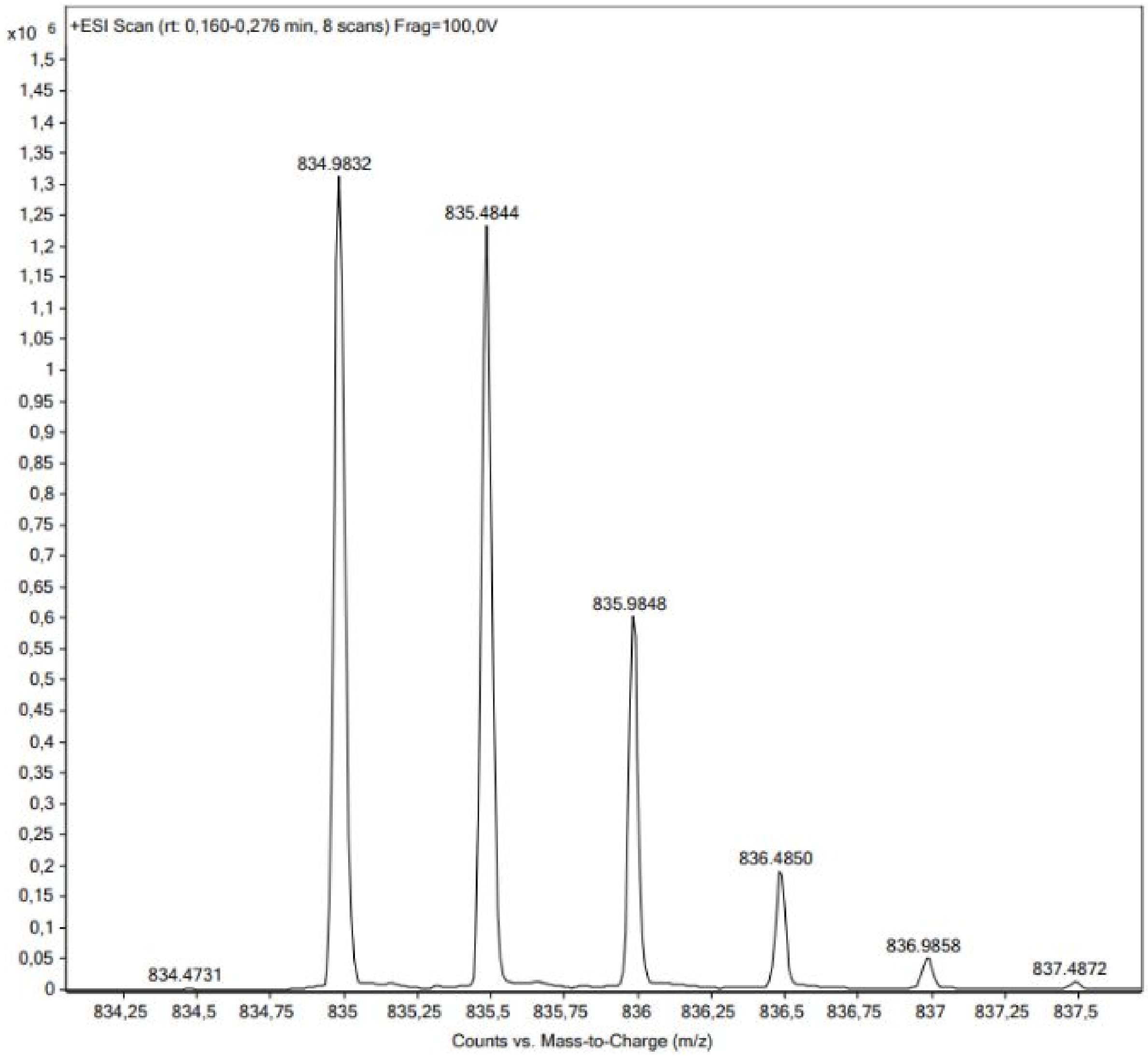
x10 6
+ESI Scan (rt: 0,160-0,276 min, 8 scans) Frag=100,0V
834.9832
835.4844
835.9848
836.4850
836.9858
837.4872
834.4731
Counts vs. Mass-to-Charge (m/z)

CV1

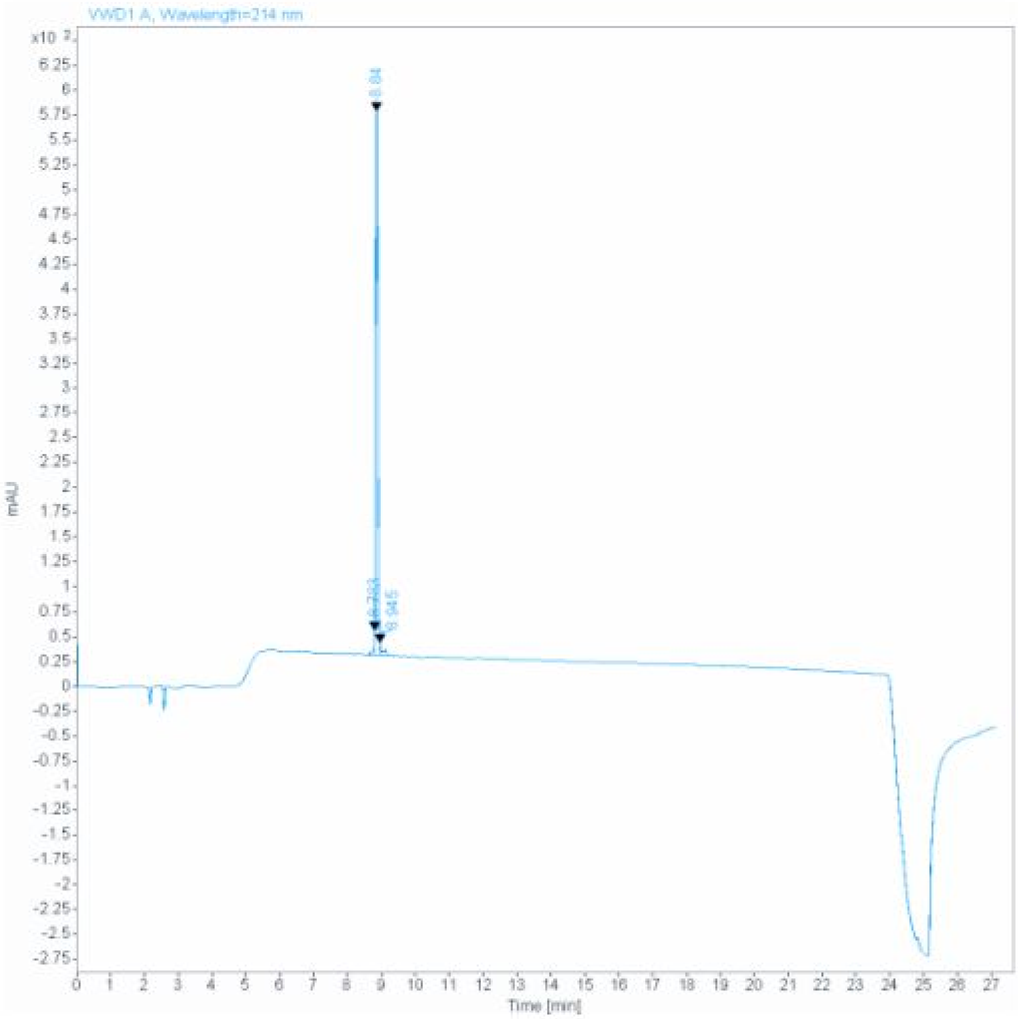

Signal: VWD1 A, Wavelength=214 nm

RT [min]	Type	Width [min]	Area	Height	Area%	Name
8.783	MF	0.0289	42.1761	24.3453	1.5672	
8.840	MF	0.0784	2581.0601	548.5083	95.9085	
8.945	FM	0.0784	67.9323	11.4747	2.5243	
		Sum	2691.1684			

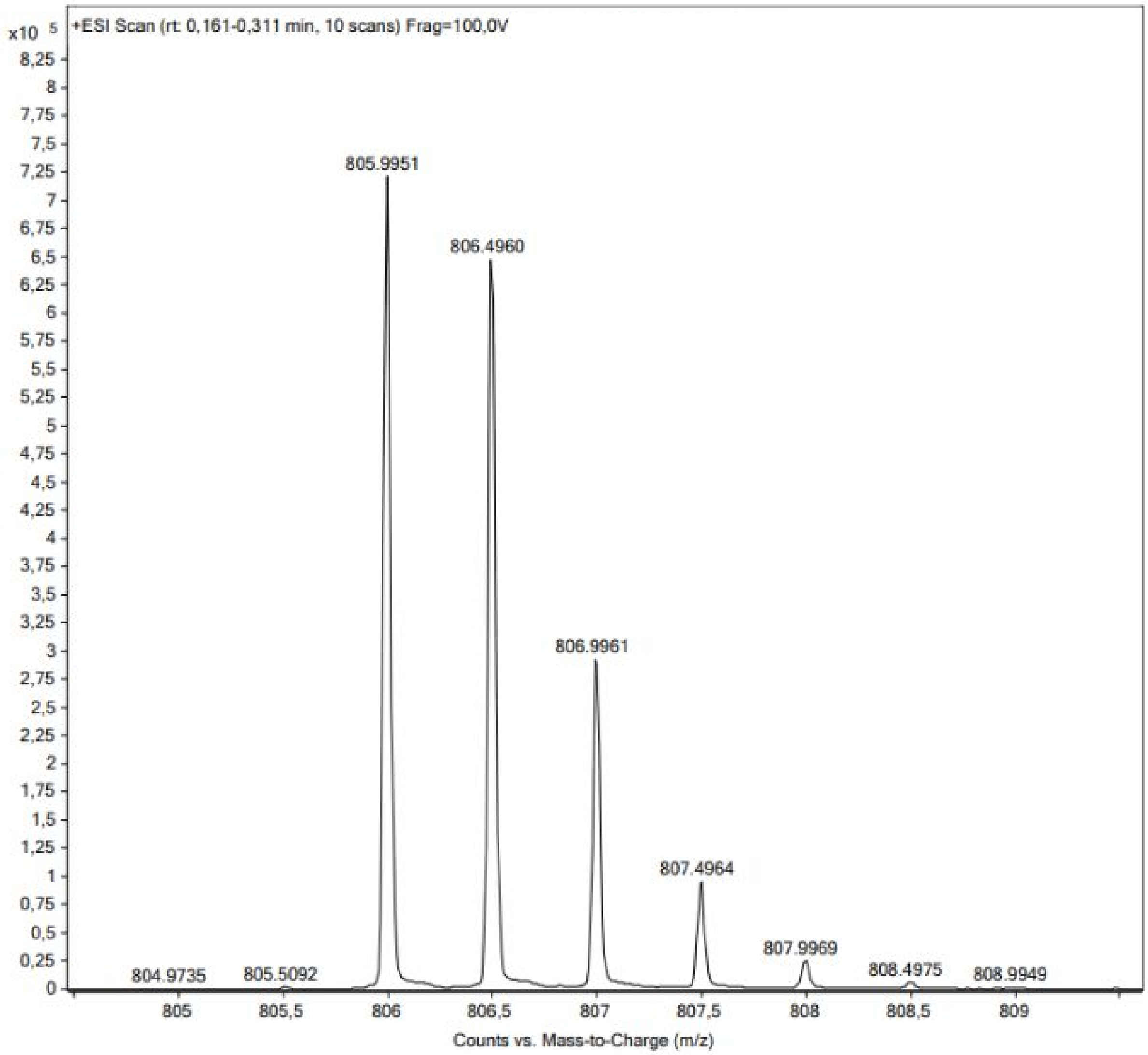

x10 5
+ESI Scan (rt: 0,161-0,311 min, 10 scans) Frag=100,0V
805.9951
806.4960
806.9961
807.4964
807.9969
808.4975
808.9949
804.9735
805.5092
Counts vs. Mass-to-Charge (m/z)

CV2

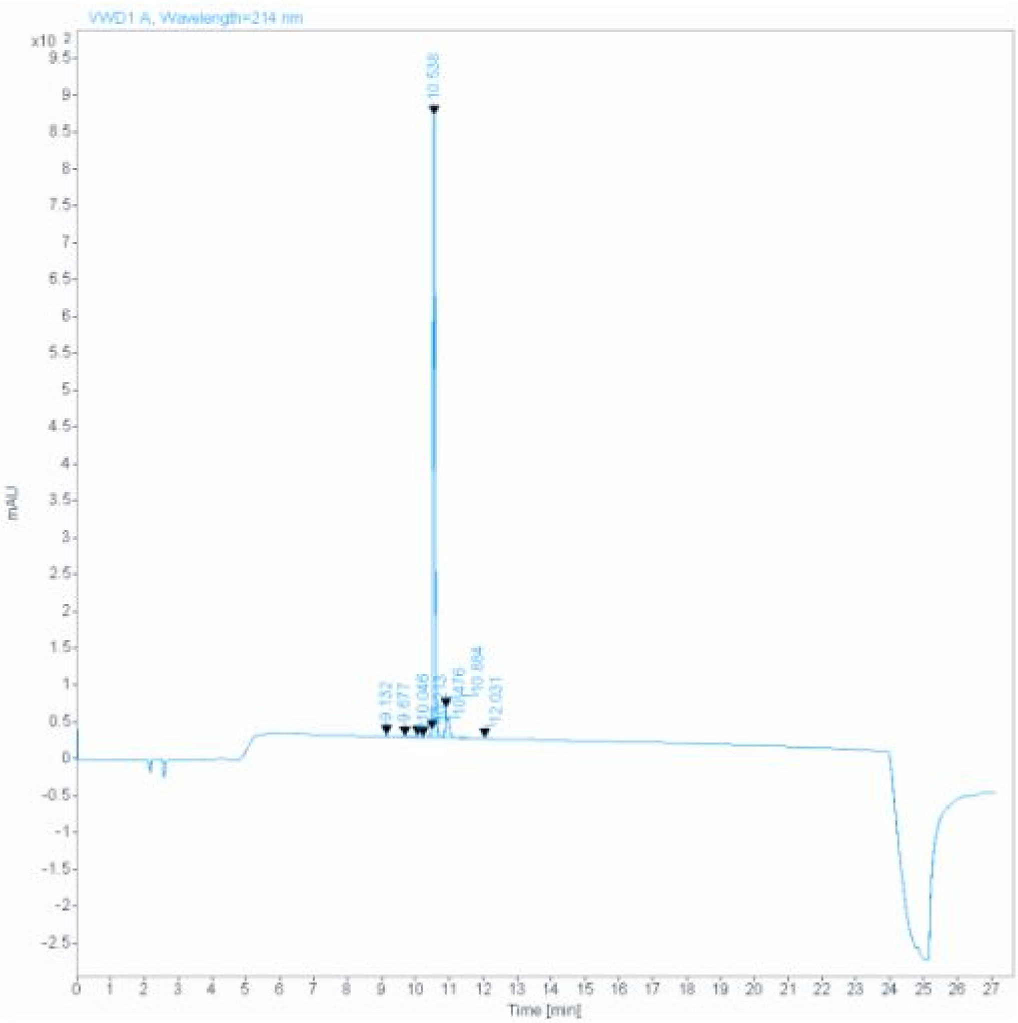

Signal: VWD1 A, Wavelength=214 nm

RT [min]	Type	Width [min]	Area	Height	Area%	Name
9.132	MM	0.1372	28.1798	3.4240	0.7016	
9.677	MM	0.0739	9.6299	2.1716	0.2398	
10.046	MM	0.1053	16.6696	2.6380	0.4150	
10.213	MM	0.0907	9.0658	1.6659	0.2257	
10.476	MF	0.0691	46.4915	11.2167	1.1576	
10.538	FM	0.0703	3572.8723	846.9645	88.9586	
10.884	FM	0.1323	326.9245	41.1716	8.1399	
12.031	MM	0.0677	6.4985	1.6002	0.1618	
		Sum	4016.3319			

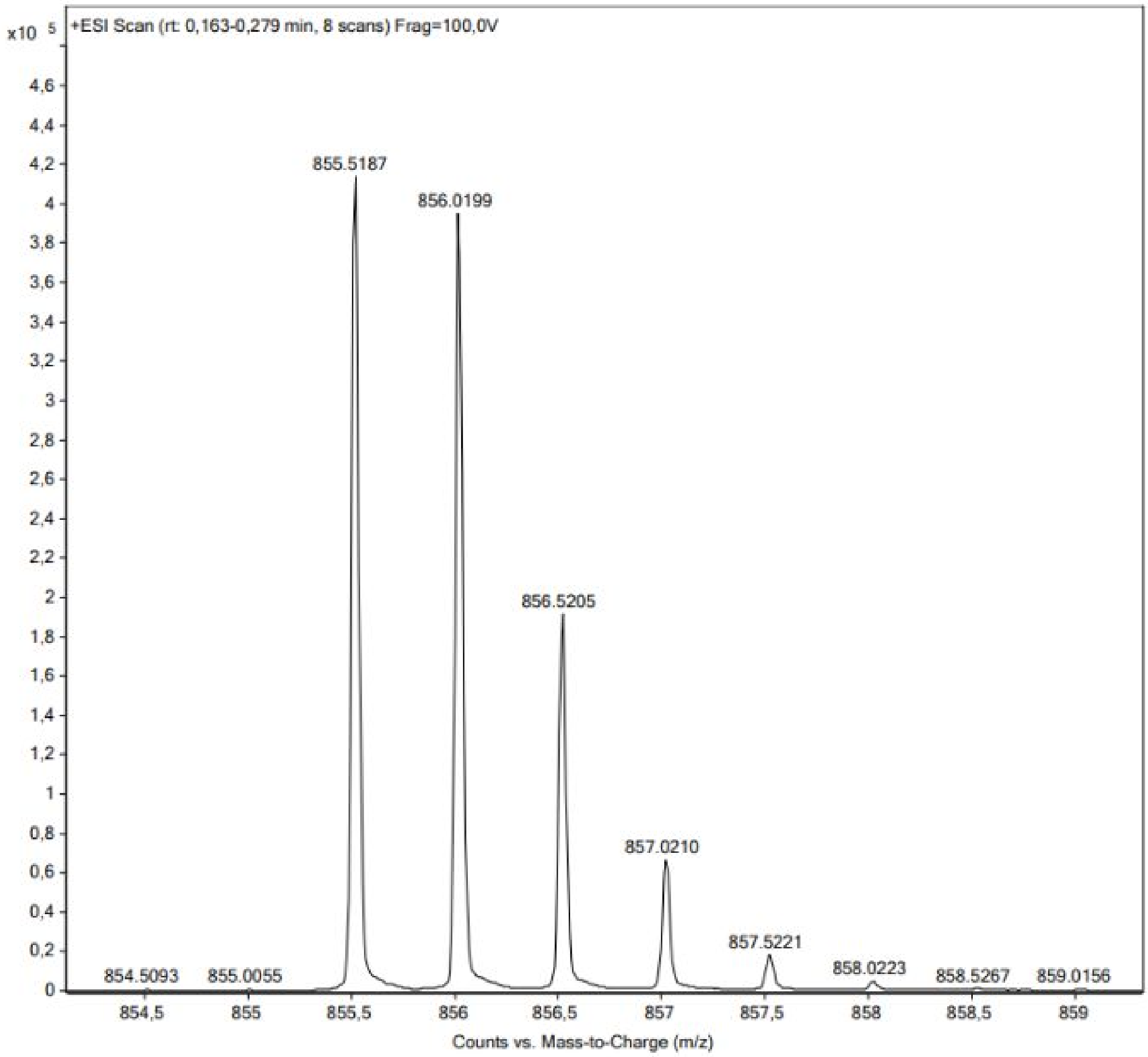

x10 5
+ESI Scan (rt: 0,163-0,279 min, 8 scans) Frag=100,0V
855.5187
856.0199
856.5205
857.0210
857.5221
858.0223
858.5267
859.0156
854.5093
855.0055
Counts vs. Mass-to-Charge (m/z)

CV3

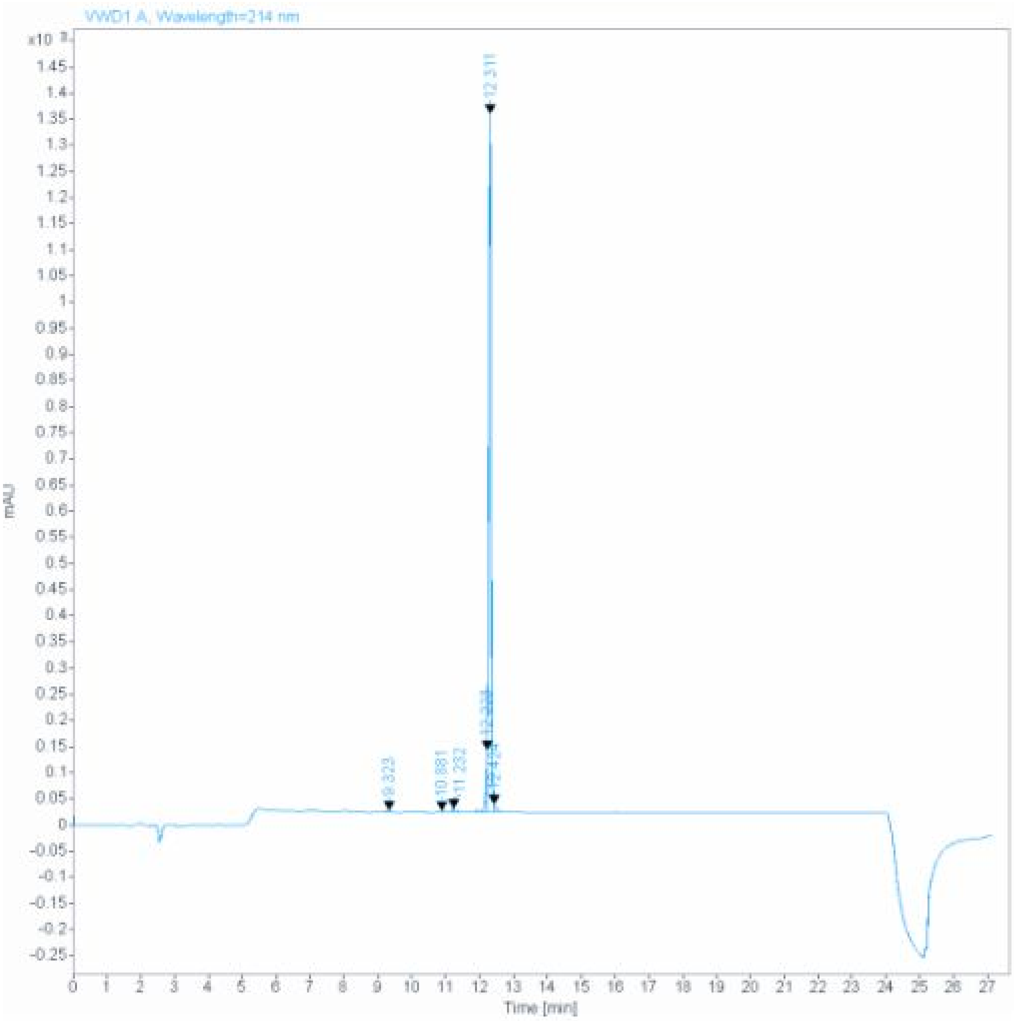

Signal: VWD1 A, Wavelength=214 nm

RT [min]	Type	Width [min]	Area	Height	Area%	Name
9.323	MM	0.0825	16.0383	3.2390	0.2450	
10.881	MM	0.0593	11.8601	3.3329	0.1812	
11.232	MM	0.1195	52.8511	7.3725	0.8075	
12.223	MF	0.0410	291.6785	118.5406	4.4565	
12.311	MF	0.0757	6092.1465	1341.2595	93.0804	
12.424	FM	0.0890	80.4628	15.0722	1.2294	
		Sum	6545.0372			

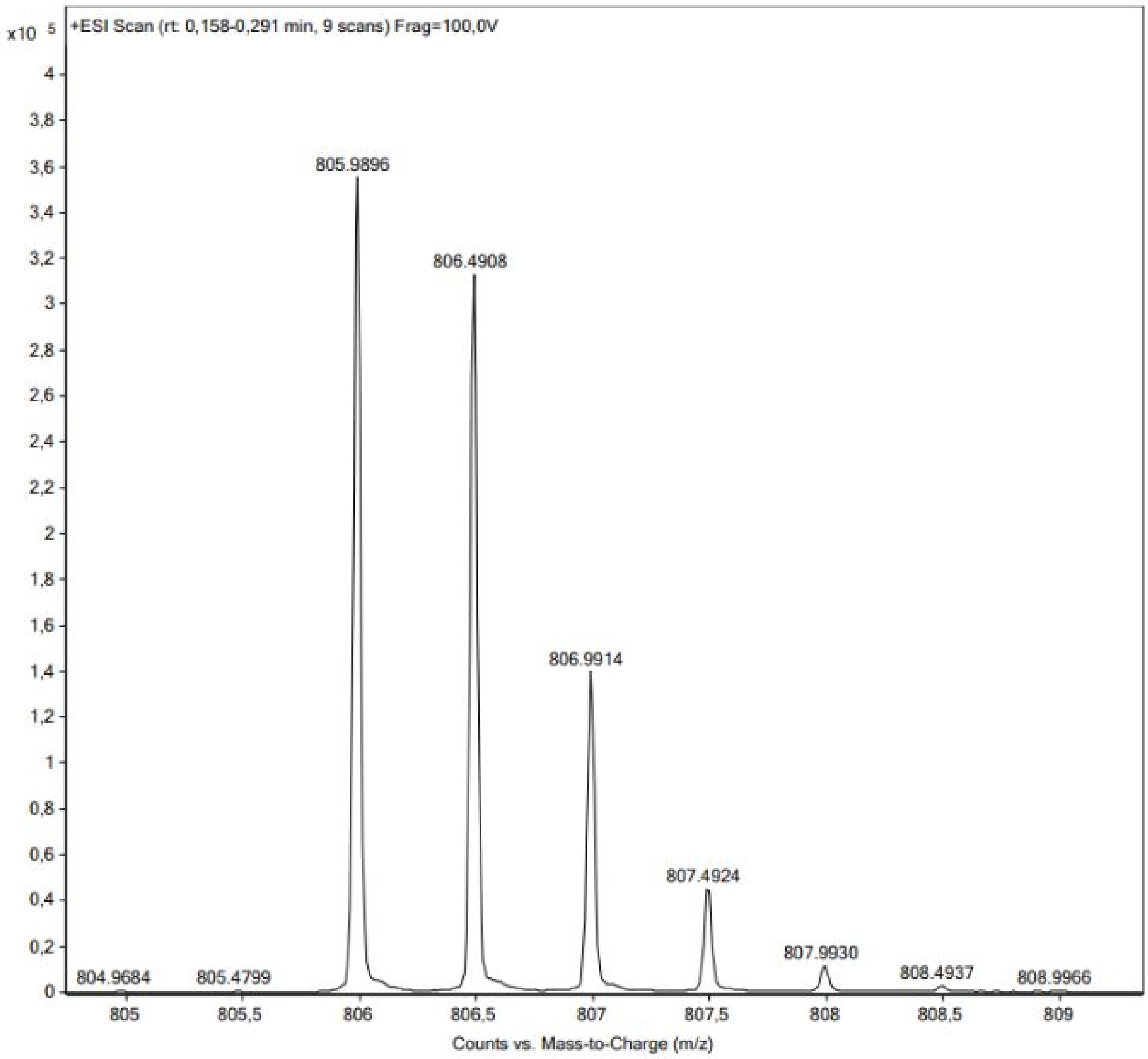

x10 5
+ESI Scan (rt: 0,158-0,291 min, 9 scans) Frag=100,0V
805.9896
806.4908
806.9914
807.4924
807.9930
808.4937
808.9966
804.9684
805.4799
Counts vs. Mass-to-Charge (m/z)

CV4

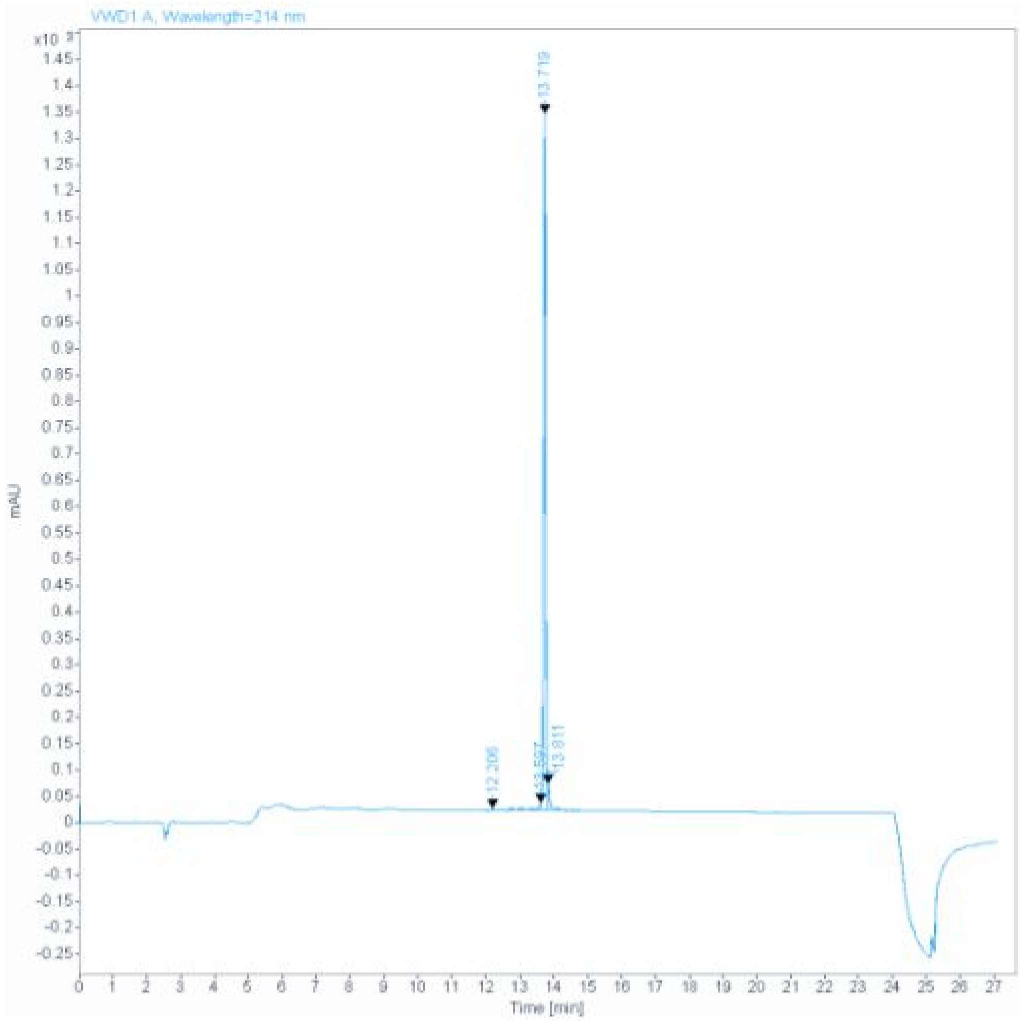

Signal: VWD1 A, Wavelength=214 nm

RT [min]	Type	Width [min]	Area	Height	Area%	Name
12.206	MM	0.1527	37.2201	4.0616	0.5841	
13.597	MF	0.2335	211.3662	15.0875	3.3168	
13.719	MF	0.0732	5826.7915	1327.4868	91.4364	
13.811	FM	0.0969	297.1312	51.1038	4.6627	
		Sum	6372.5090			

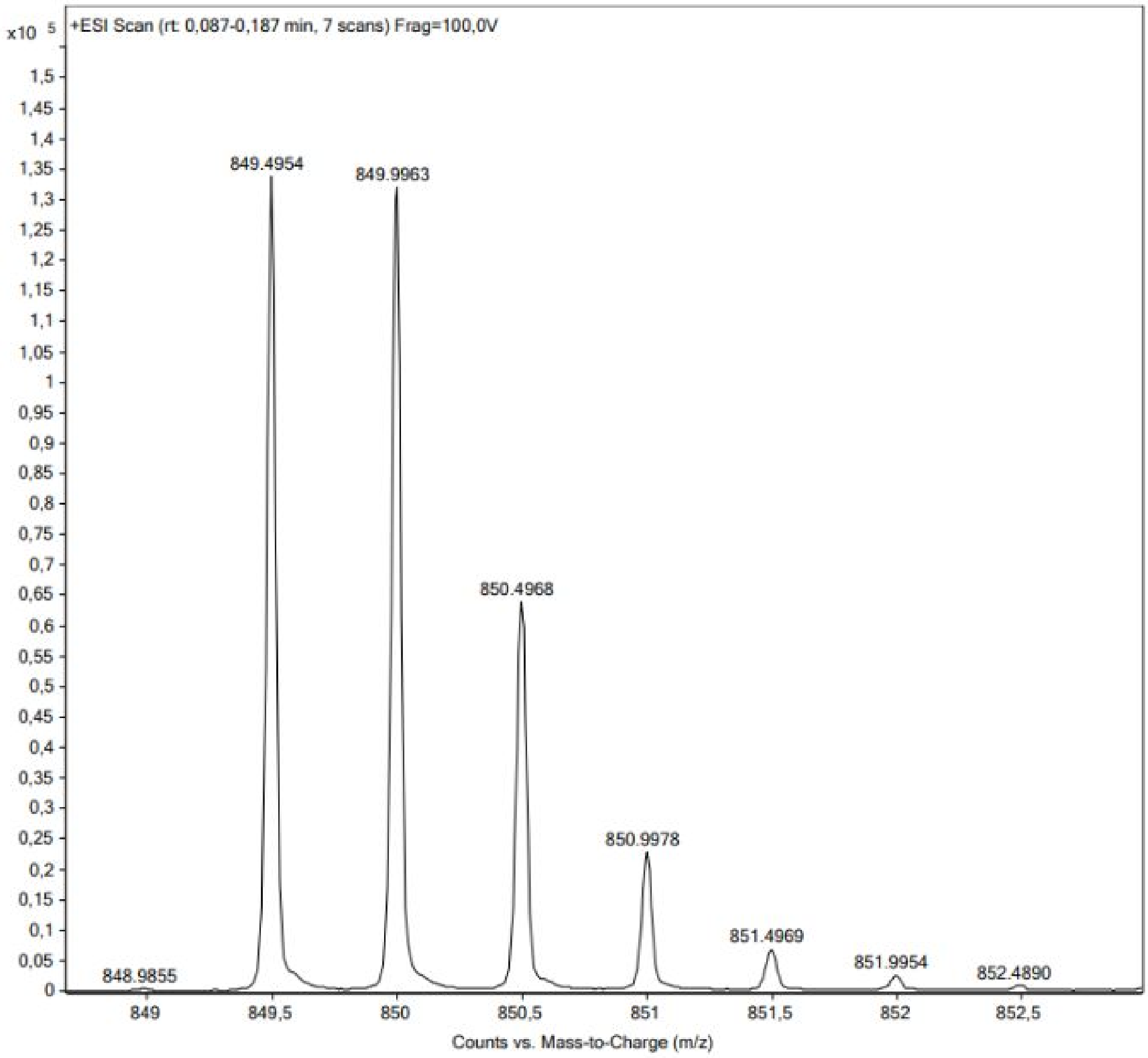
x10 5
+ESI Scan (rt: 0,087-0,187 min, 7 scans) Frag=100,0V
849.4954
849.9963
850.4968
850.9978
851.4969
851.9954
852.4890
848.9855
Counts vs. Mass-to-Charge (m/z)

CV5

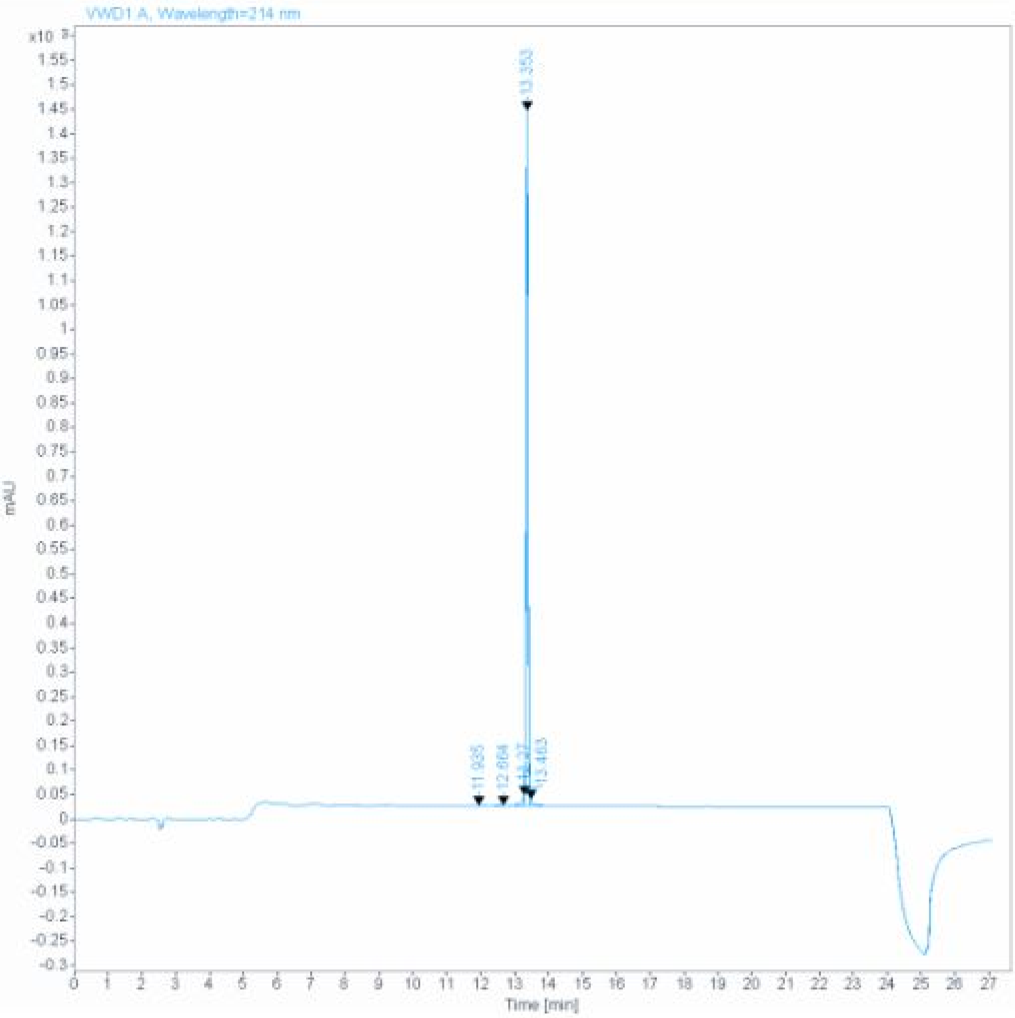

Signal: VWD1 A, Wavelength=214 nm

RT [min]	Type	Width [min]	Area	Height	Area%	Name
11.935	MM	0.0659	6.1483	1.5552	0.1065	
12.664	MM	0.1434	10.8226	1.2579	0.1874	
13.270	MF	0.0850	112.4281	22.0552	1.9468	
13.353	MF	0.0651	5568.5112	1425.5956	96.4229	
13.463	FM	0.0744	77.1844	14.2262	1.3365	
		Sum	5775.0946			

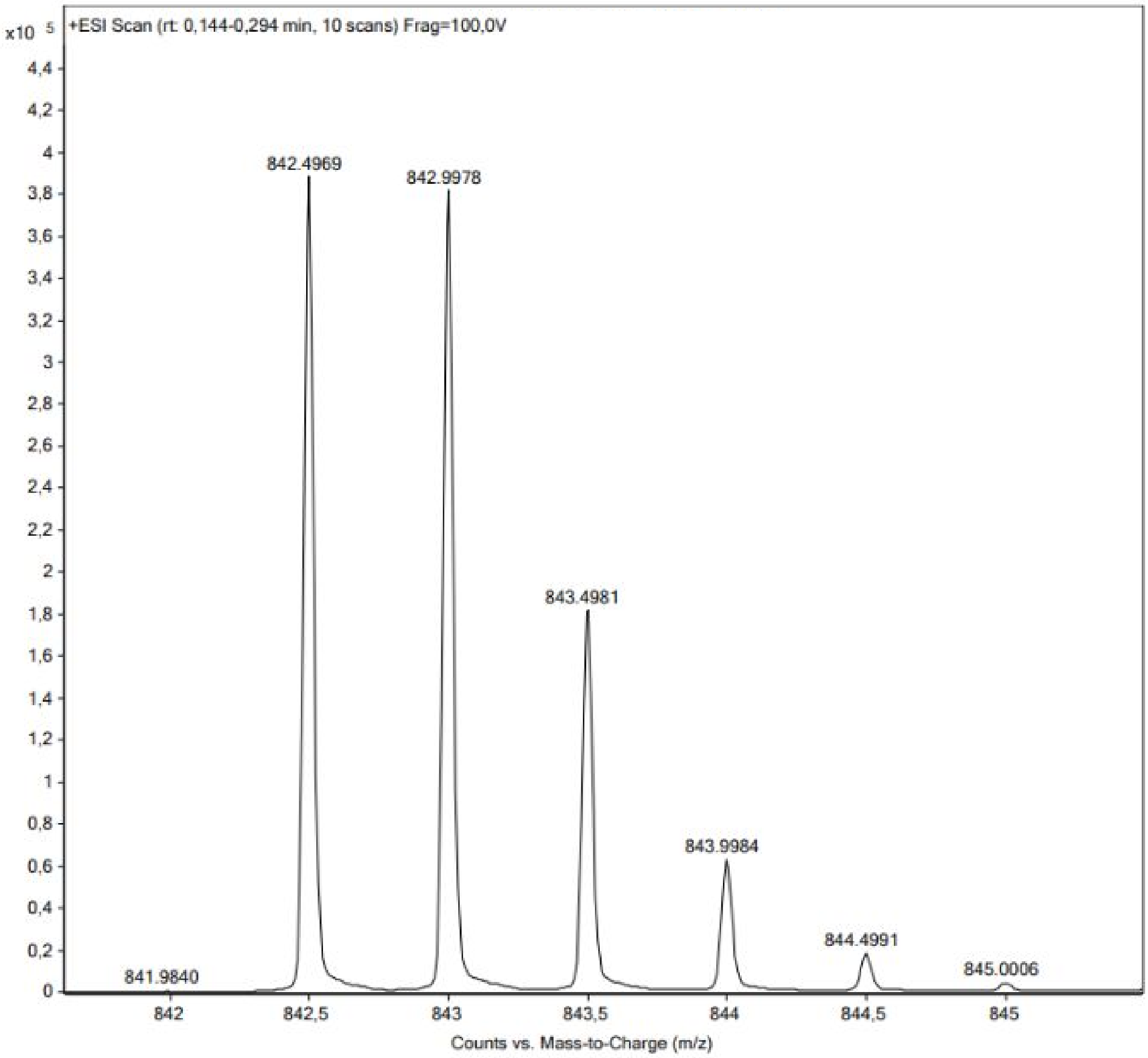

x10 5
+ESI Scan (rt: 0,144-0,294 min, 10 scans) Frag=100,0V
842.4969
842.9978
843.4981
843.9984
844.4991
845.0006
841.9840
Counts vs. Mass-to-Charge (m/z)

CV6

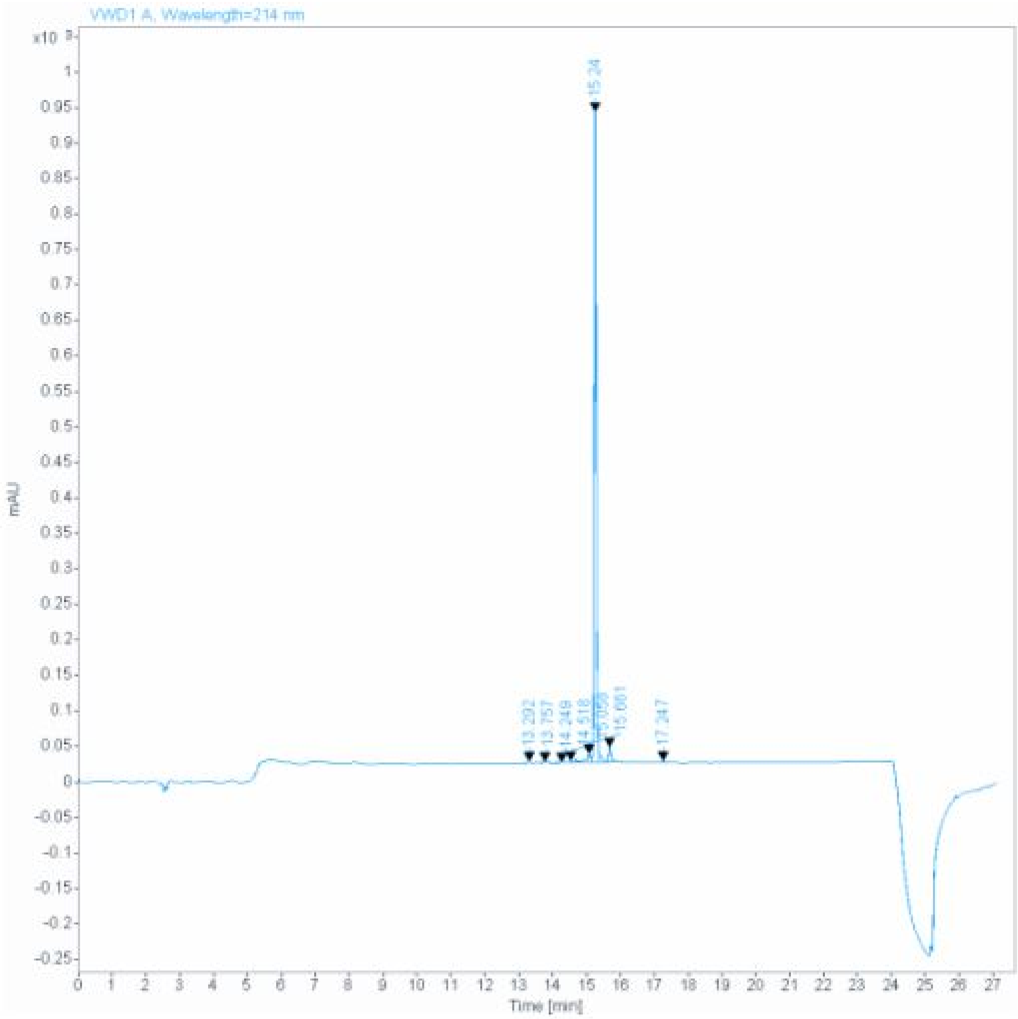

Signal: VWD1 A, Wavelength=214 nm

RT [min]	Type	Width [min]	Area	Height	Area%	Name
13.292	MM	0.0753	11.0685	2.4488	0.2716	
13.757	MM	0.1532	16.6083	1.8071	0.4075	
14.249	MM	0.1872	9.8143	0.8740	0.2408	
14.518	MM	0.0841	14.4001	2.8541	0.3533	
15.058	MF	0.1766	131.4162	12.4040	3.2242	
15.240	MF	0.0672	3706.7092	919.4766	90.9405	
15.661	FM	0.1394	178.1678	21.3061	4.3712	
17.247	MM	0.0566	7.7873	2.2915	0.1911	
		Sum	4075.9718			

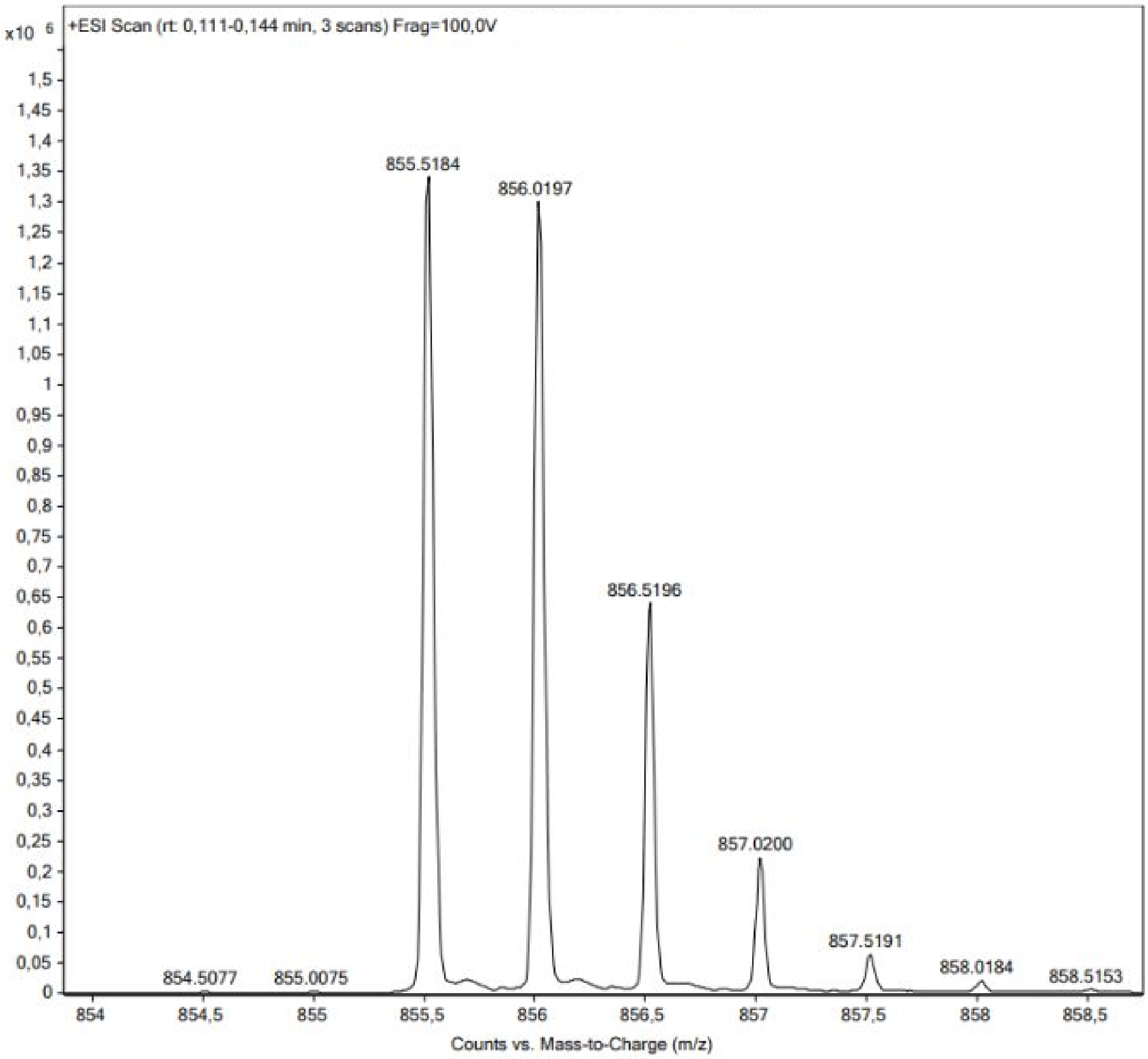
x10 6
+ESI Scan (rt: 0,111-0,144 min, 3 scans) Frag=100,0V
855.5184
856.0197
856.5196
857.0200
857.5191
858.0184
858.5153
854.5077
855.0075
Counts vs. Mass-to-Charge (m/z)

CV8

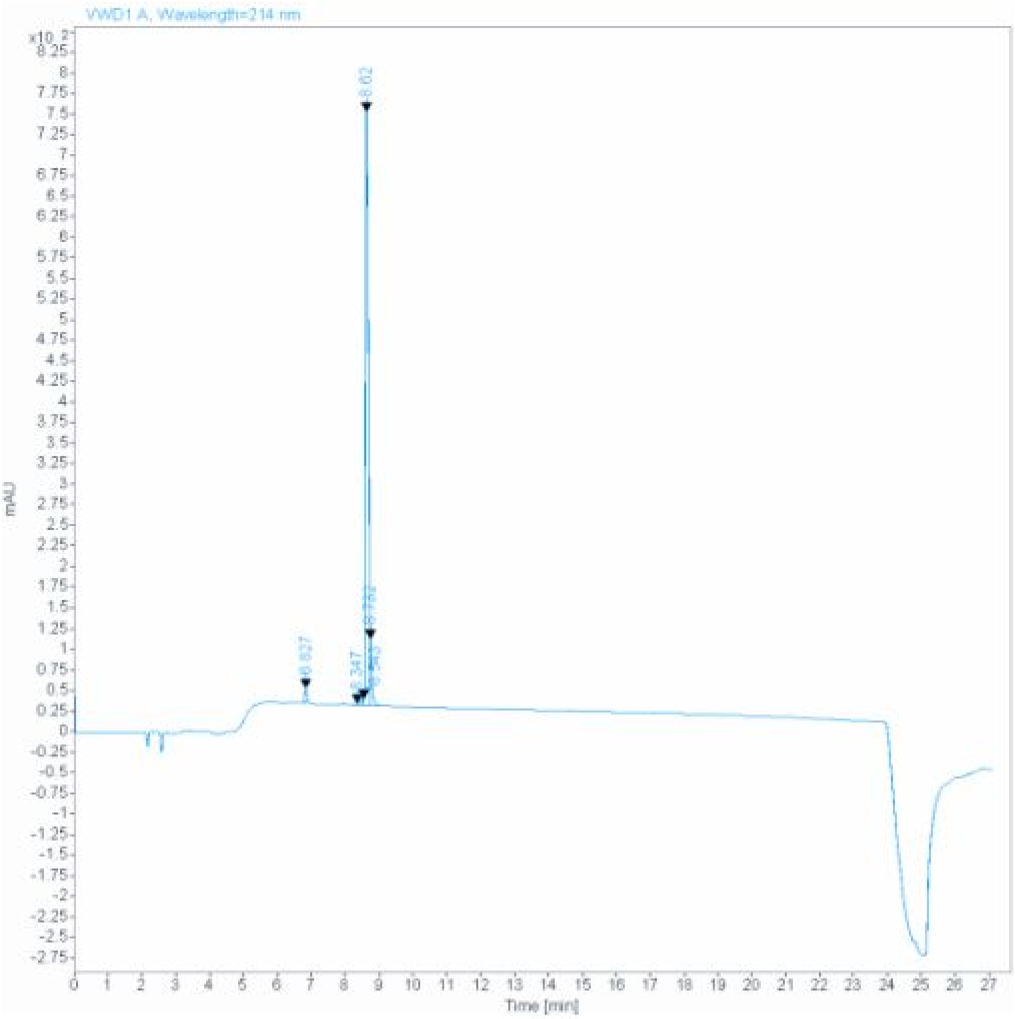

Signal: VWD1 A, Wavelength=214 nm

RT [min]	Type	Width [min]	Area	Height	Area%	Name
6.827	MM	0.0721	76.6317	17.7135	1.7171	
8.347	MM	0.0426	5.7685	2.2583	0.1293	
8.543	MF	0.0908	44.9164	8.2427	1.0064	
8.620	FM	0.0949	4114.7559	722.8049	92.1978	
8.732	FM	0.0455	220.8925	80.8771	4.9495	
		Sum	4462.9649			

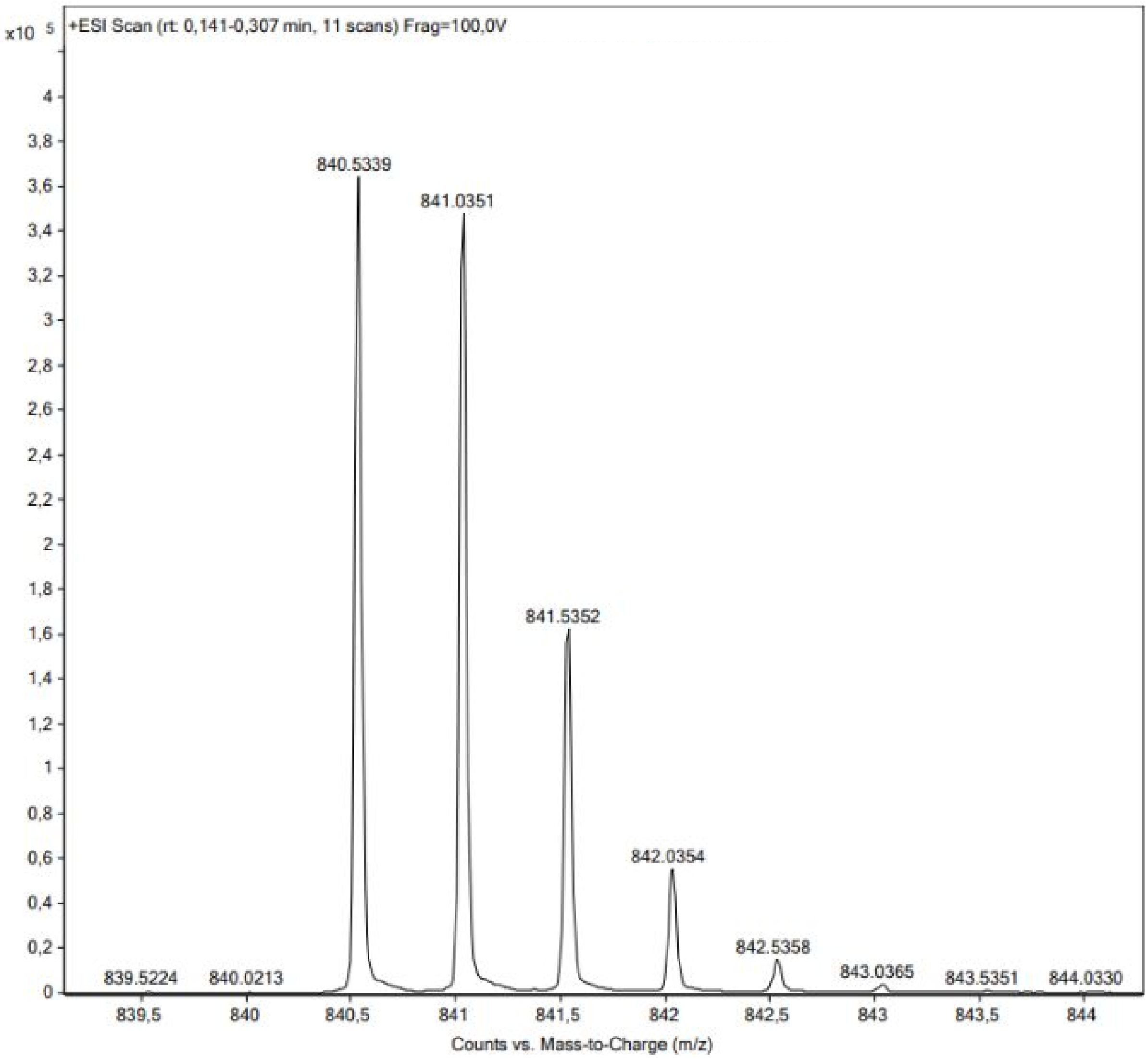
+ESI Scan (rt: 0,141-0,307 min, 11 scans) Frag=100,0V
x10 5
840.5339
841.0351
841.5352
842.0354
842.5358
839.5224
840.0213
843.0365
843.5351
844.0330
Counts vs. Mass-to-Charge (m/z)

CV9

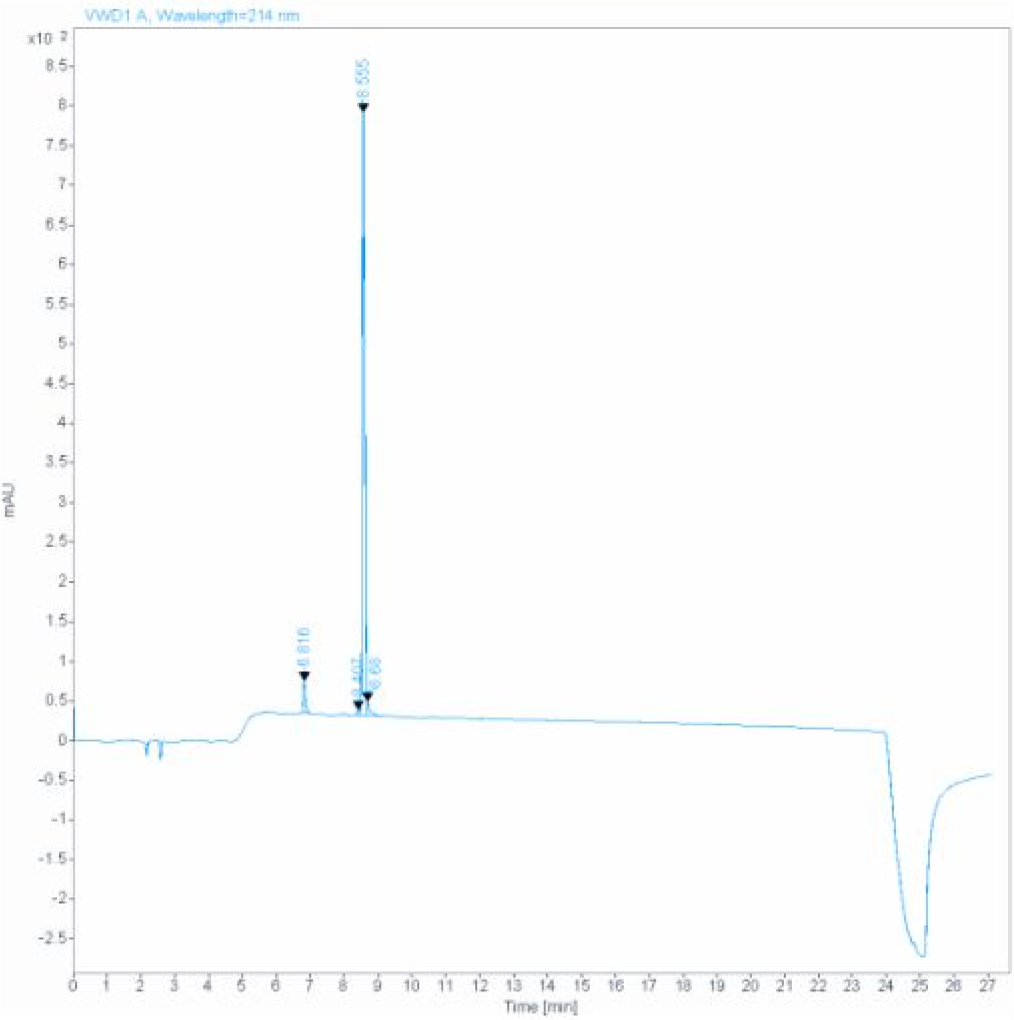

Signal: VWD1 A, Wavelength=214 nm

RT [min]	Type	Width [min]	Area	Height	Area%	Name
6.816	MM	0.0806	196.8454	40.7149	4.6123	
8.407	MF	0.0977	39.6664	6.7640	0.9294	
8.555	MF	0.0864	3946.1309	760.8558	92.4627	
8.680	FM	0.0809	85.1662	17.5373	1.9955	
		Sum	4267.8089			

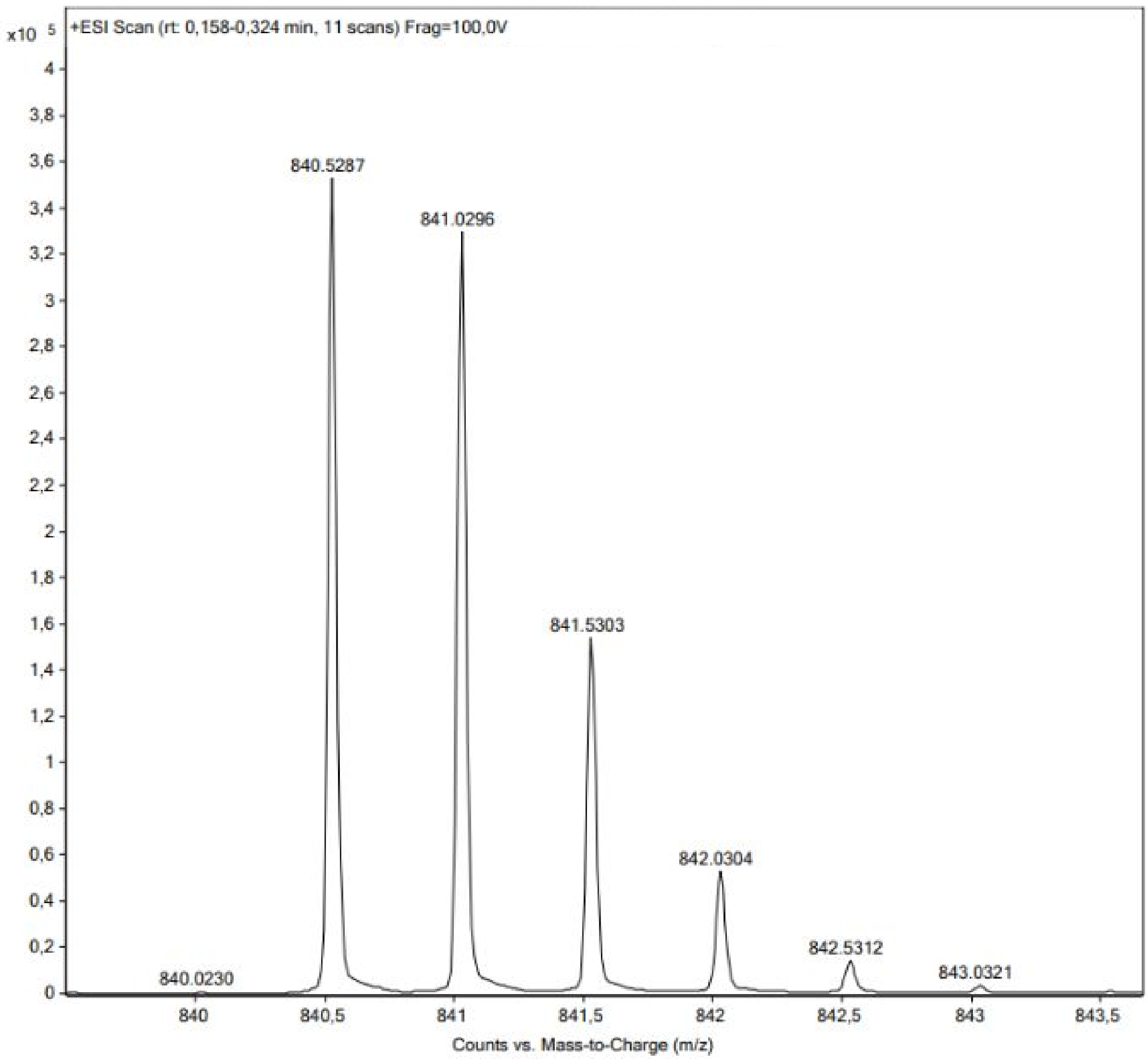
x10 5
+ESI Scan (rt: 0,158-0,324 min, 11 scans) Frag=100,0V
840.5287
841.0296
841.5303
842.0304
842.5312
843.0321
840.0230
4
3,8
3,6
3,4
3,2
3
2,8
2,6
2,4
2,2
2
1,8
1,6
1,4
1,2
1
0,8
0,6
0,4
0,2
0
840
840,5
841
841,5
842
842,5
843
843,5
Counts vs. Mass-to-Charge (m/z)

CV10

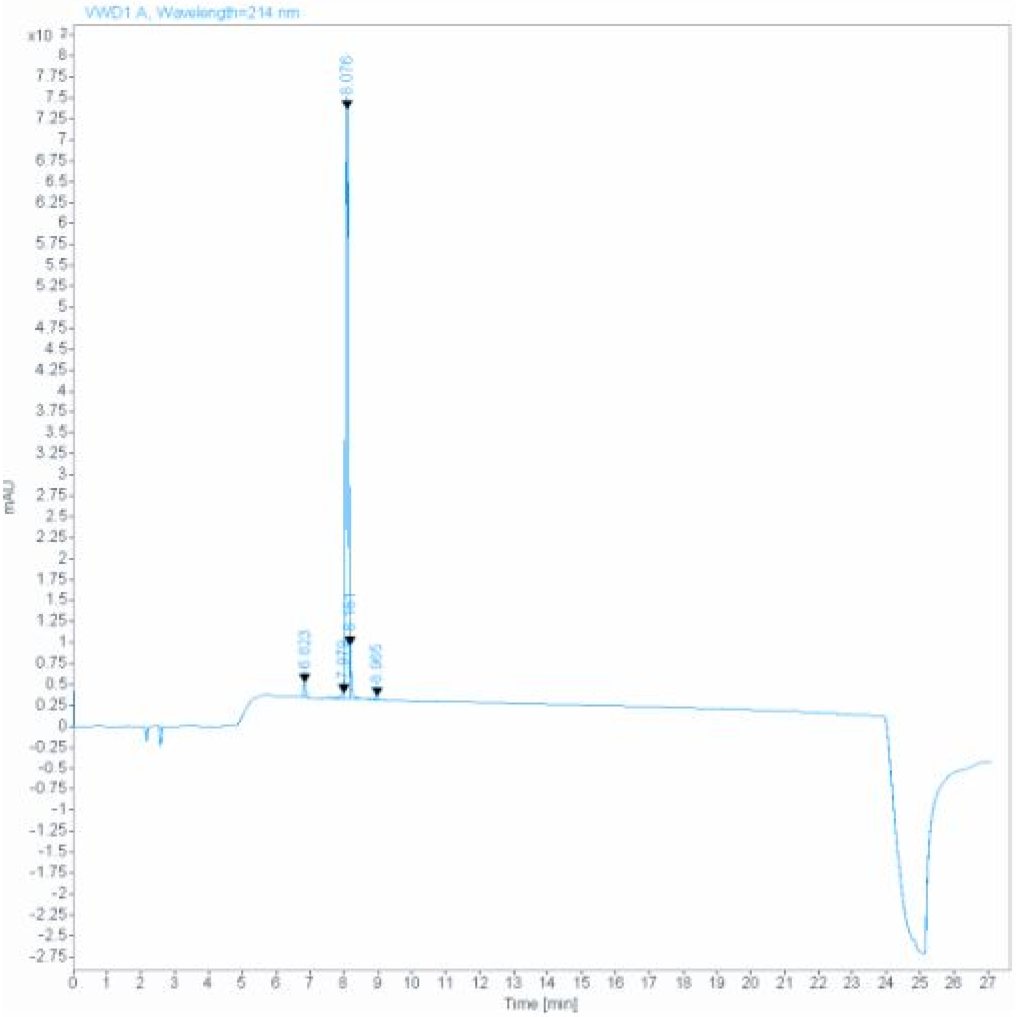

Signal: VWD1 A, Wavelength=214 nm

RT [min]	Type	Width [min]	Area	Height	Area%	Name
6.823	MM	0.0814	84.0216	17.2011	1.9034	
7.979	MF	0.1870	75.3474	6.7163	1.7069	
8.076	MF	0.0959	4053.1360	704.0845	91.8207	
8.161	FM	0.0478	177.8179	61.9802	4.0283	
8.965	MM	0.0939	23.8626	4.2338	0.5406	
		Sum	4414.1856			

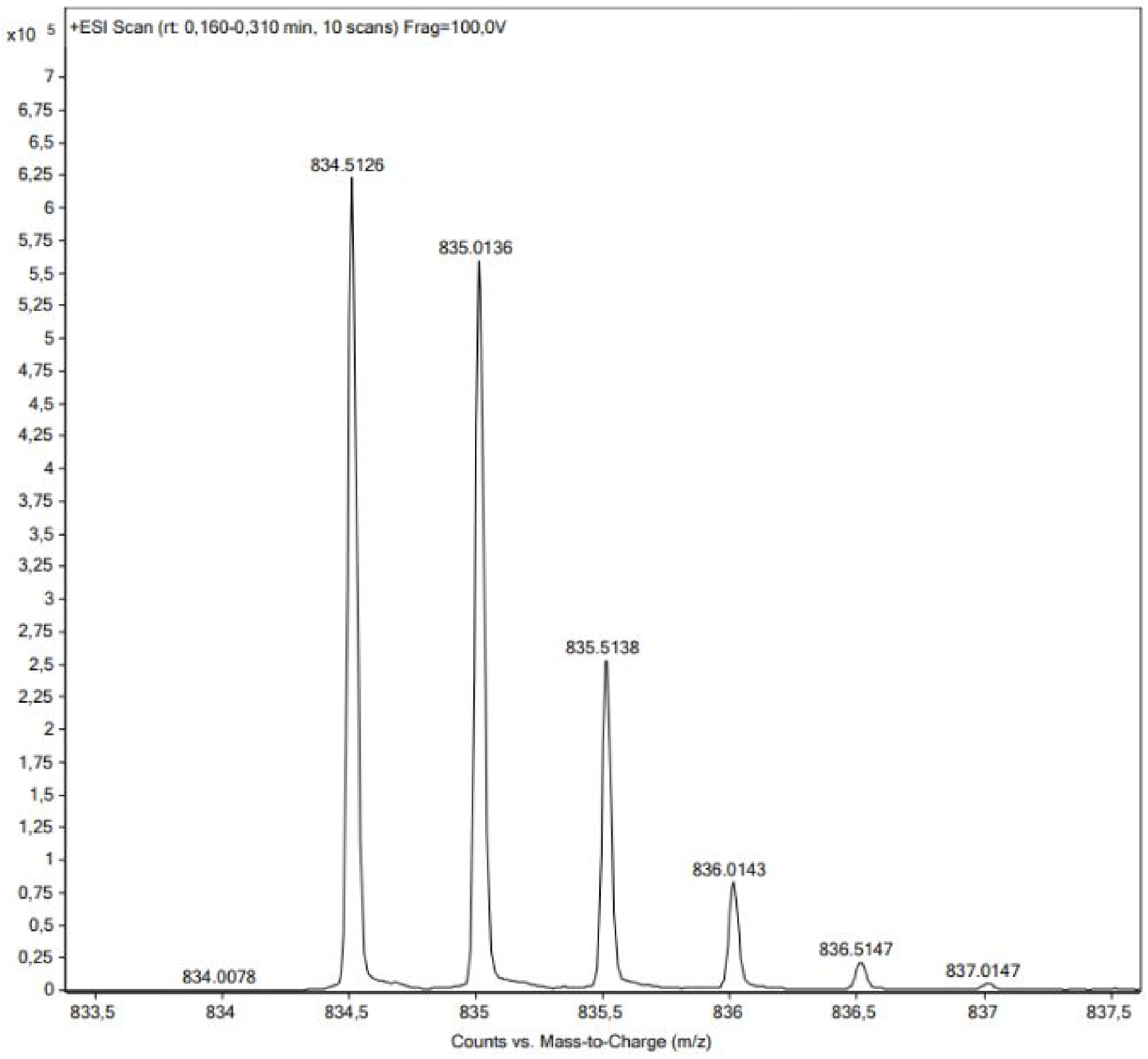
x10 5
+ESI Scan (rt: 0,160-0,310 min, 10 scans) Frag=100,0V
834.5126
835.0136
835.5138
836.0143
836.5147
837.0147
834.0078
Counts vs. Mass-to-Charge (m/z)

CV11

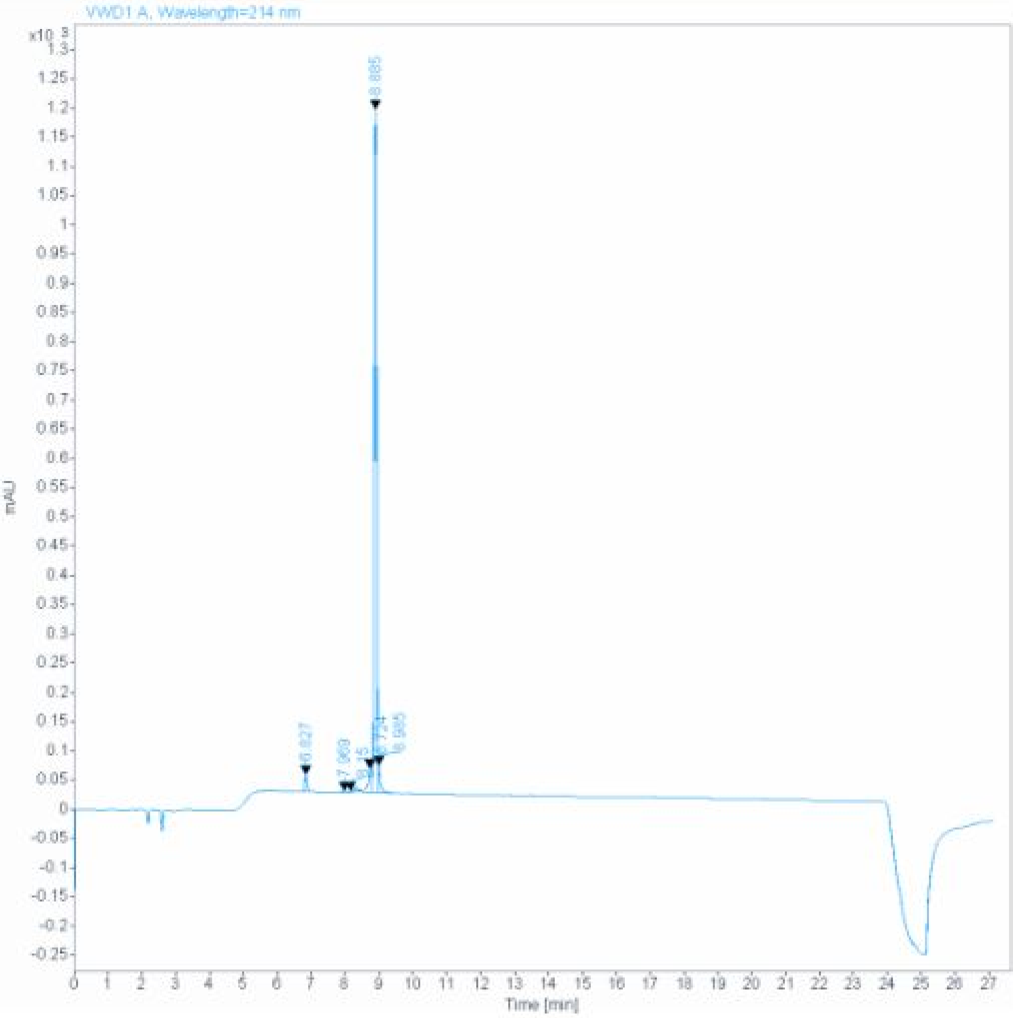

Signal: VWD1 A, Wavelength=214 nm

RT [min]	Type	Width [min]	Area	Height	Area%	Name
6.827	MM	0.0799	138.1575	28.8090	1.9515	
7.969	MM	0.0998	13.6798	2.2840	0.1932	
8.150	MM	0.0470	6.0030	2.1284	0.0848	
8.724	MF	0.1555	383.0525	41.0560	5.4106	
8.885	MF	0.0907	6366.2998	1170.3442	89.9243	
8.985	FM	0.0612	172.4248	46.9433	2.4355	
		Sum	7079.6173			

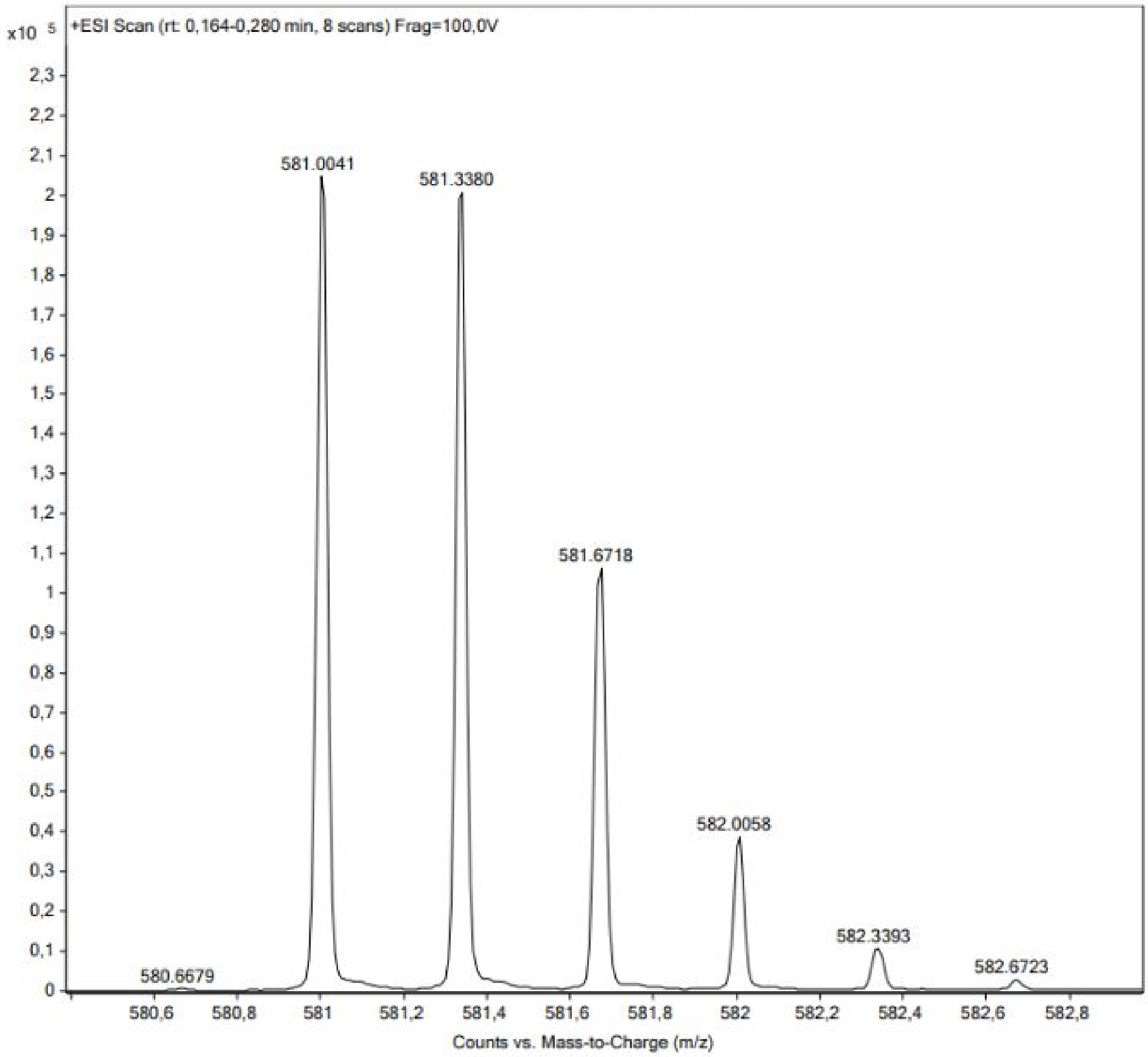
x10 5
+ESI Scan (rt: 0,164-0,280 min, 8 scans) Frag=100,0V
2,3
2,2
2,1
2
1,9
1,8
1,7
1,6
1,5
1,4
1,3
1,2
1,1
1
0,9
0,8
0,7
0,6
0,5
0,4
0,3
0,2
0,1
0
581.0041
581.3380
581.6718
582.0058
582.3393
582.6723
580.6679
580,6
580,8
581
581,2
581,4
581,6
581,8
582
582,2
582,4
582,6
582,8
Counts vs. Mass-to-Charge (m/z)

CV12

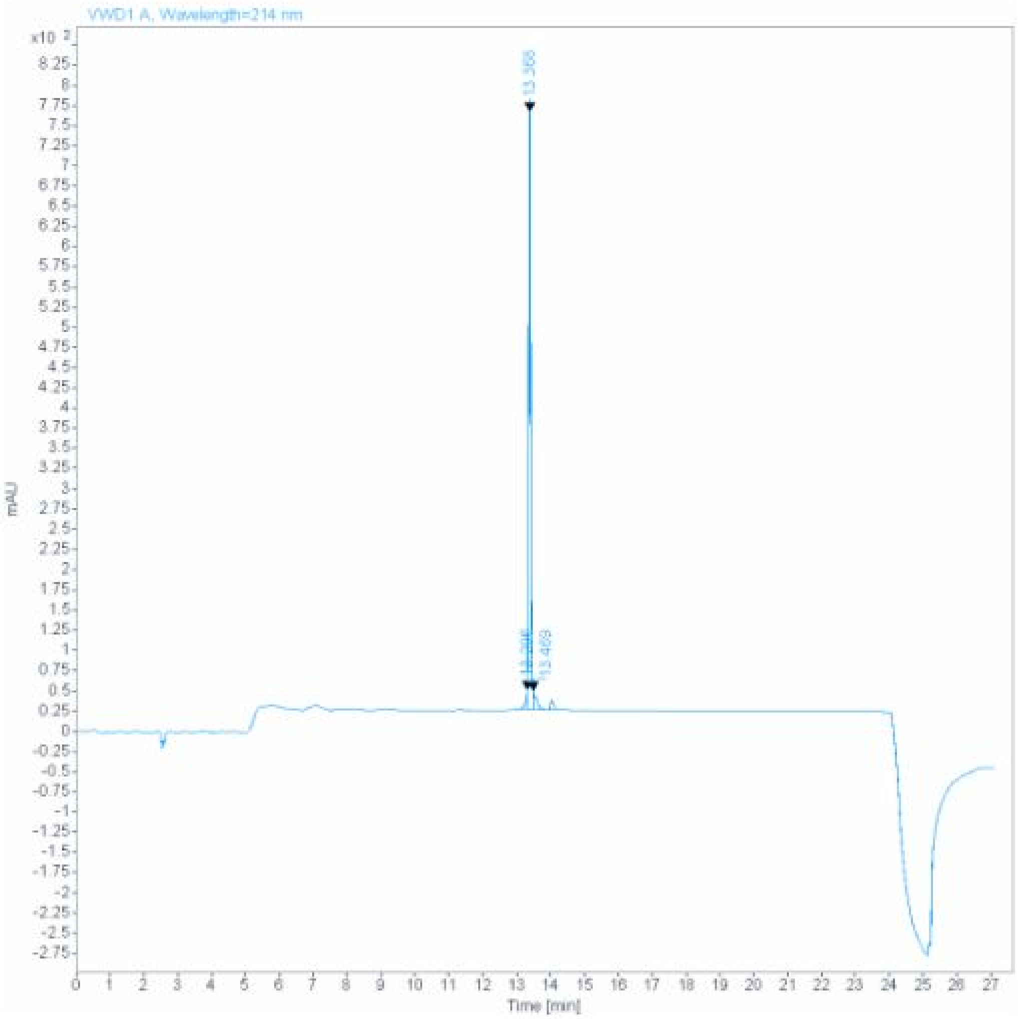

Signal: VWD1 A, Wavelength=214 nm

RT [min]	Type	Width [min]	Area	Height	Area%	Name
13.296	MF	0.0588	87.3972	24.7894	2.6464	
13.368	MF	0.0664	2959.8569	742.7513	89.6241	
13.469	FM	0.1821	255.2679	23.3609	7.7295	
		Sum	3302.5220			

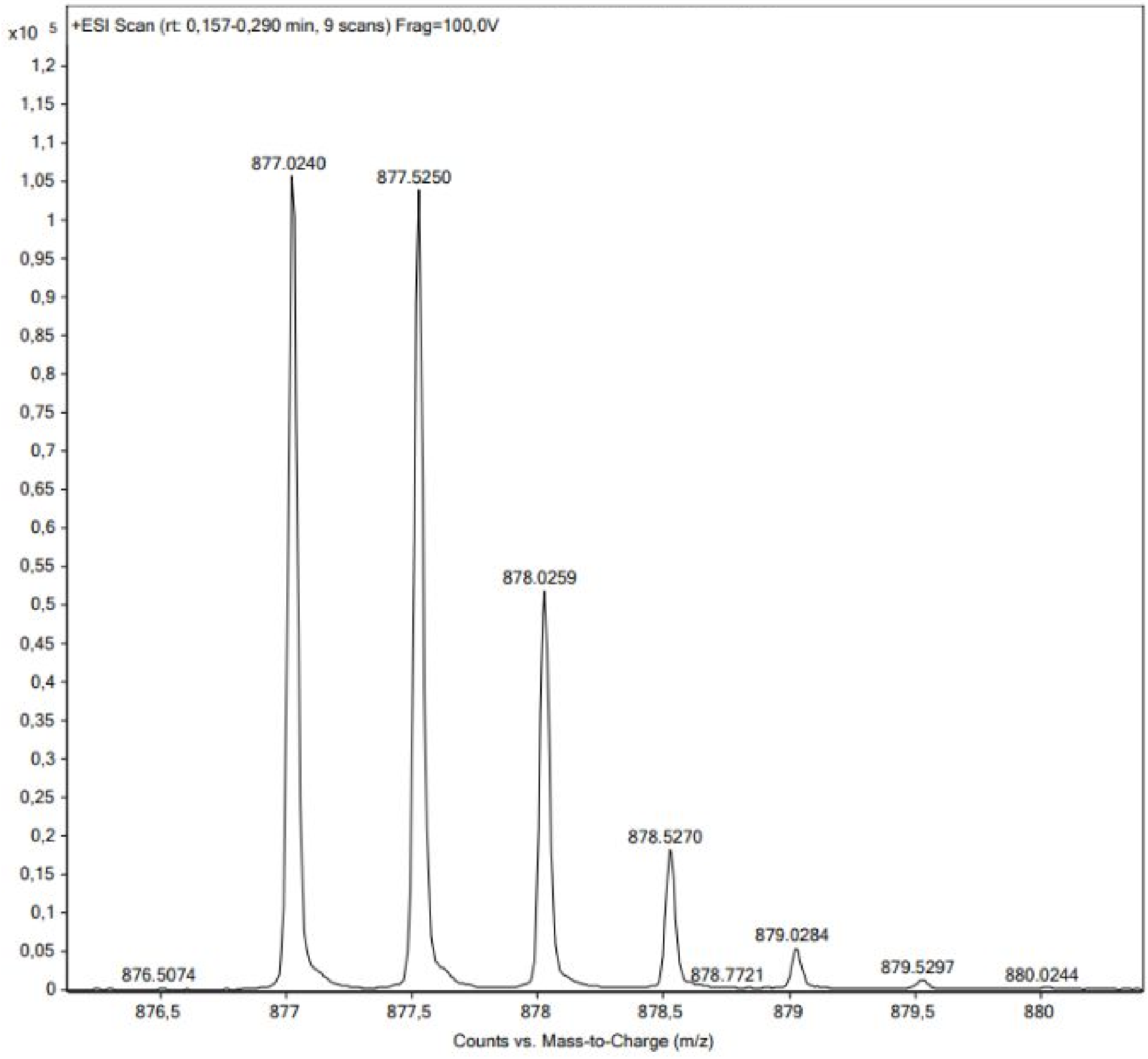

x10 5
+ESI Scan (rt: 0,157-0,290 min, 9 scans) Frag=100,0V
877.0240
877.5250
878.0259
878.5270
879.0284
876.5074
878.7721
879.5297
880.0244
Counts vs. Mass-to-Charge (m/z)

HV1

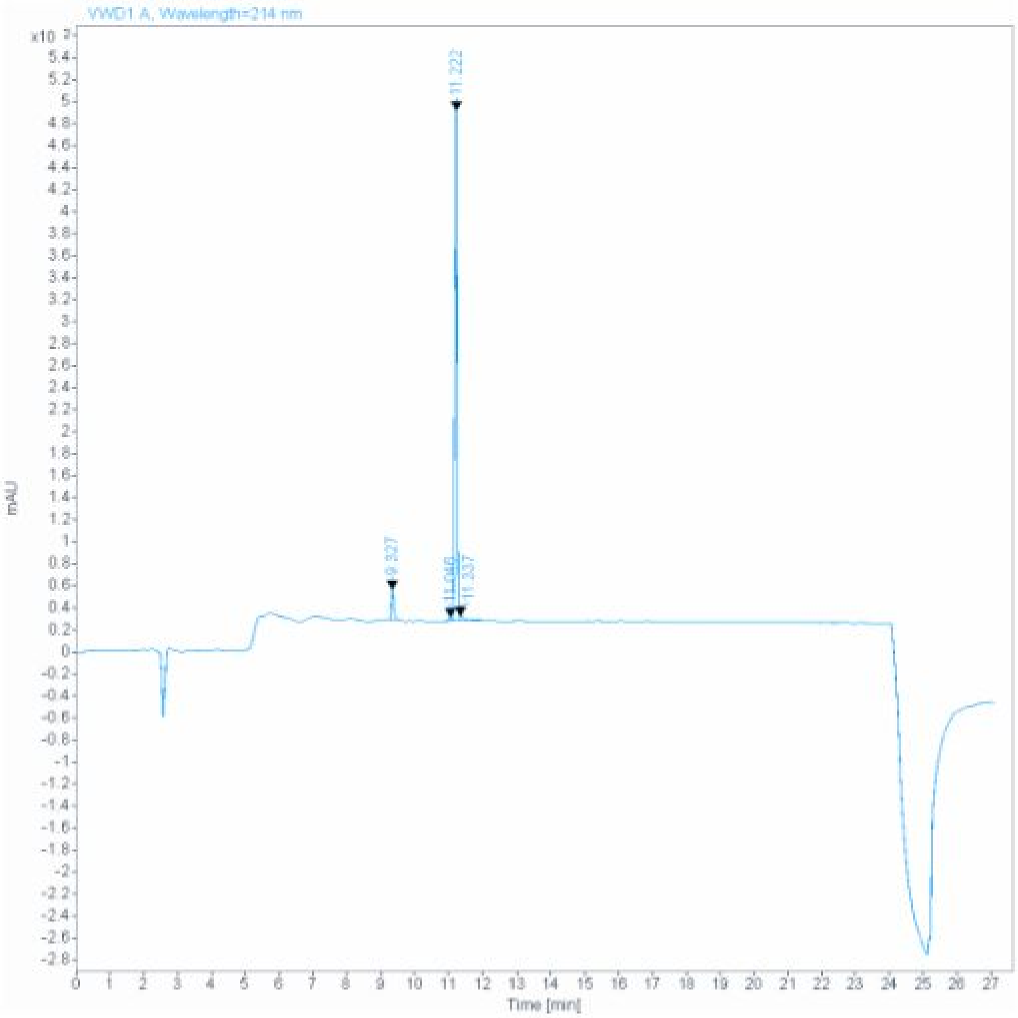

Signal: VWD1 A, Wavelength=214 nm

RT [min]	Type	Width [min]	Area	Height	Area%	Name
9.327	MM	0.0827	133.7636	26.9457	5.5527	
11.046	MF	0.1072	22.9554	3.5681	0.9529	
11.222	MF	0.0795	2213.5806	464.1086	91.8889	
11.337	FM	0.1448	38.6765	4.4504	1.6055	
		Sum	2408.9761			

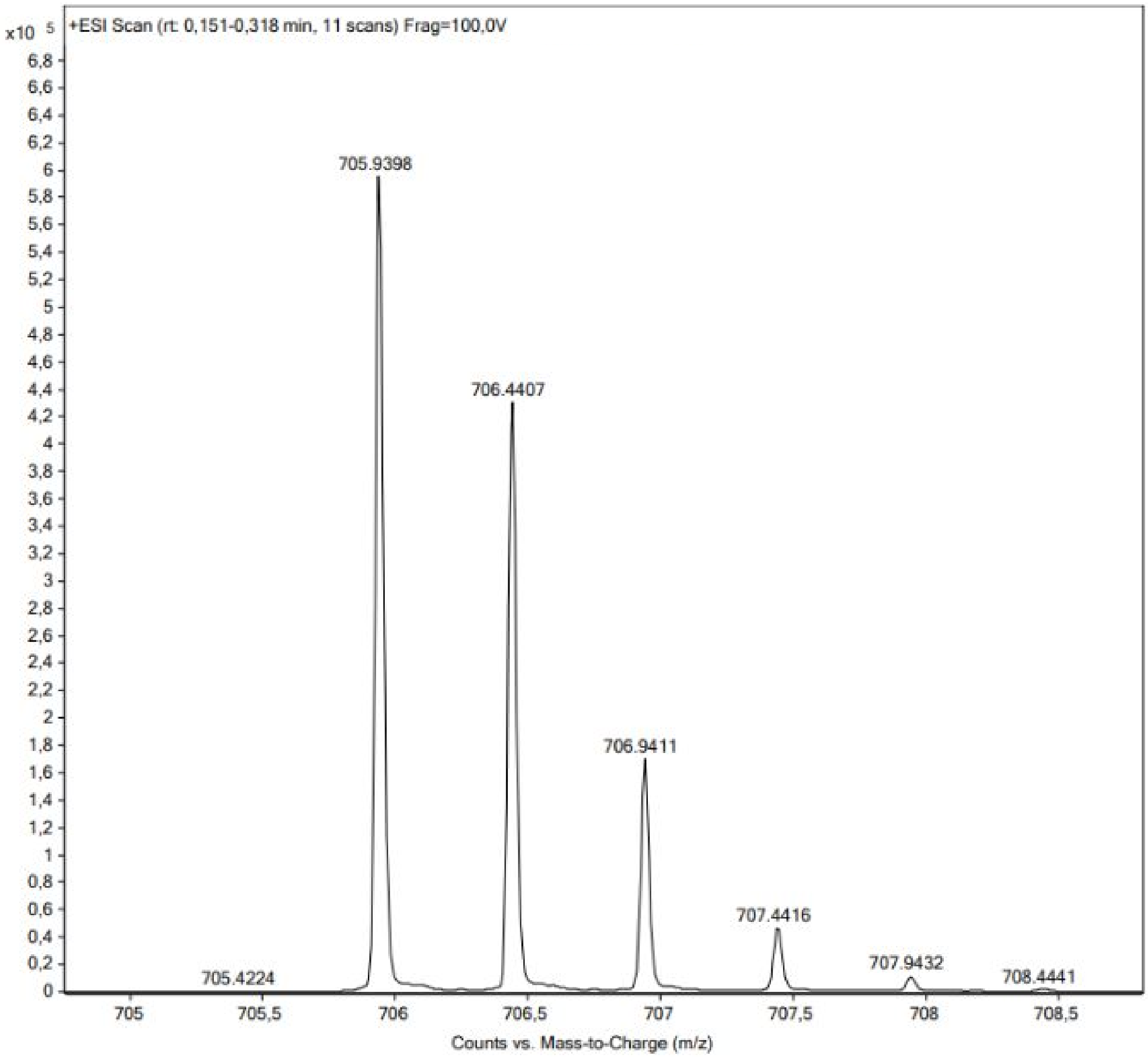

x10 5
+ESI Scan (rt: 0,151-0,318 min, 11 scans) Frag=100,0V
705.9398
706.4407
706.9411
707.4416
707.9432
708.4441
705.4224
Counts vs. Mass-to-Charge (m/z)

HV2

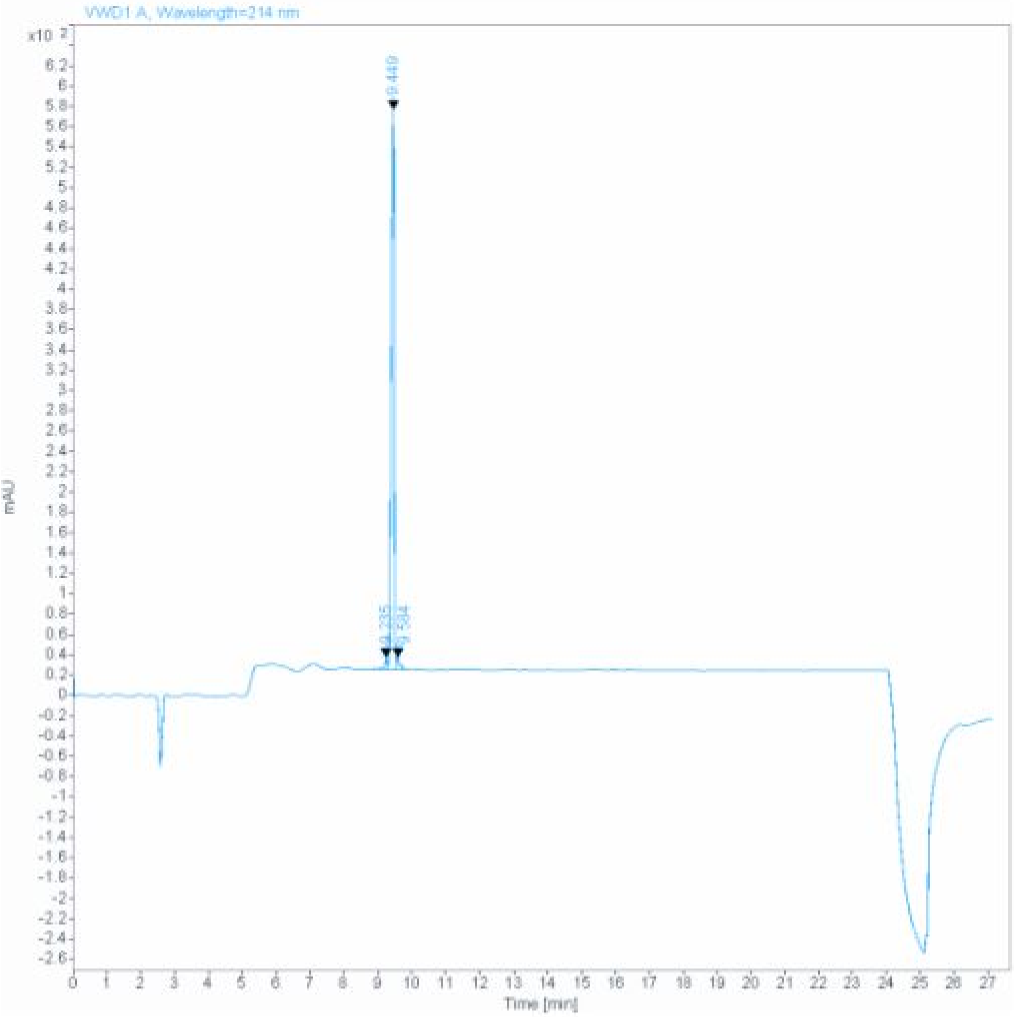

Signal: VWD1 A, Wavelength=214 nm

RT [min]	Type	Width [min]	Area	Height	Area%	Name
9.235	MF	0.0907	63.5510	11.6817	1.7423	
9.449	MF	0.1062	3517.5188	552.1094	96.4342	
9.584	FM	0.0937	66.5143	11.8262	1.8235	
		Sum	3647.5841			

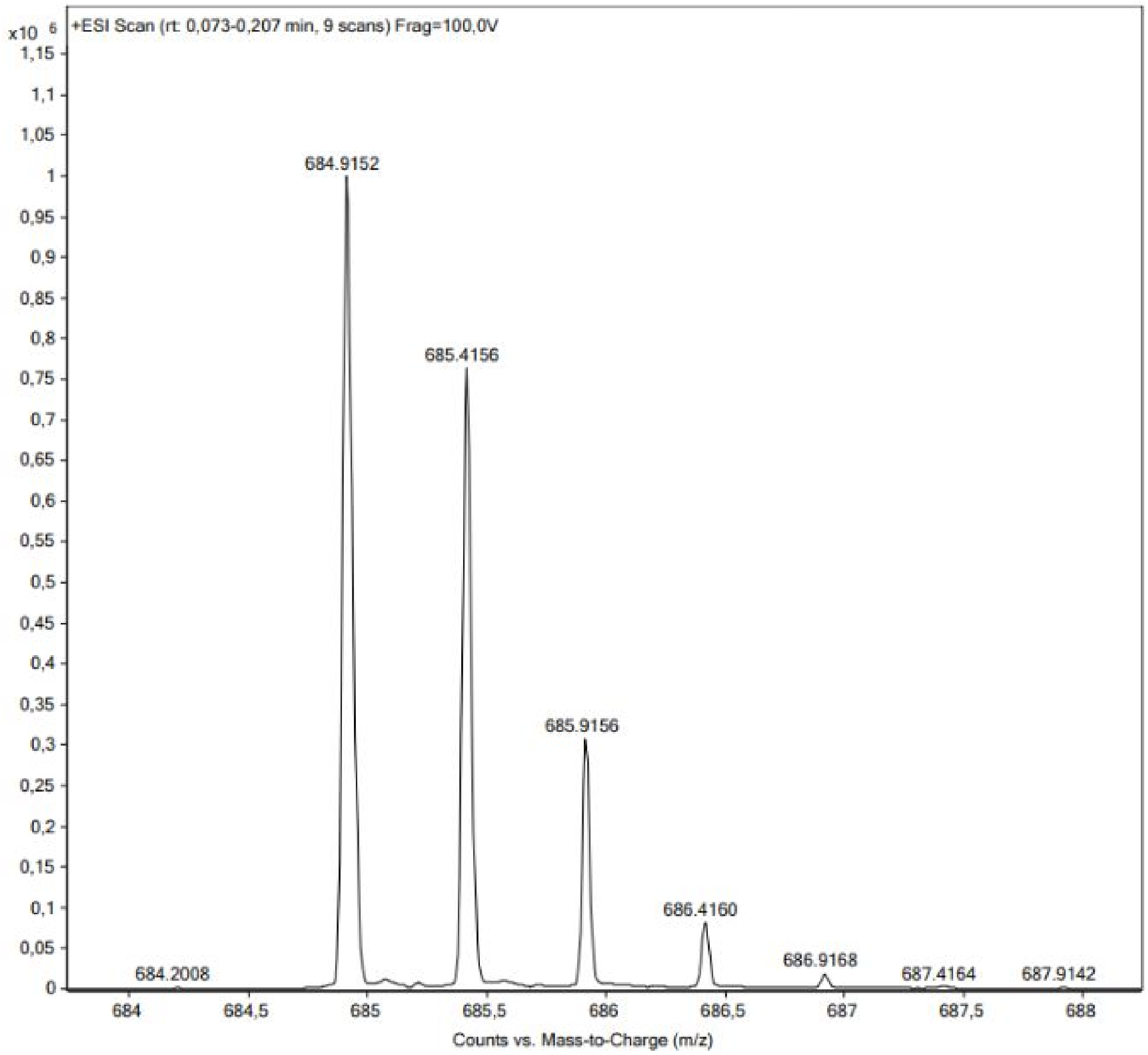
x10 6
+ESI Scan (rt: 0,073-0,207 min, 9 scans) Frag=100,0V
684.9152
685.4156
685.9156
686.4160
686.9168
684.2008
687.4164
687.9142
Counts vs. Mass-to-Charge (m/z)

HV3

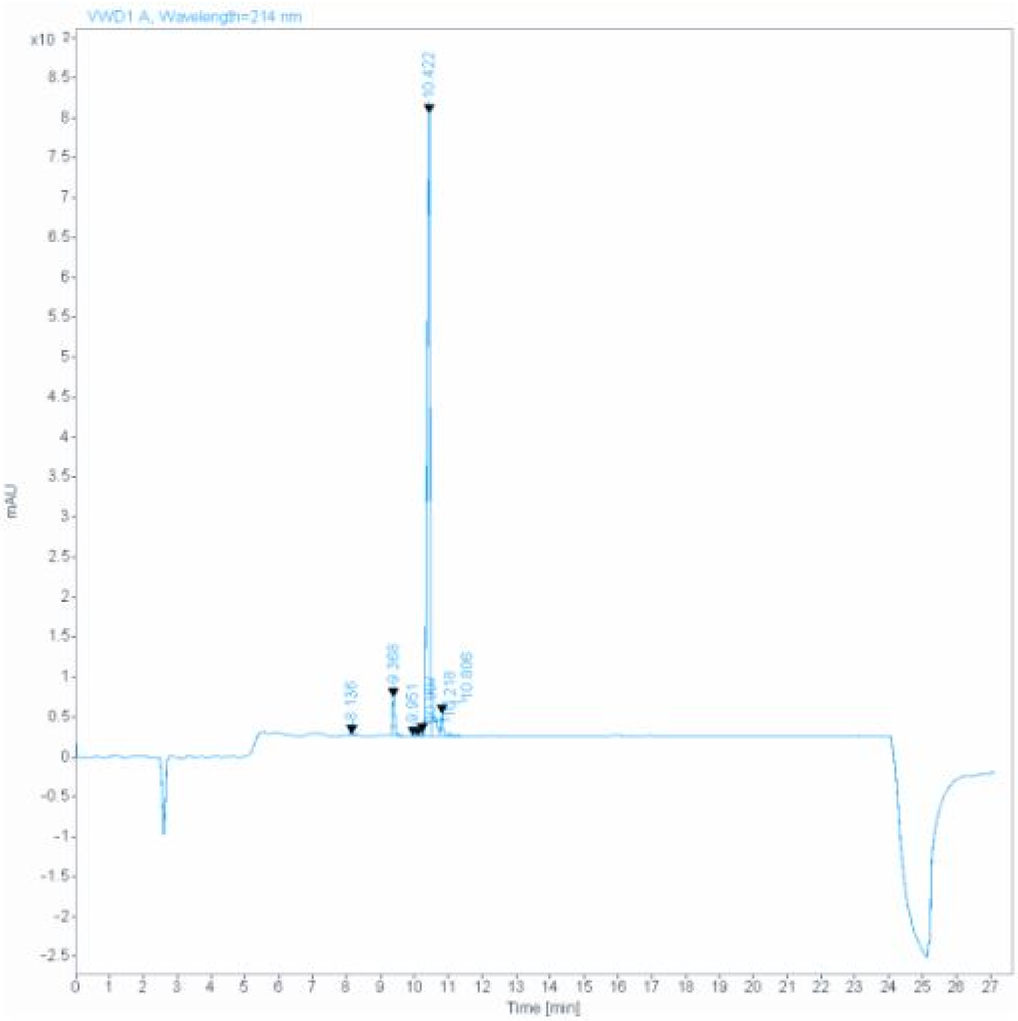

Signal: VWD1 A, Wavelength=214 nm

RT [min]	Type	Width [min]	Area	Height	Area%	Name
8.136	MM	0.0831	4.9446	0.9919	0.0905	
9.368	MM	0.0874	253.5662	48.3601	4.6430	
9.951	MM	0.1128	4.1039	0.6064	0.0751	
10.097	MM	0.0578	2.4116	0.6955	0.0442	
10.218	MF	0.0425	13.6431	5.3522	0.2498	
10.422	FM	0.1022	4779.1509	779.6428	87.5097	
10.806	FM	0.2349	403.4581	28.6269	7.3876	
		Sum	5461.2785			

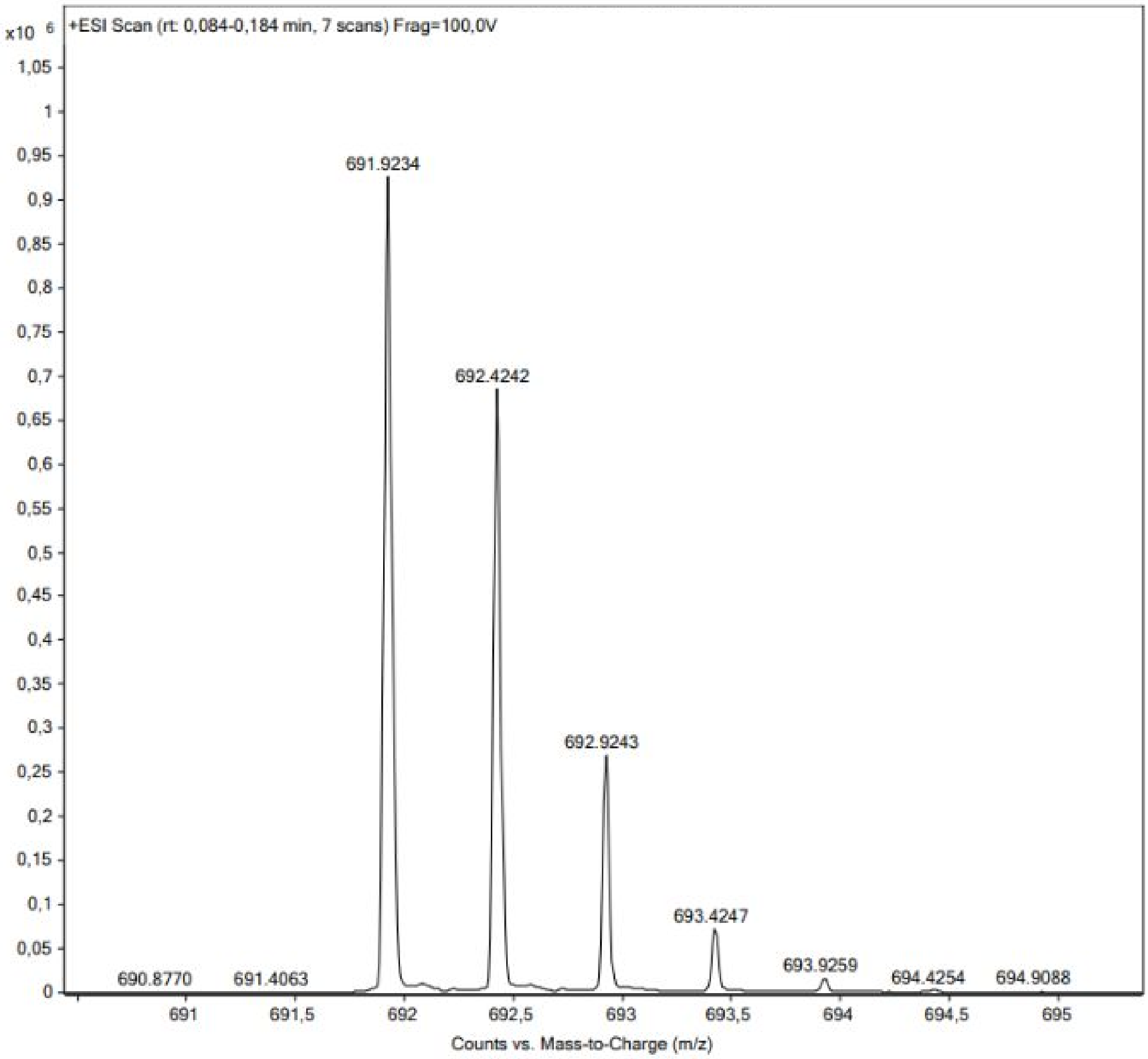
x10 6
+ESI Scan (rt: 0,084-0,184 min, 7 scans) Frag=100,0V
691.9234
692.4242
692.9243
693.4247
693.9259
690.8770
691.4063
694.4254
694.9088
Counts vs. Mass-to-Charge (m/z)

HV4

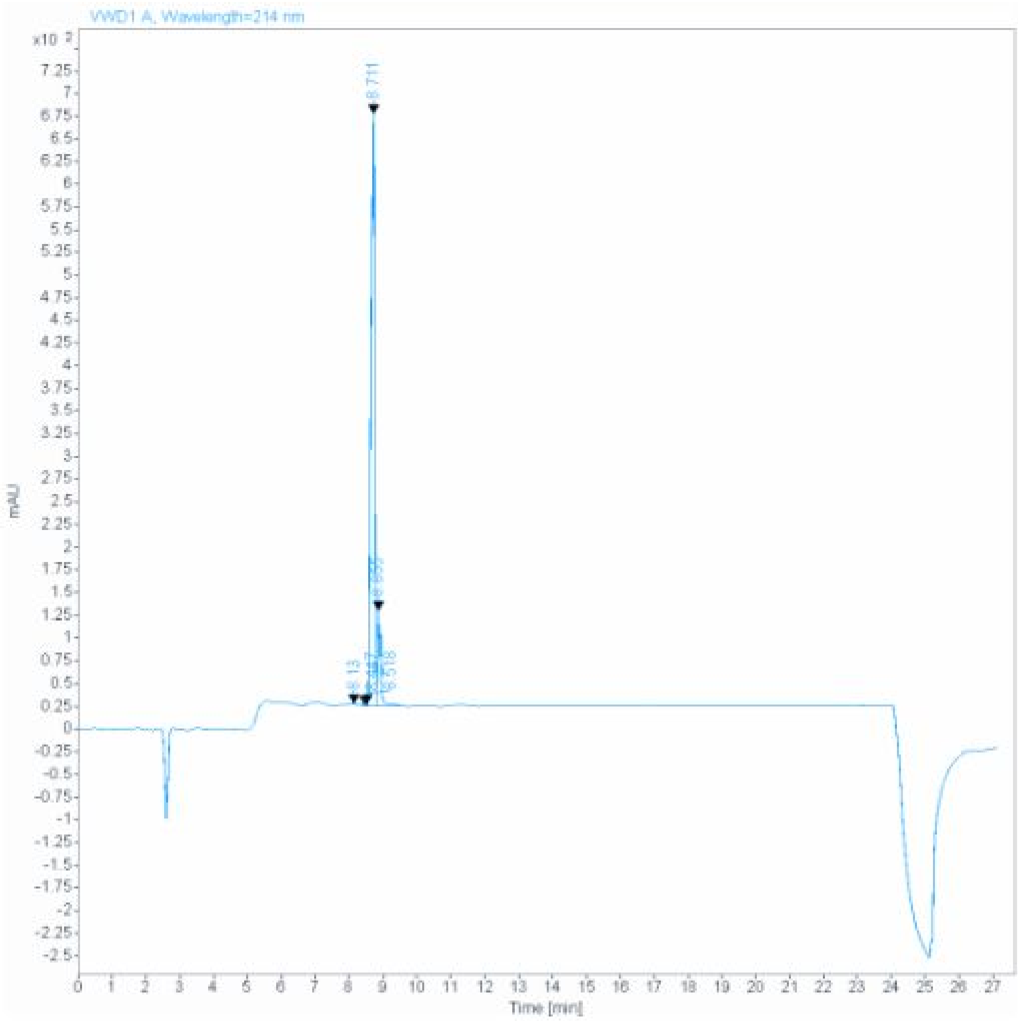

Signal: VWD1 A, Wavelength=214 nm

RT [min]	Type	Width [min]	Area	Height	Area%	Name
8.130	MM	0.0642	3.4402	0.8932	0.0623	
8.447	MM	0.0408	2.1918	0.8944	0.0397	
8.518	MF	0.0258	12.2277	7.8926	0.2214	
8.711	FM	0.1227	4809.2422	653.4194	87.0902	
8.855	FM	0.1103	695.0372	105.0469	12.5864	
		Sum	5522.1391			

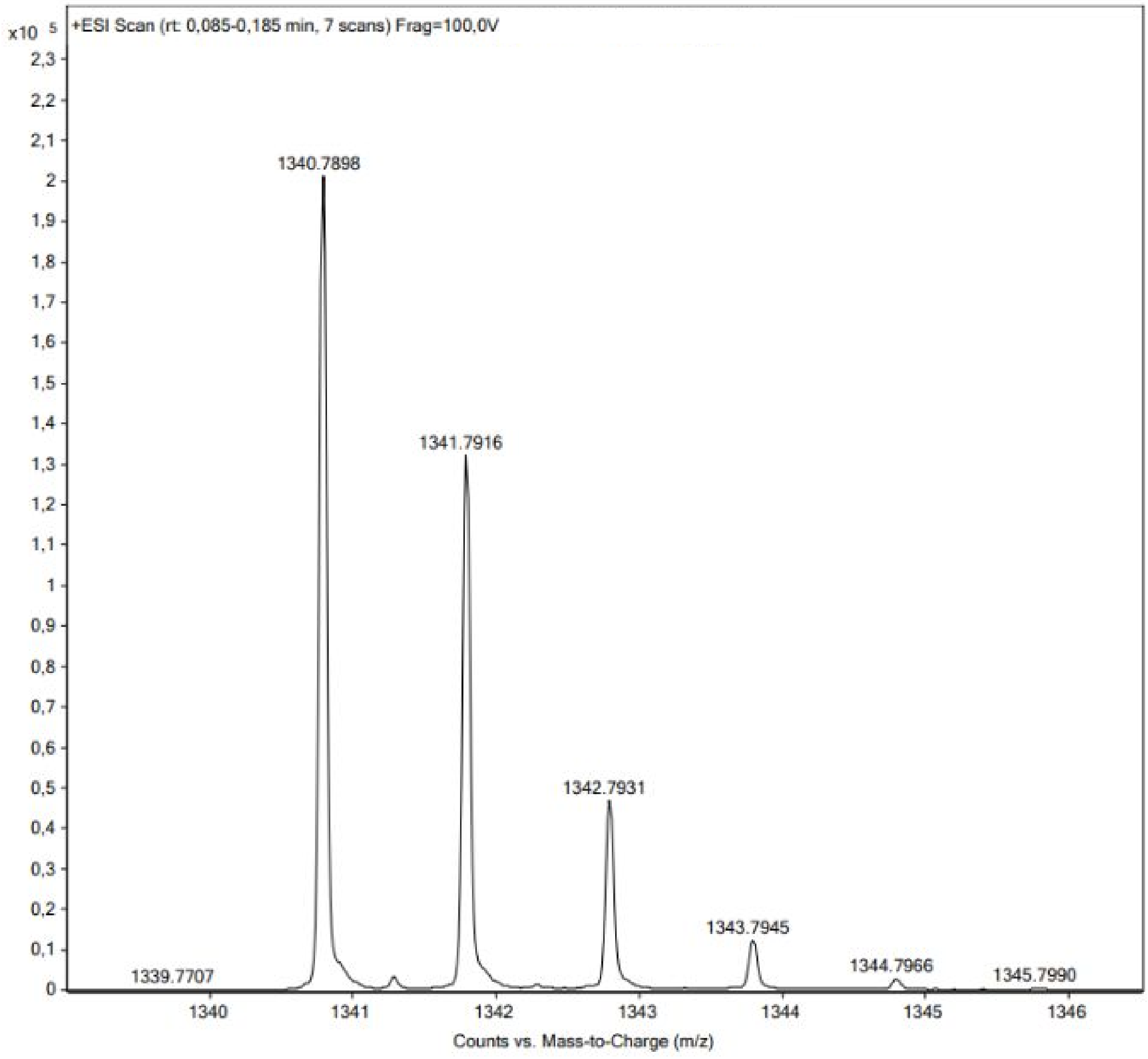
x10 5
+ESI Scan (rt: 0,085-0,185 min, 7 scans) Frag=100,0V
2,3
2,2
2,1
2
1,9
1,8
1,7
1,6
1,5
1,4
1,3
1,2
1,1
1
0,9
0,8
0,7
0,6
0,5
0,4
0,3
0,2
0,1
0
1340.7898
1341.7916
1342.7931
1343.7945
1344.7966
1345.7990
1339.7707
1340
1341
1342
1343
1344
1345
1346
Counts vs. Mass-to-Charge (m/z)

HV5

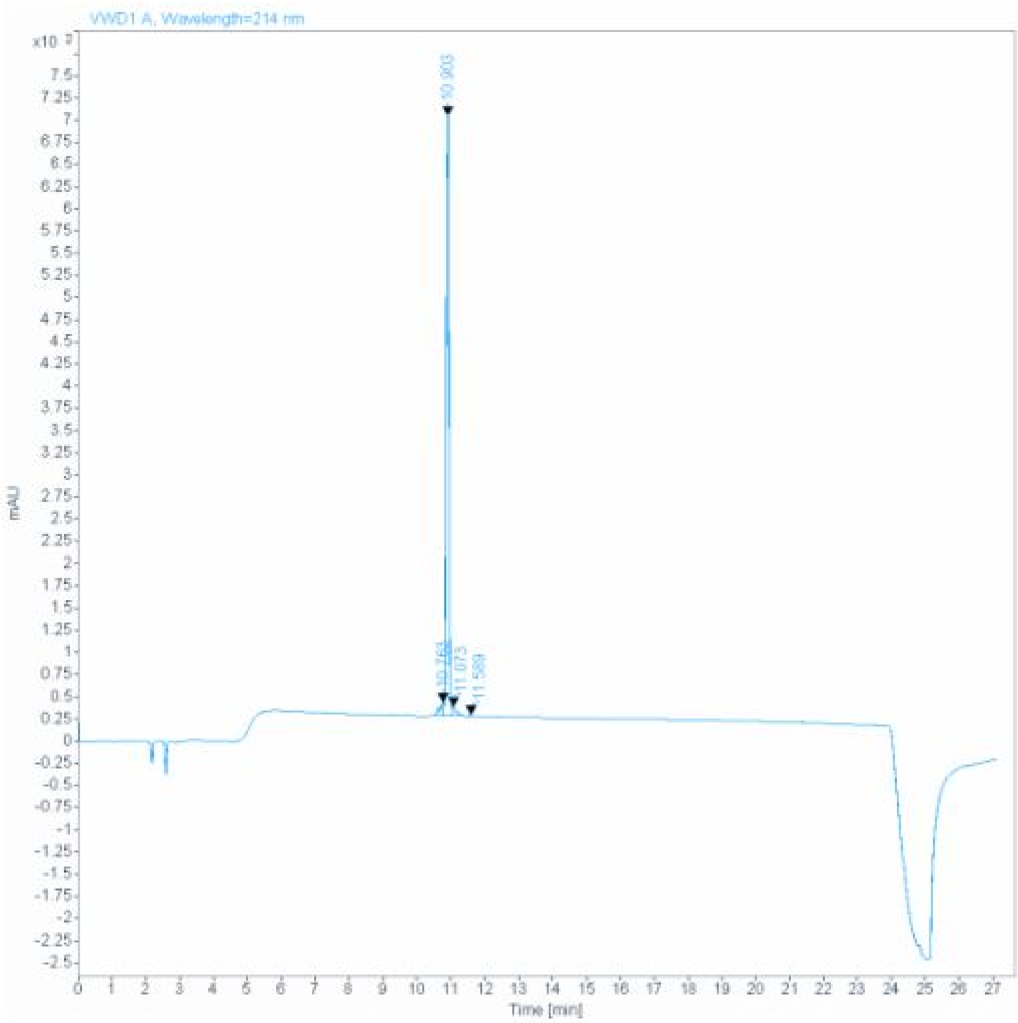

Signal: VWD1 A, Wavelength=214 nm

RT [min]	Type	Width [min]	Area	Height	Area%	Name
10.763	MF	0.1368	130.3530	15.8757	3.1032	
10.903	FM	0.0979	3977.2466	677.2116	94.6836	
11.073	FM	0.1352	89.3058	11.0075	2.1260	
11.589	MM	0.0380	3.6595	1.6071	0.0871	
		Sum	4200.5649			

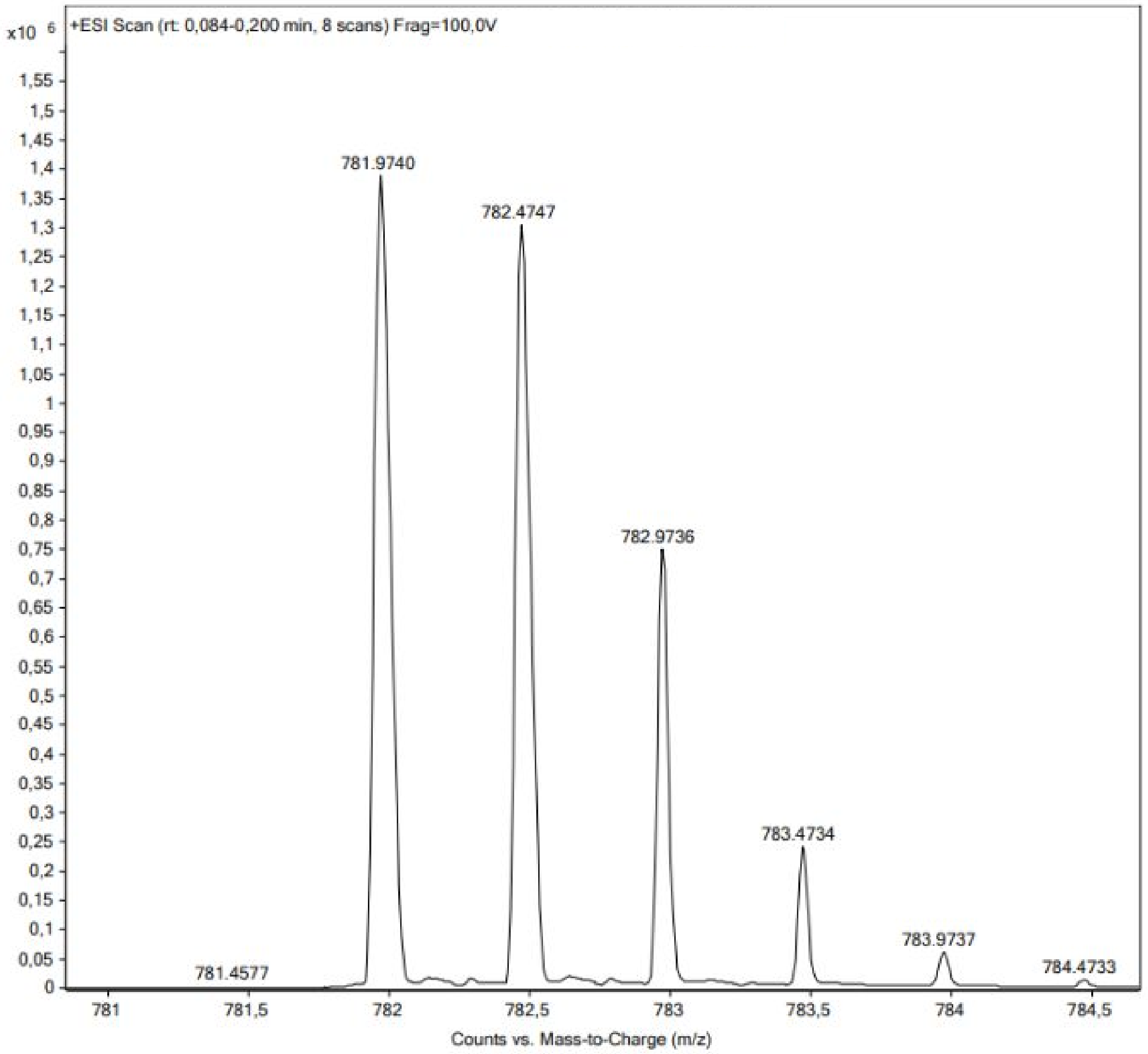
x10 6
+ESI Scan (rt: 0,084-0,200 min, 8 scans) Frag=100,0V
781.9740
782.4747
782.9736
783.4734
783.9737
784.4733
781.4577
Counts vs. Mass-to-Charge (m/z)

HV6

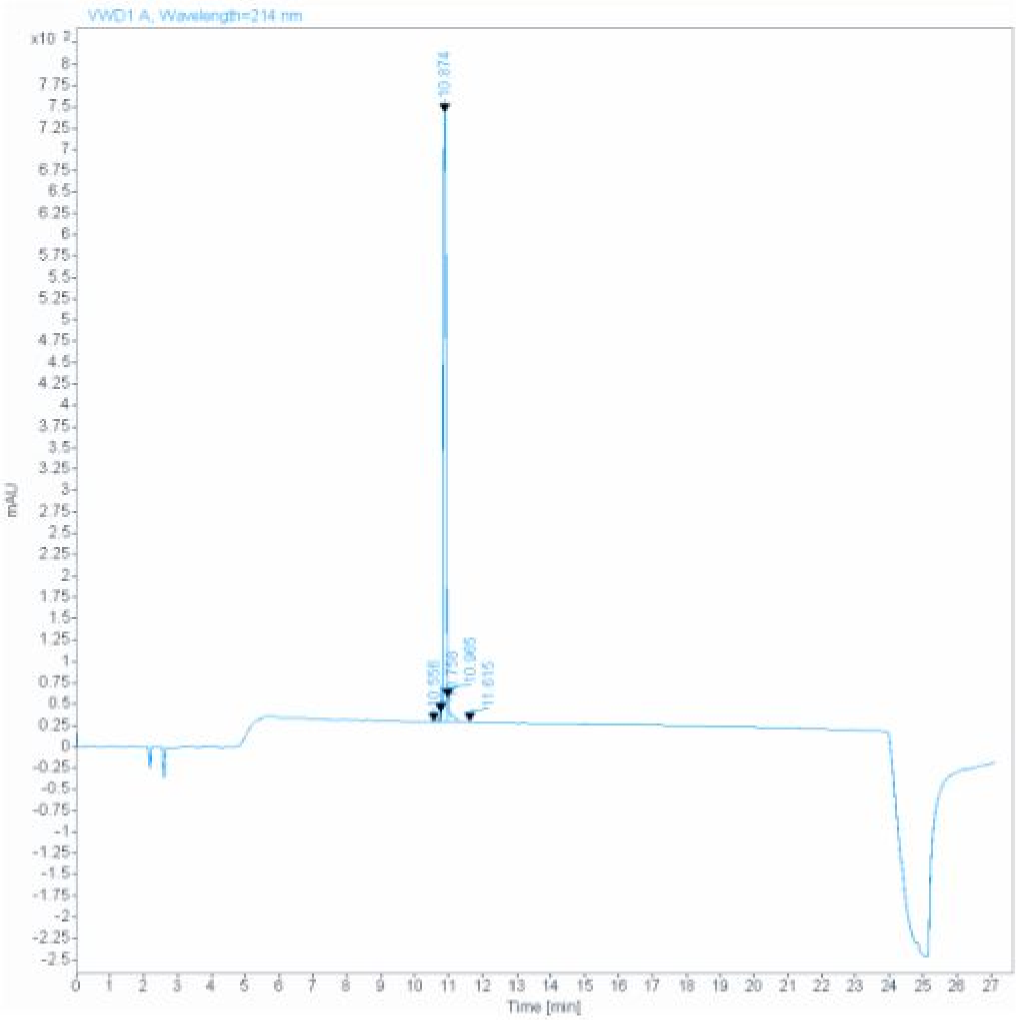

Signal: VWD1 A, Wavelength=214 nm

RT [min]	Type	Width [min]	Area	Height	Area%	Name
10.556	MM	0.0578	1.3972	0.4028	0.0325	
10.758	MF	0.0495	33.7174	11.3568	0.7847	
10.874	MF	0.0956	4101.8745	714.9889	95.4658	
10.965	FM	0.0768	157.0865	27.1655	3.6560	
11.615	MM	0.0446	2.6218	0.9803	0.0610	
		Sum	4296.6974			

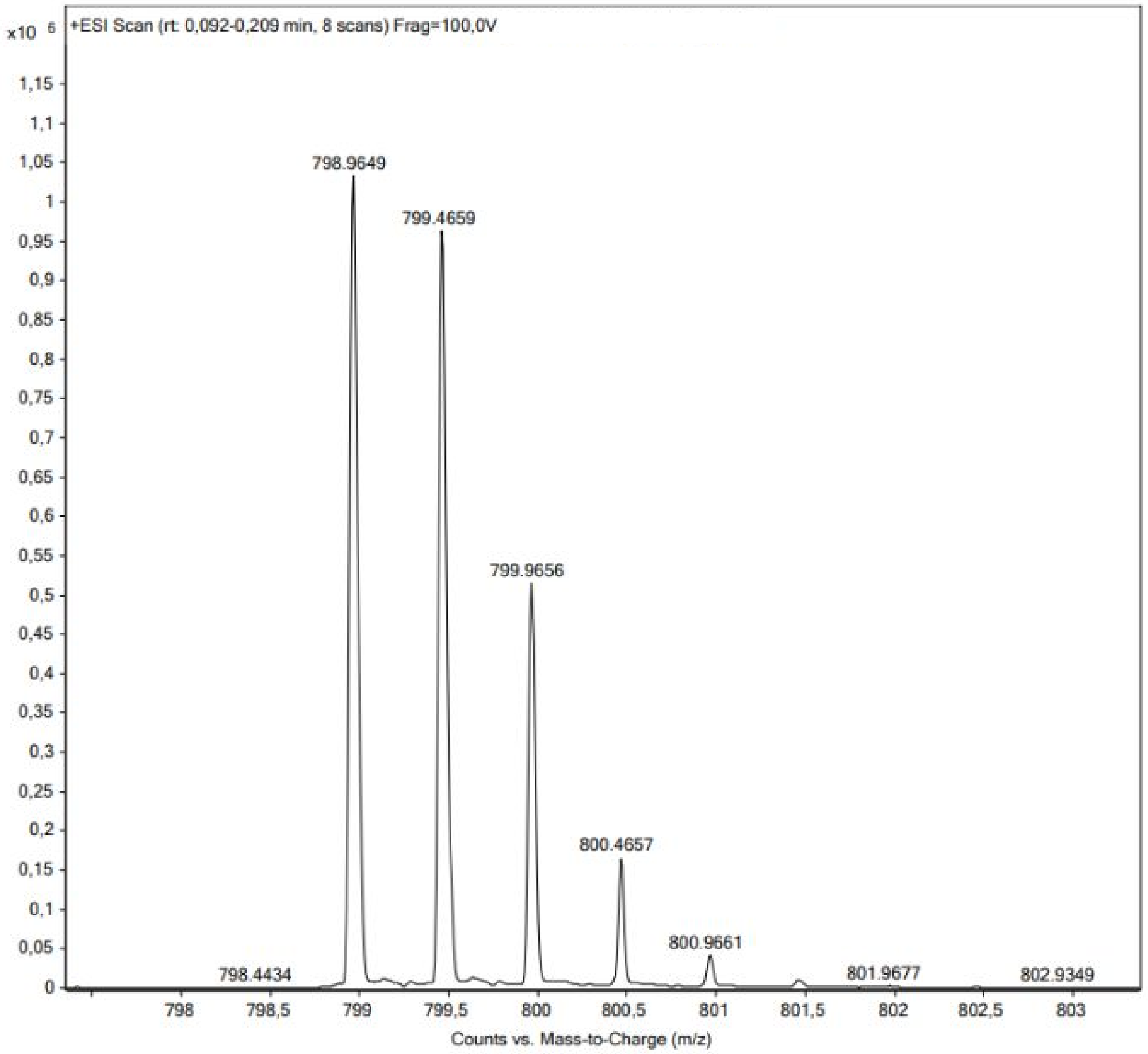

x10 6
+ESI Scan (rt: 0,092-0,209 min, 8 scans) Frag=100,0V
798.9649
799.4659
799.9656
800.4657
800.9661
798.4434
801.9677
802.9349
Counts vs. Mass-to-Charge (m/z)

SV1

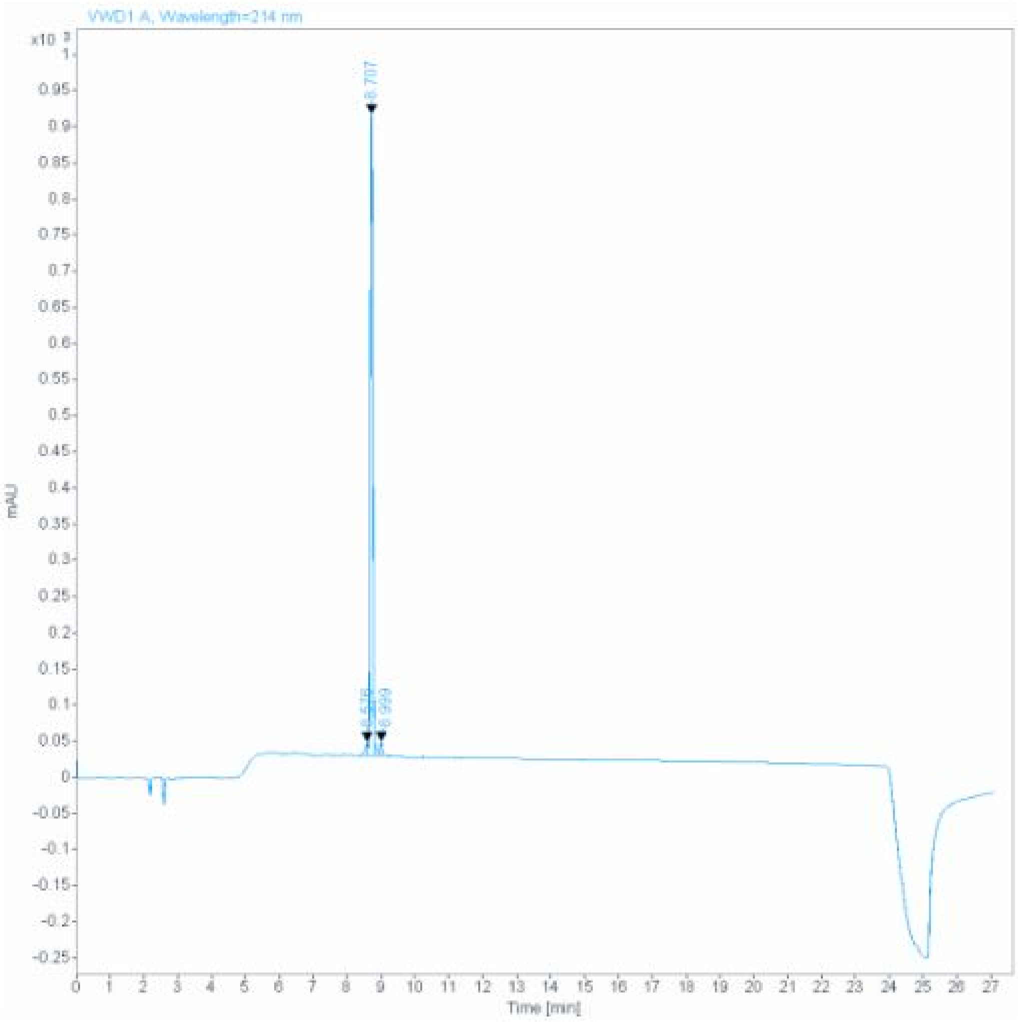

Signal: VWD1 A, Wavelength=214 nm

RT [min]	Type	Width [min]	Area	Height	Area%	Name
8.576	MF	0.0905	110.9435	20.4422	2.3158	
8.707	MF	0.0852	4546.0771	889.7848	94.8954	
8.999	FM	0.1057	133.5972	21.0579	2.7887	
		Sum	4790.6178			

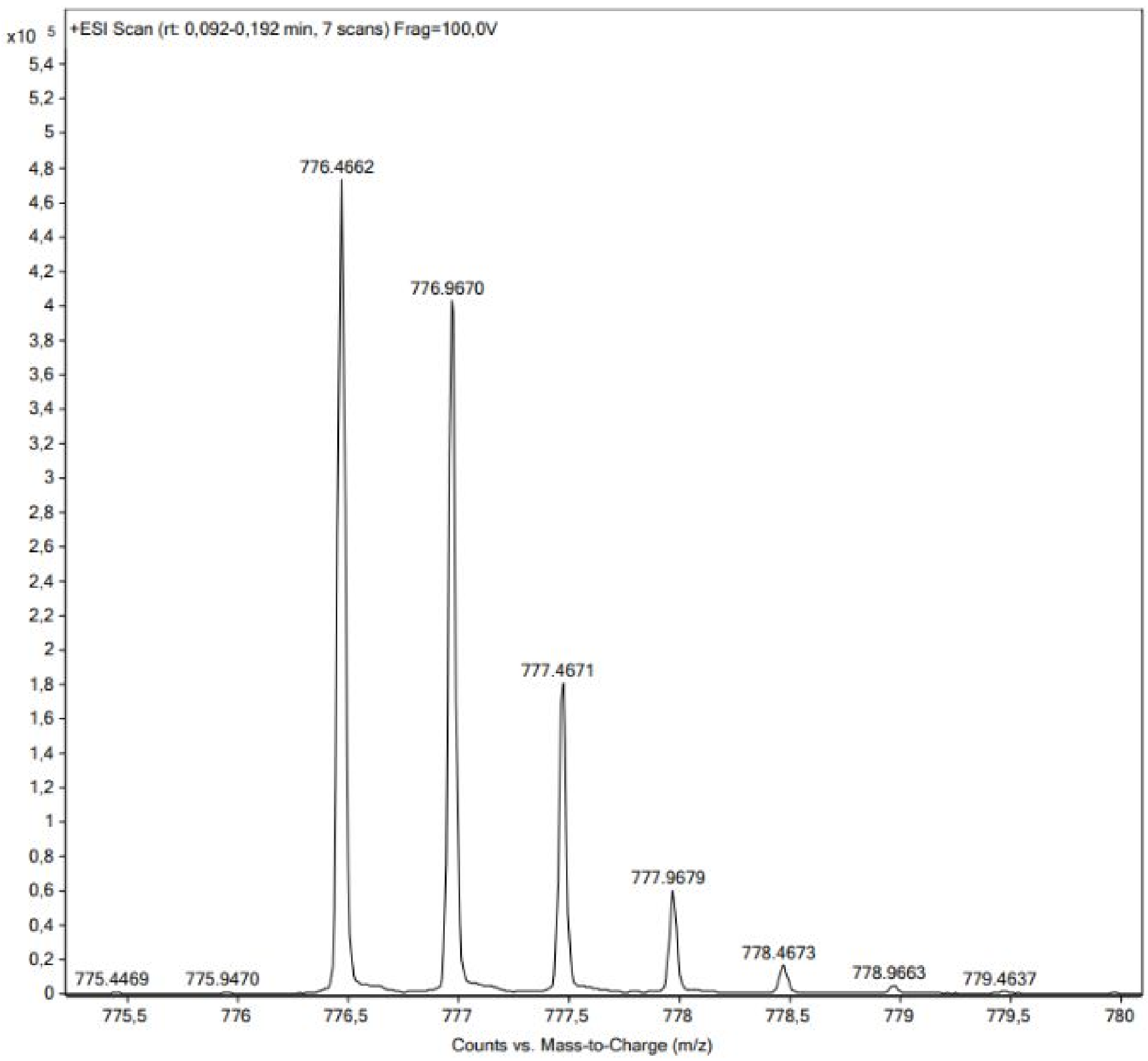

x10 5
+ESI Scan (rt: 0,092-0,192 min, 7 scans) Frag=100,0V
5,4
5,2
5
4,8
4,6
4,4
4,2
4
3,8
3,6
3,4
3,2
3
2,8
2,6
2,4
2,2
2
1,8
1,6
1,4
1,2
1
0,8
0,6
0,4
0,2
0
776.4662
776.9670
777.4671
777.9679
778.4673
778.9663
779.4637
775.4469
775.9470
775,5
776
776,5
777
777,5
778
778,5
779
779,5
780
Counts vs. Mass-to-Charge (m/z)

Unconjugated Bicycle “warhead” and original standalone Map chromatographic and mass spectrometry data

AB1

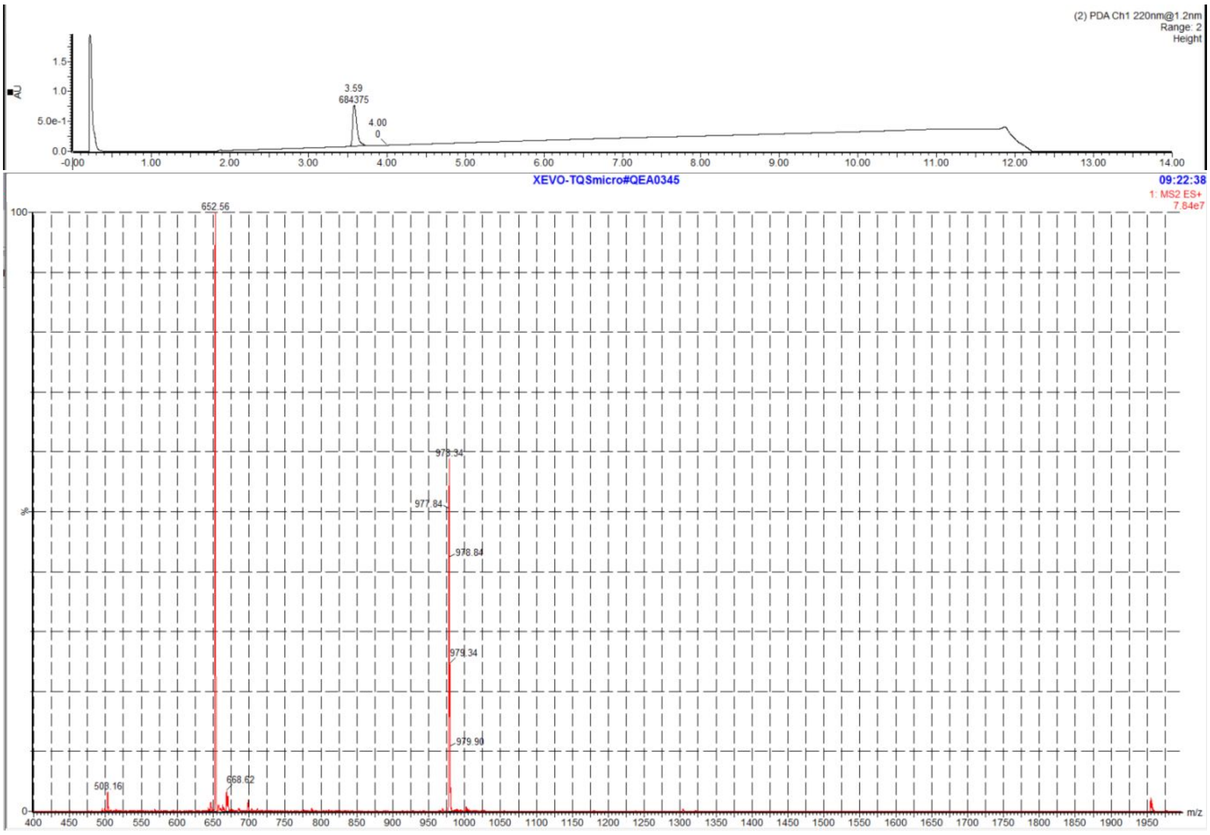

AV1

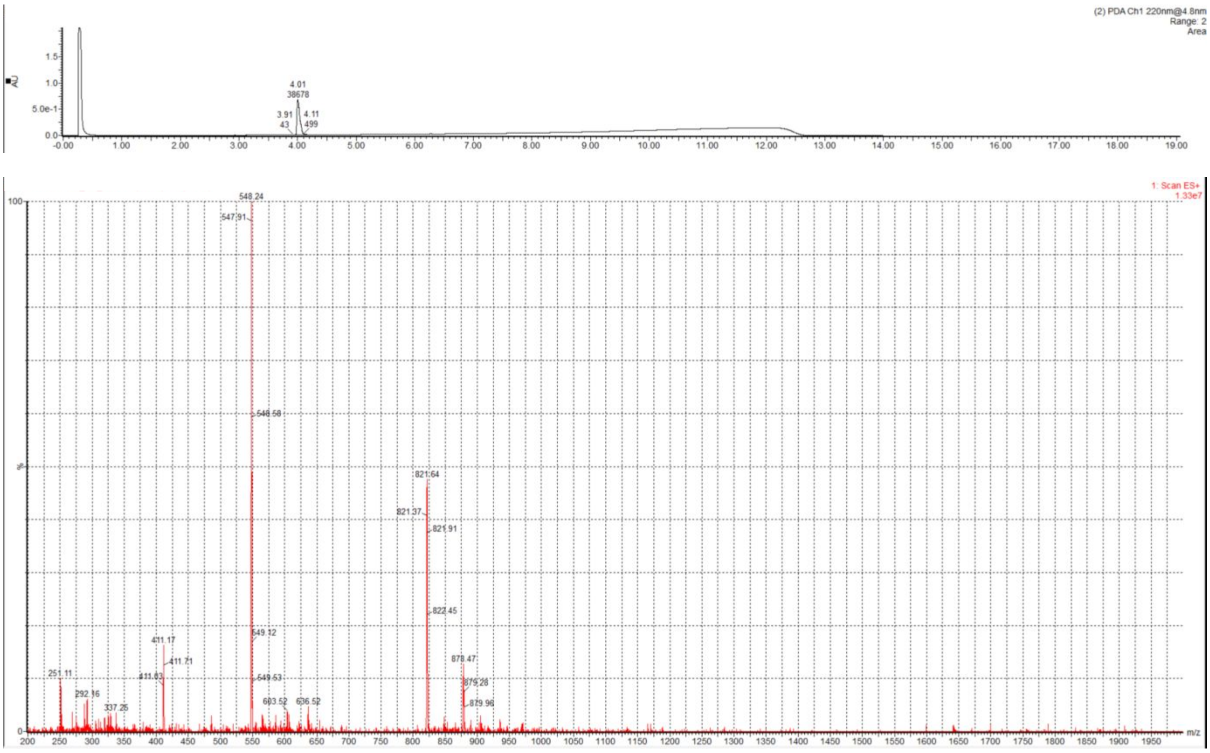

Bicycle-vector conjugates chromatographic and mass spectrometry data

A1

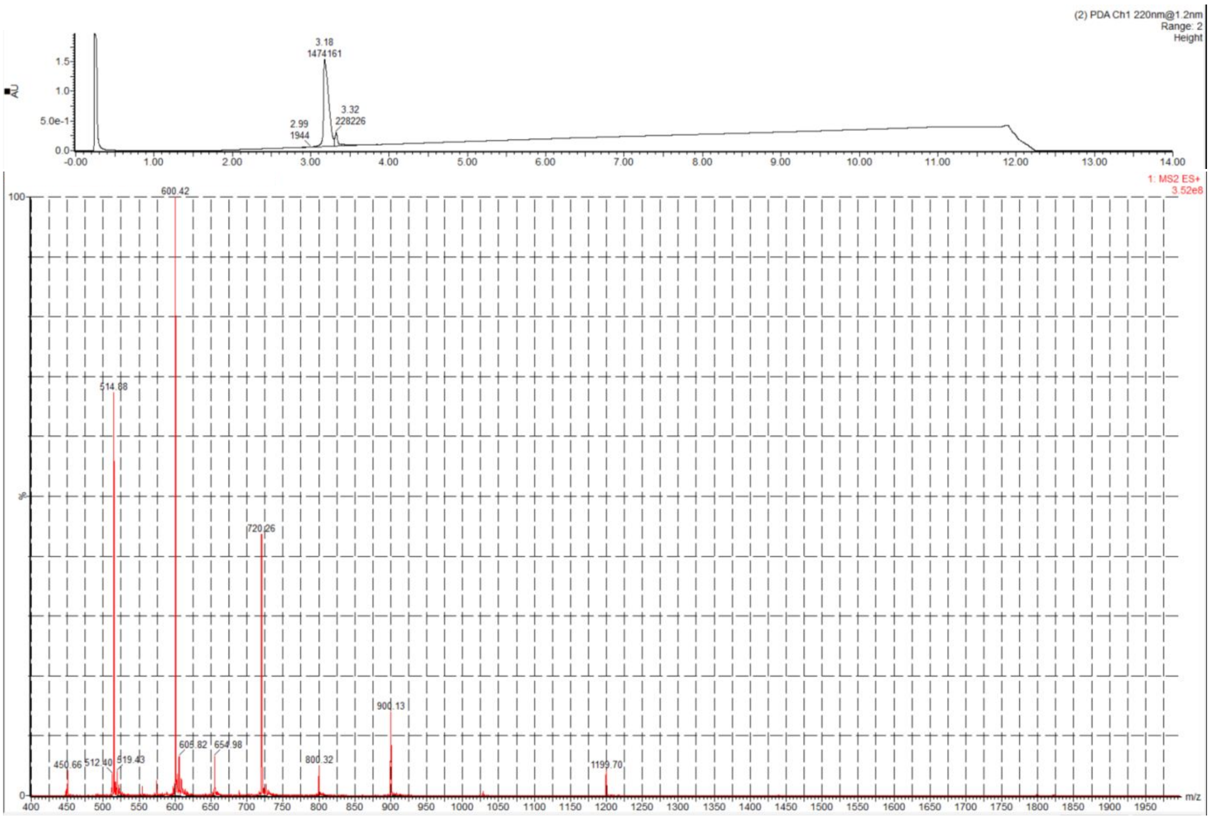

S1

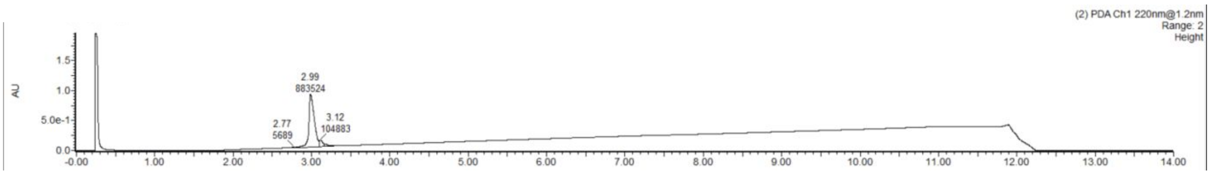

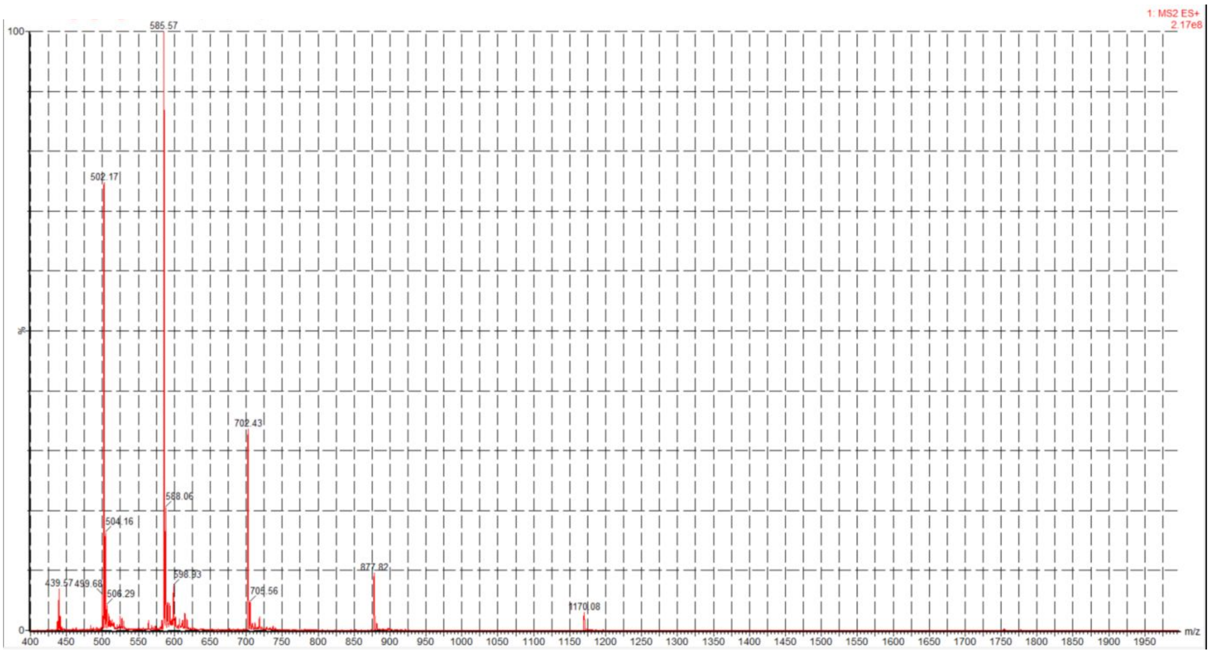
1: MS2 ES+
2.17e8
585.57
502.17
702.43
588.06
504.16
598.93
877.82
439.57
459.68
506.29
705.56
1170.08
m/z

H6

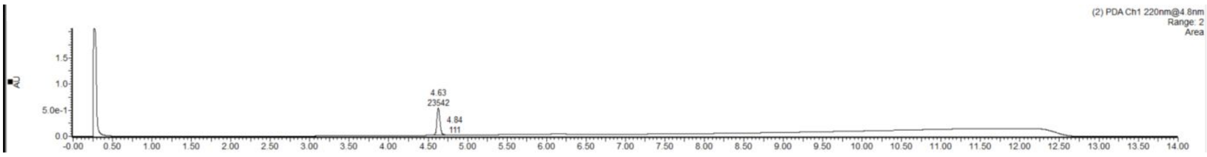
(2) PDA Ch1 220nm@4.8nm
Range: 2
Area
AU
4.63
23542
4.84
111

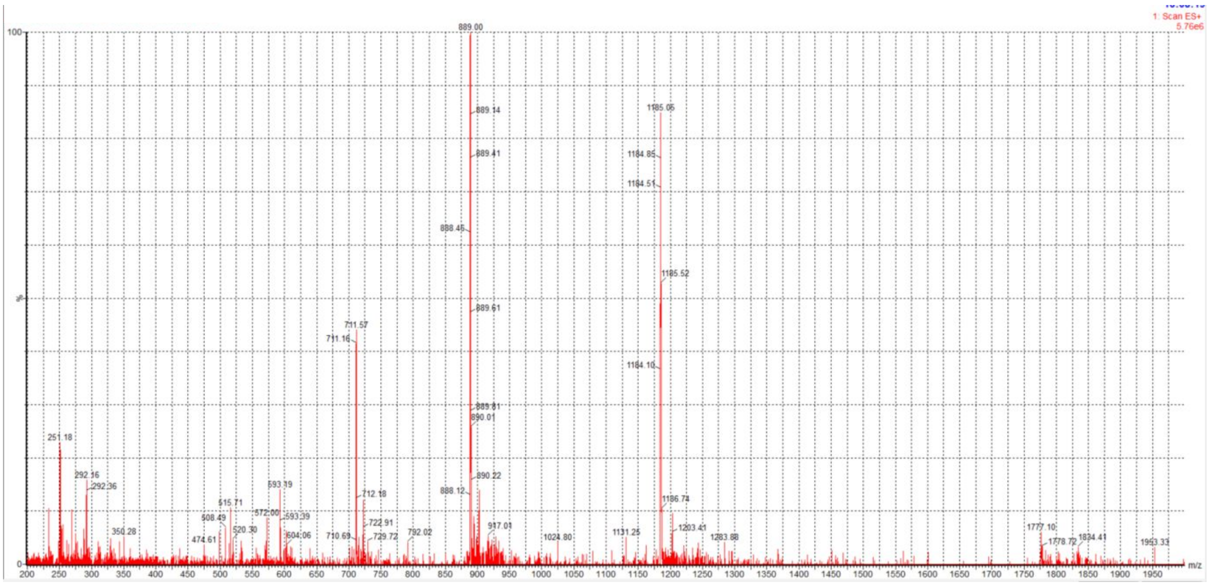
1: Scan ES+
5.76e6
889.00
889.14
889.41
888.45
889.61
889.81
890.01
890.22
888.12
917.01
1185.06
1184.85
1184.51
1185.52
1184.10
1186.74
1203.41
1283.88
711.57
711.16
712.18
722.91
729.72
710.69
792.02
251.18
292.16
292.36
350.28
474.61
508.49
515.71
520.30
572.00
593.19
593.39
604.06
1024.80
1131.25
1777.10
1778.72
1834.41
1953.33
m/z

H5

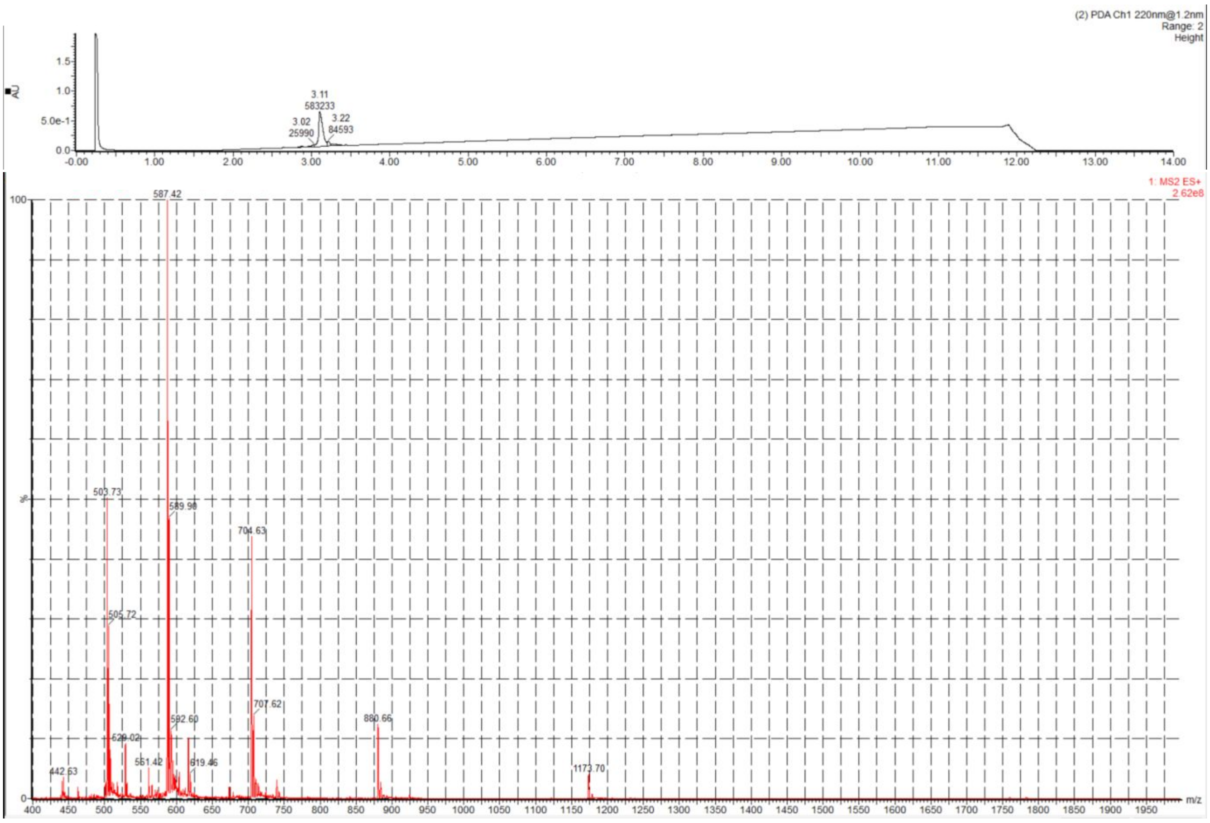
(2) PDA Ch1 220nm@1.2nm
Range: 2
Height
AU
3.11
583233
3.02
25990
3.22
84593
1: MS2 ES+
2.62e8
587.42
503.73
589.98
704.63
505.72
707.62
592.60
880.66
529.02
561.42
619.46
442.63
1173.70
%
m/z

H4

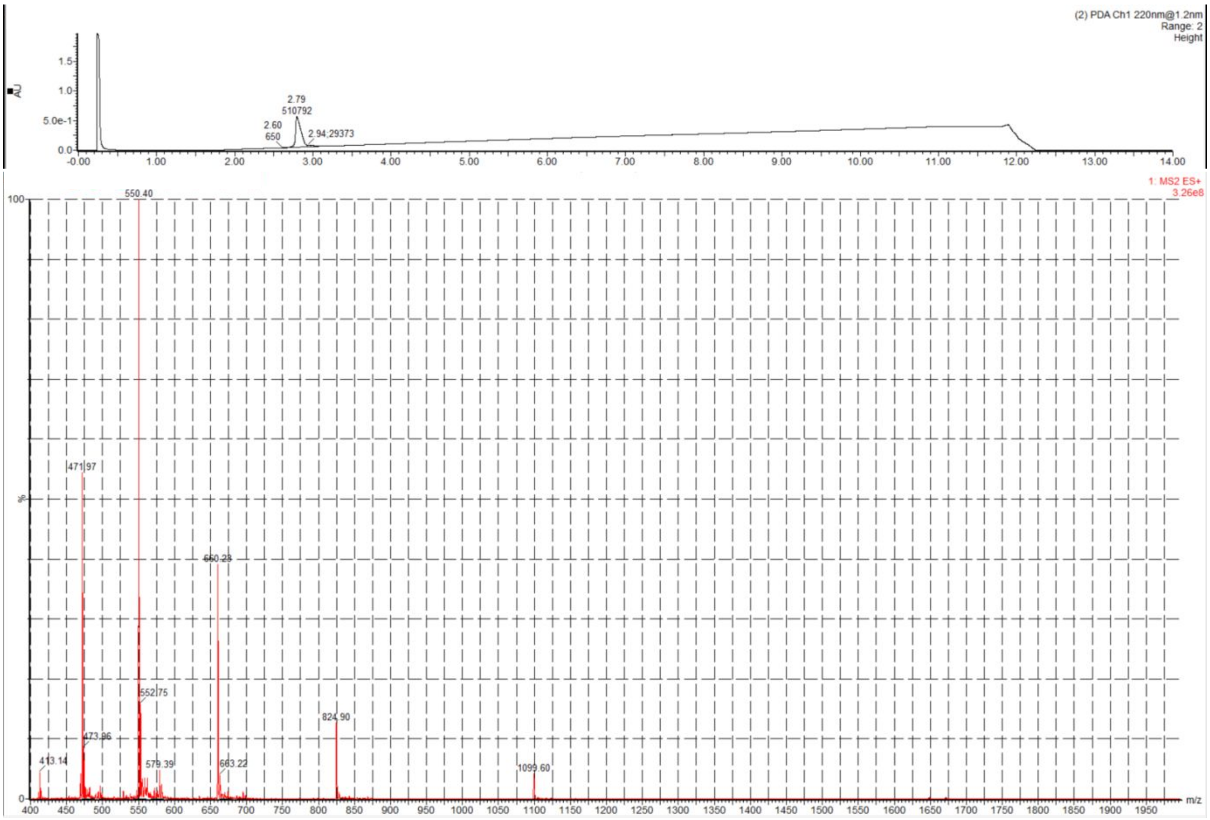
(2) PDA Ch1 220nm@1.2nm
Range: 2
Height
AU
2.79
510792
2.60
650
2.94,29373
1: MS2 ES+
3.26e8
550.40
471.97
660.23
552.75
824.90
473.86
413.14
579.39
663.22
1099.60
%
m/z

H3

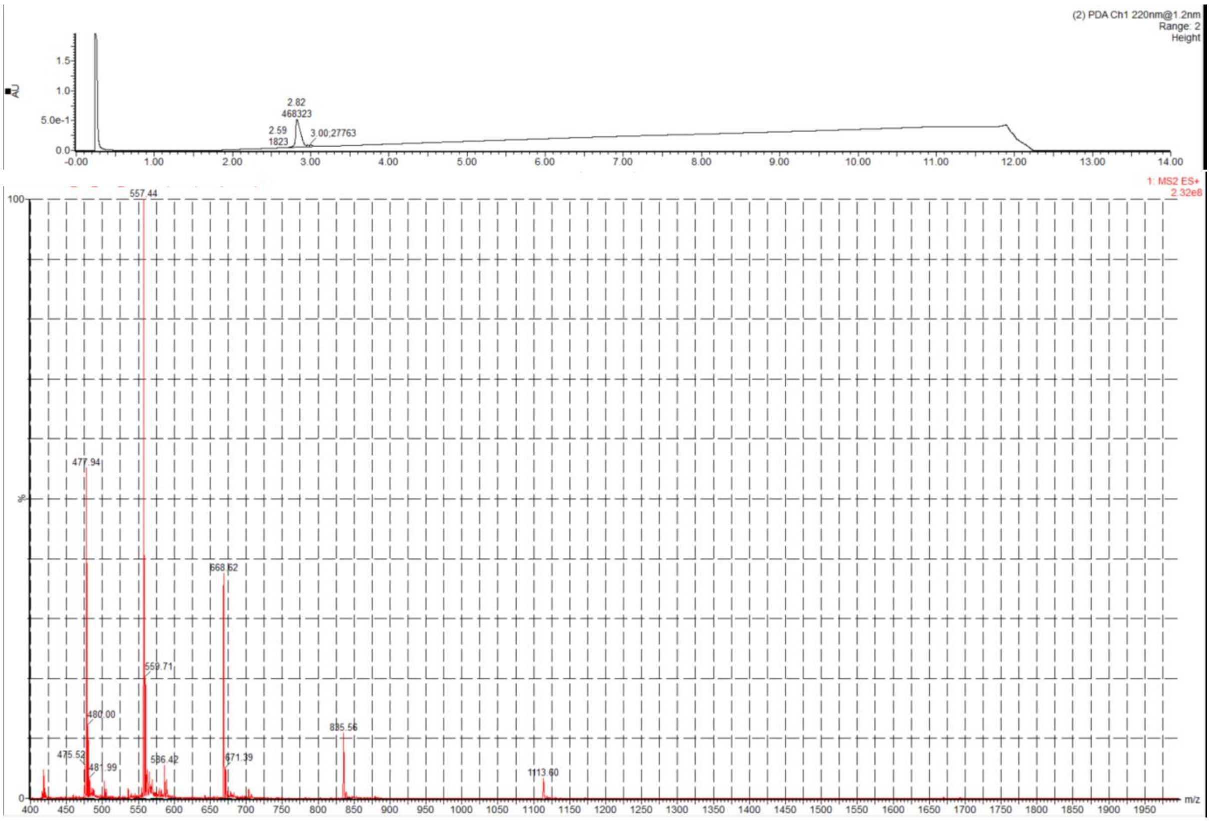
(2) PDA Ch1 220nm@1.2nm
Range: 2
Height
AU
2.82
468323
2.59
1823
3.00;27763
1: MS2 ES+
2.32e6
557.44
477.94
668.62
559.71
480.00
835.56
475.52
586.42
671.39
481.99
1113.60
%
m/z

H2

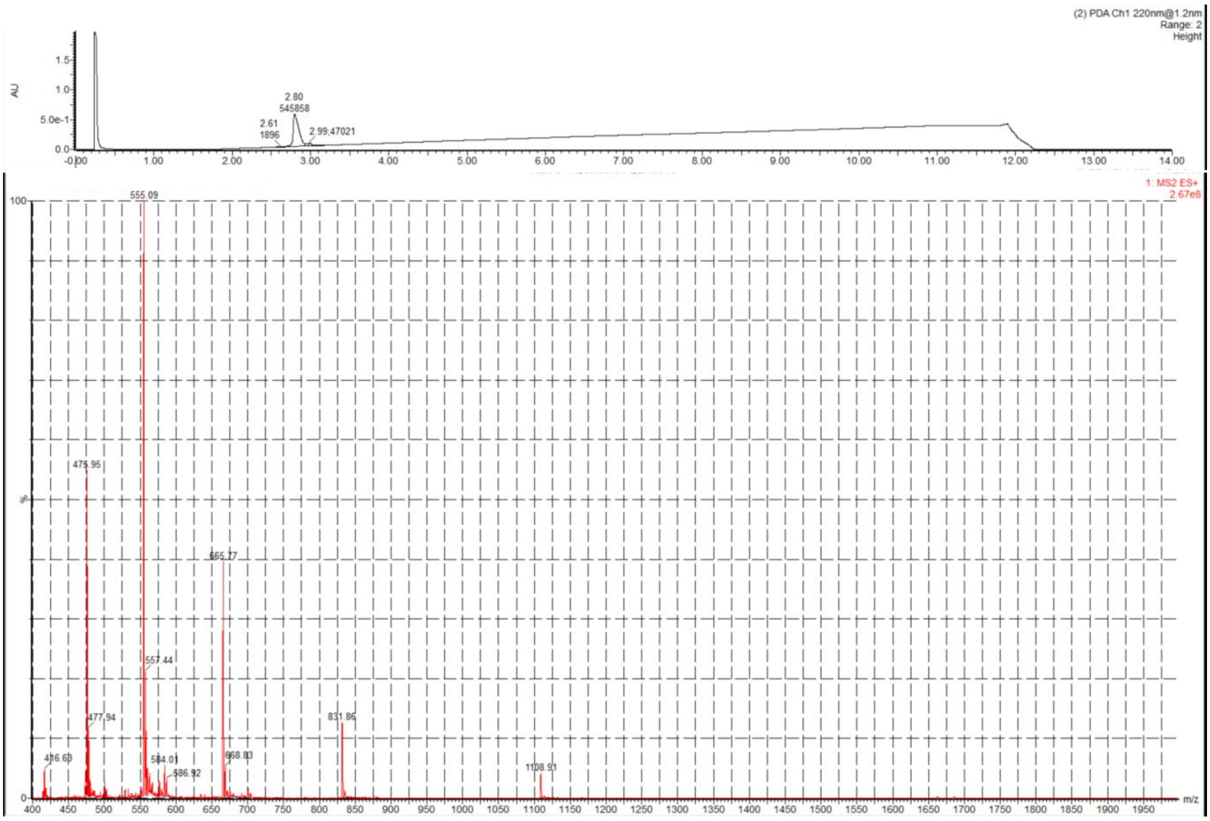
(2) PDA Ch1 220nm@1.2nm
Range: 2
Height
AU
2.80
545858
2.61
1896
2.99;47021
1: MS2 ES+
2.67e6
555.09
475.95
665.77
557.44
477.94
831.86
416.63
584.01
668.83
586.92
1108.91
%
m/z

H1

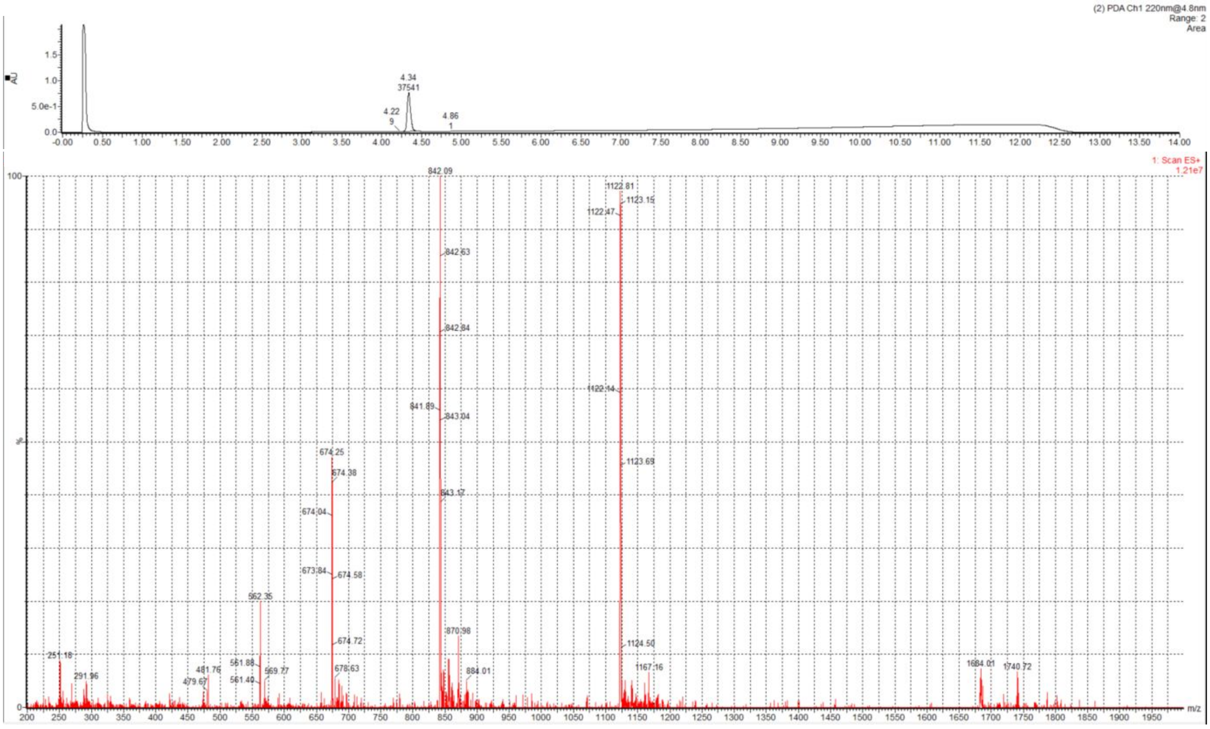

C11

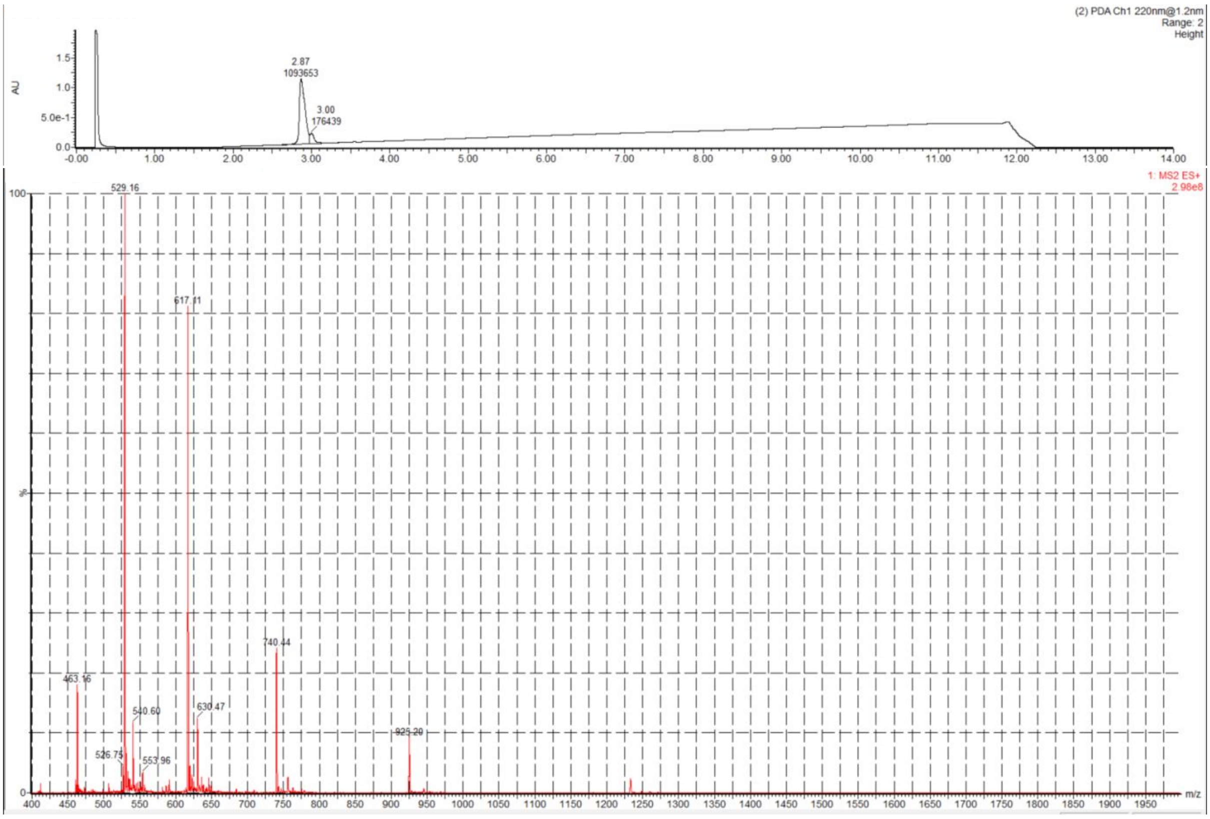

C10

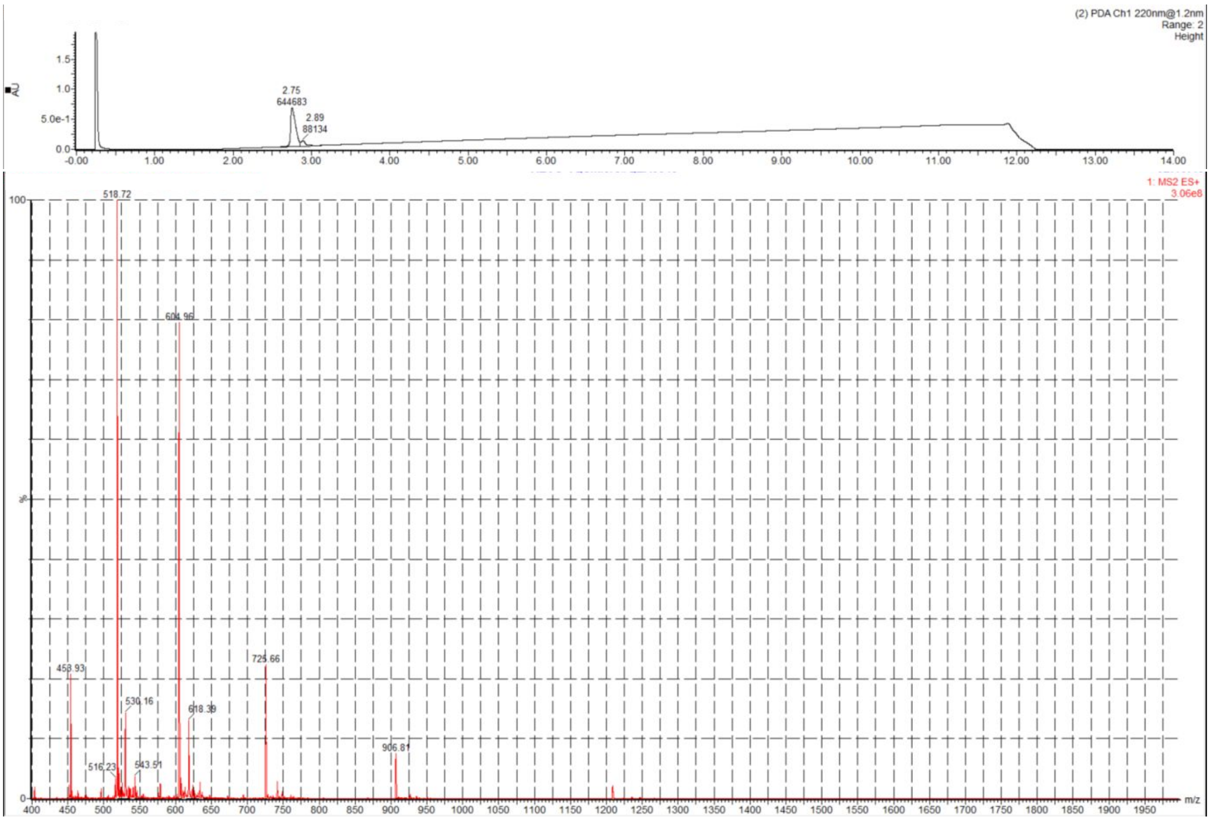
(2) PDA Ch1 220nm@1.2nm
Range: 2
Height
AU
2.75
644683
2.89
88134
1: MS2 ES+
3.06e8
518.72
604.96
453.93
725.66
530.16
618.39
906.81
516.23
543.51
%
m/z

C9

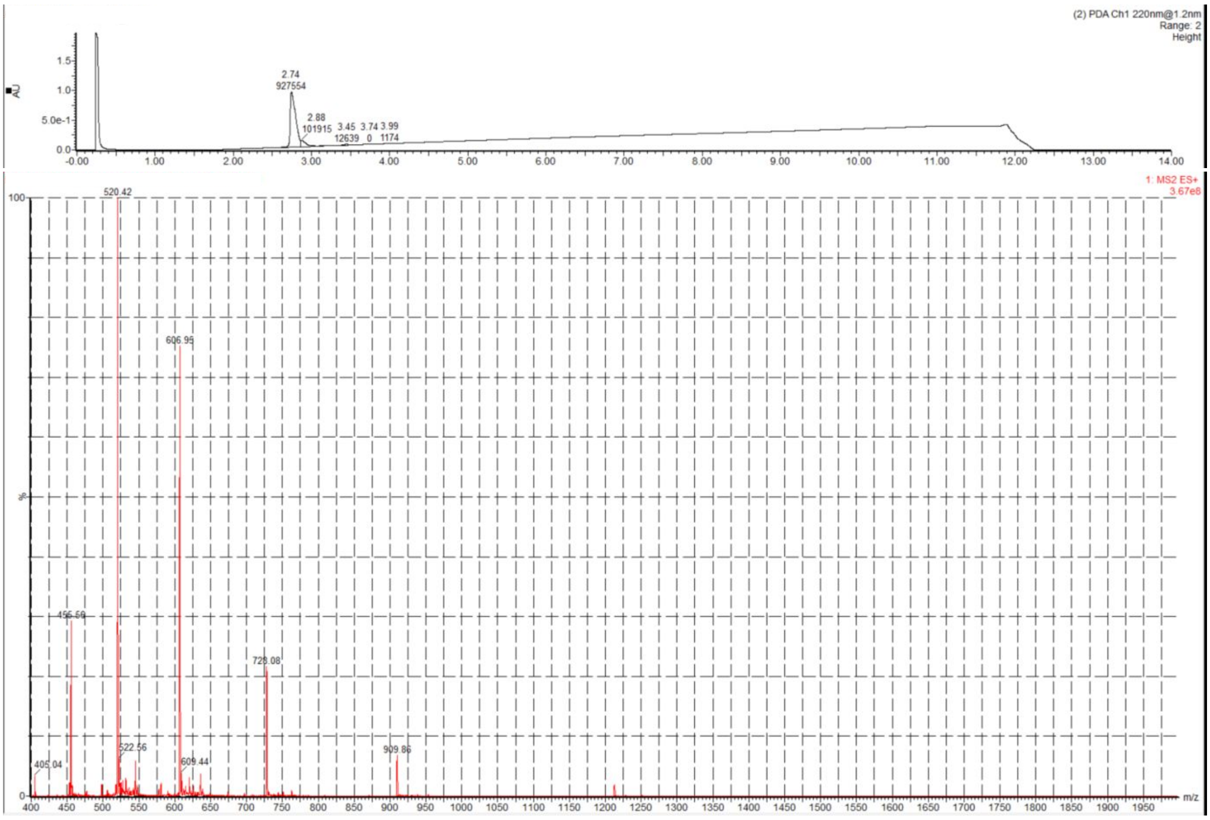
(2) PDA Ch1 220nm@1.2nm
Range: 2
Height
AU
2.74
927554
2.88
101915
3.45
12639
3.74
0
3.99
1174
1: MS2 ES+
3.67e8
520.42
606.95
456.59
728.08
522.56
609.44
909.86
405.04
%
m/z

C8

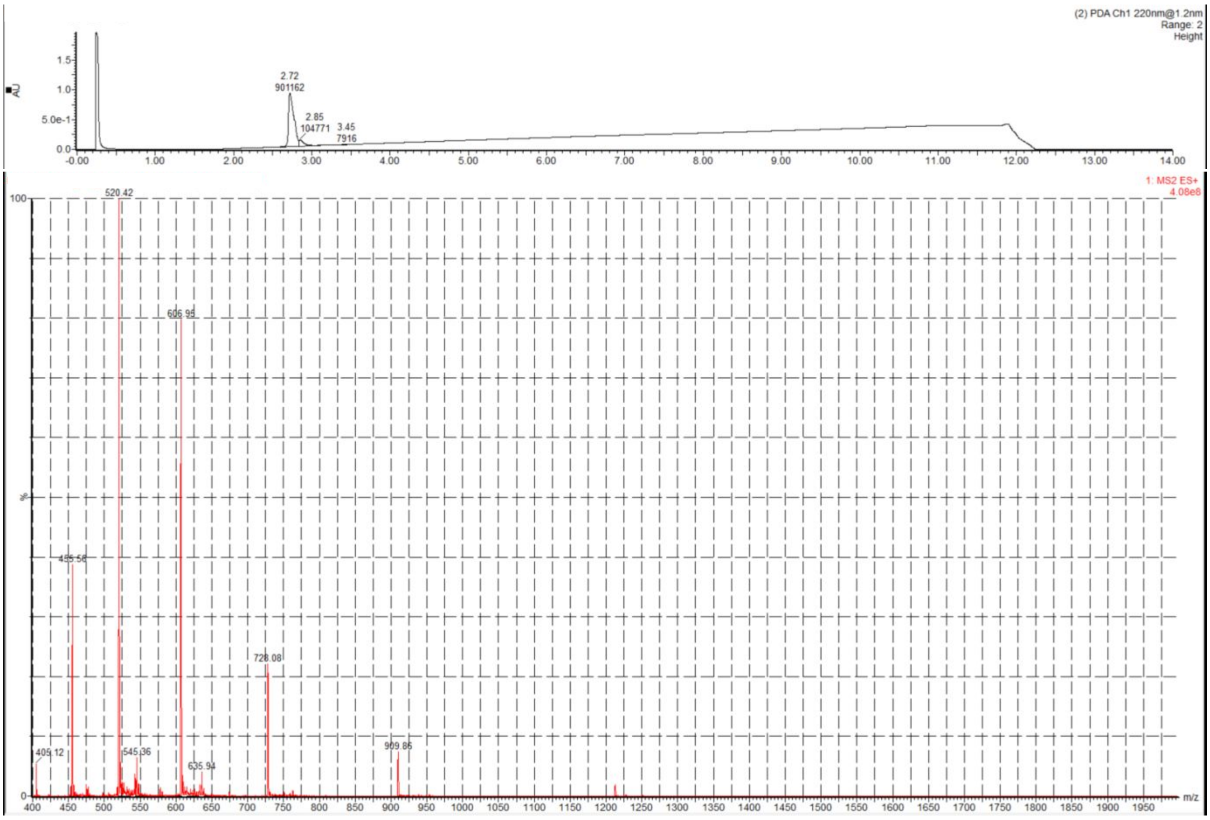
(2) PDA Ch1 220nm@1.2nm
Range: 2
Height
AU
2.72
901162
2.85
104771
3.45
7916
1: MS2 ES+
4.08e8
520.42
605.95
728.08
909.86
405.12
545.36
635.94
m/z
%

C5

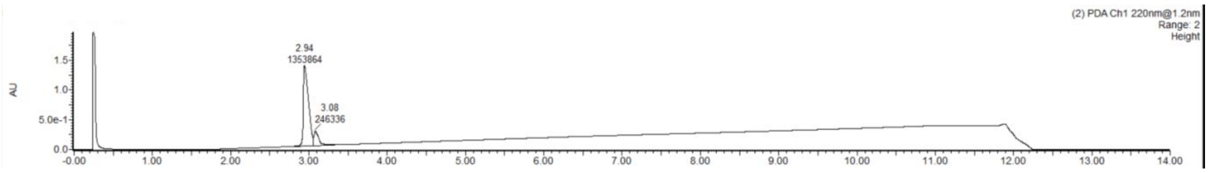
(2) PDA Ch1 220nm@1.2nm
Range: 2
Height
AU
2.94
1353864
3.08
246336

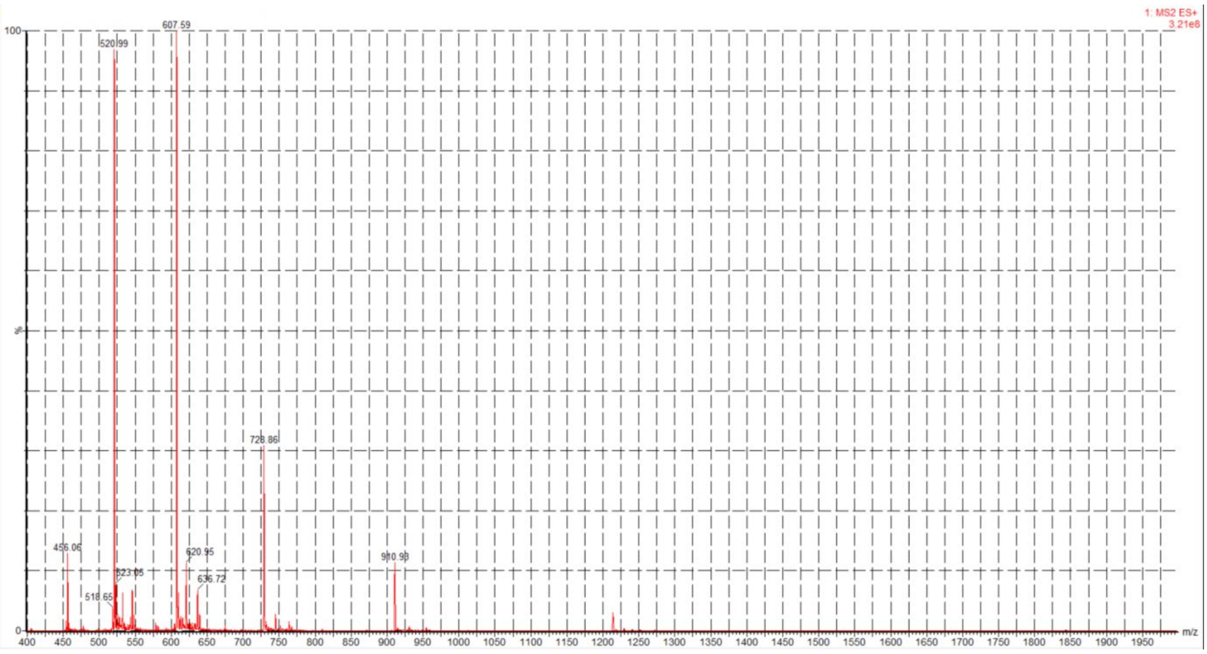
1: MS2 ES+
3.21e8
100
%
0
607.59
520.99
728.86
456.06
518.65
523.05
620.95
636.72
910.93
m/z

S4

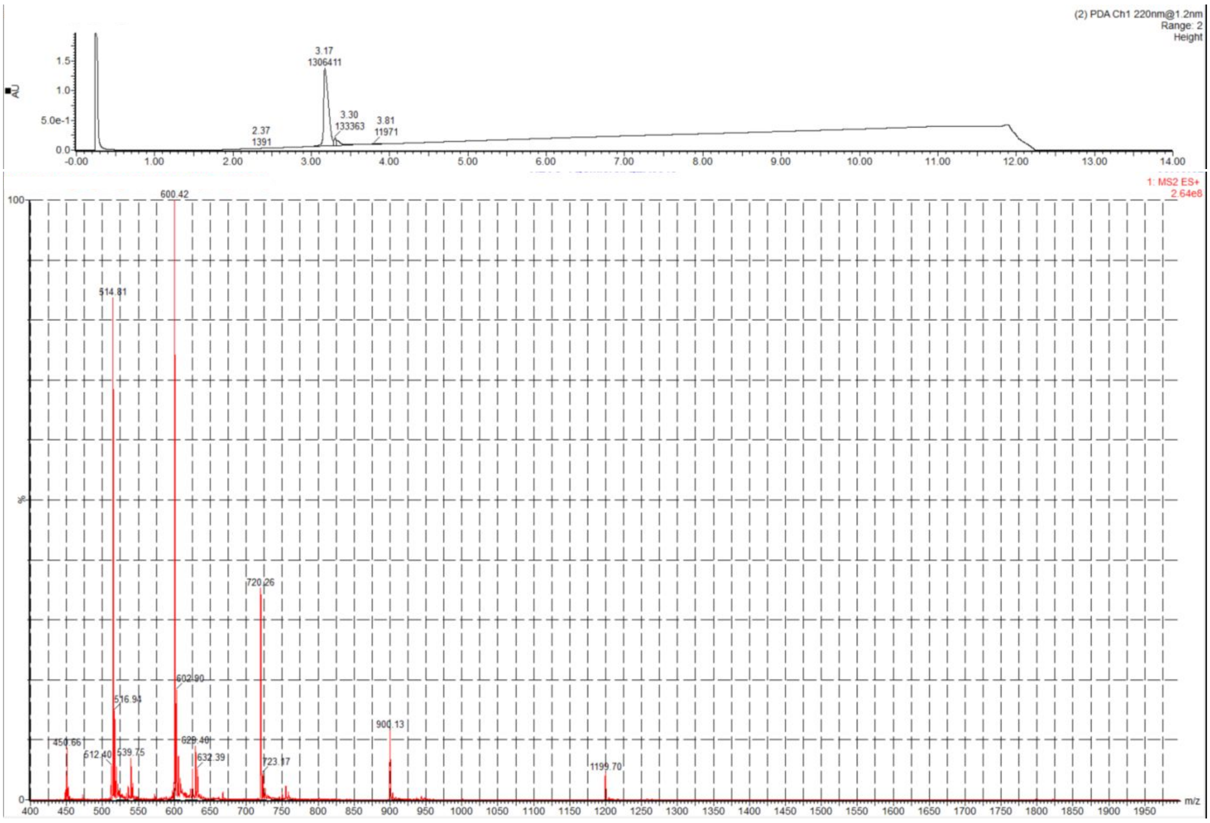
(2) PDA Ch1 220nm@1.2nm
Range: 2
Height
AU
3.17
1306411
2.37
1391
3.30
133363
3.81
11971
1: MS2 ES+
2.64e8
100
%
0
600.42
514.81
720.26
602.90
516.94
450.66
512.40
539.75
629.46
632.39
723.17
900.13
1199.70
m/z

S3

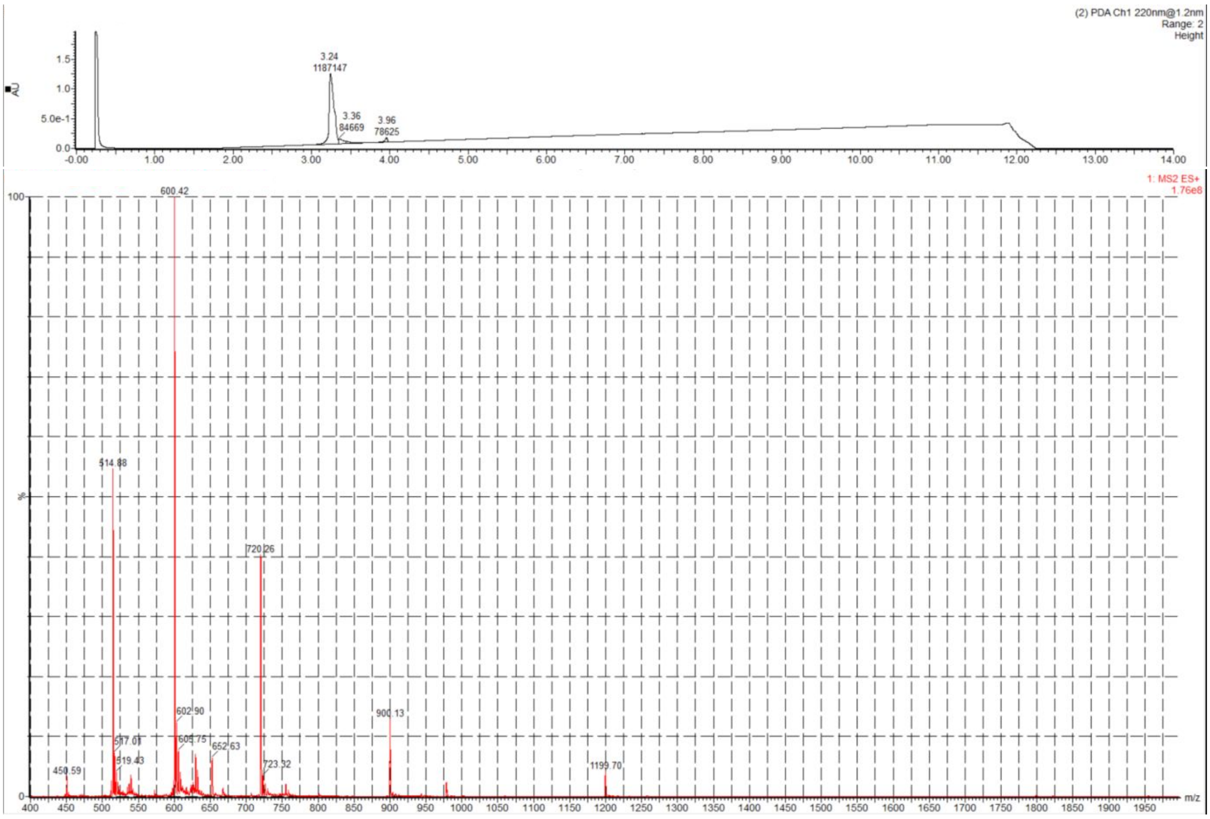
(2) PDA Ch1 220nm@1.2nm
Range: 2
Height
AU
3.24
1187147
3.36
84669
3.96
78625
1: MS2 ES+
1.76e8
600.42
514.88
720.26
602.90
900.13
605.75
652.63
723.32
450.59
1199.70
m/z

S2

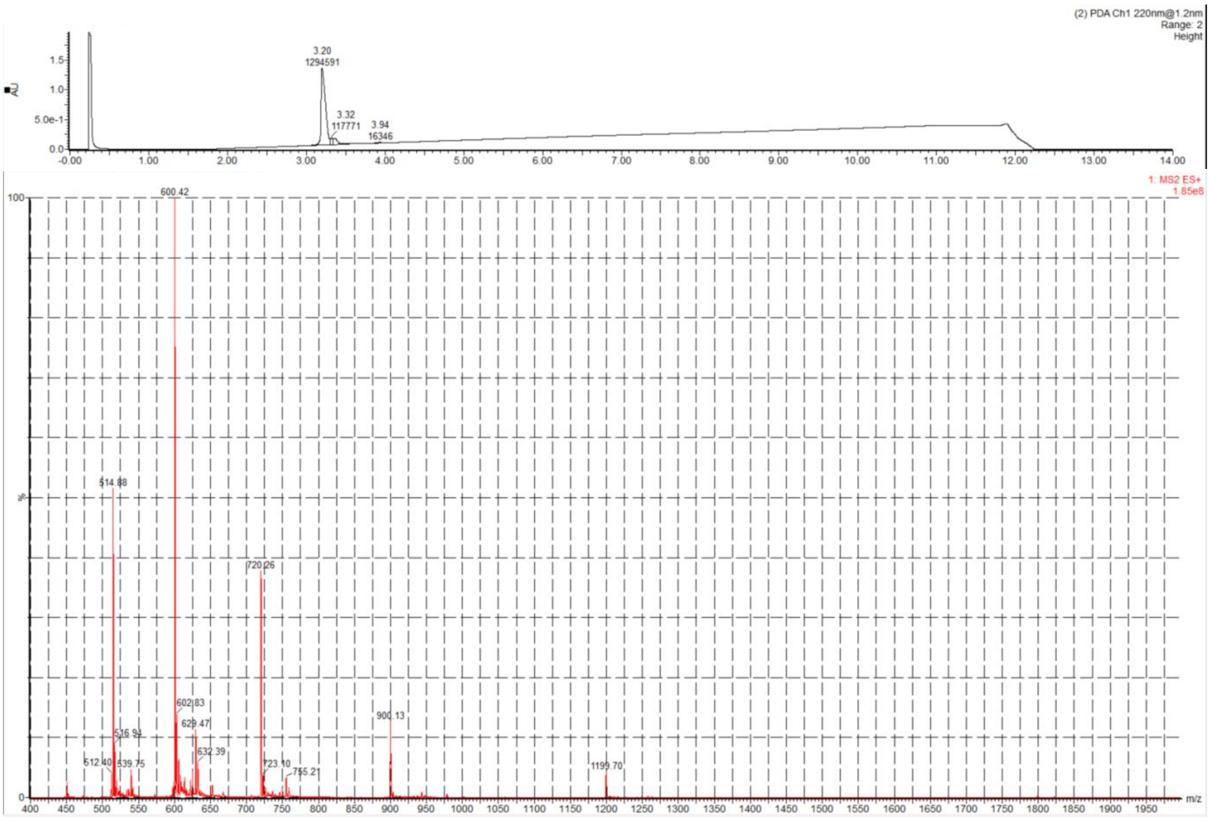
(2) PDA Ch1 220nm@1.2nm
Range: 2
Height
AU
3.20
1294591
3.32
117771
3.94
16346
1: MS2 ES+
1.85e8
600.42
514.88
720.26
602.83
900.13
629.47
632.39
512.40
539.75
723.10
755.21
1199.70
m/z

C7

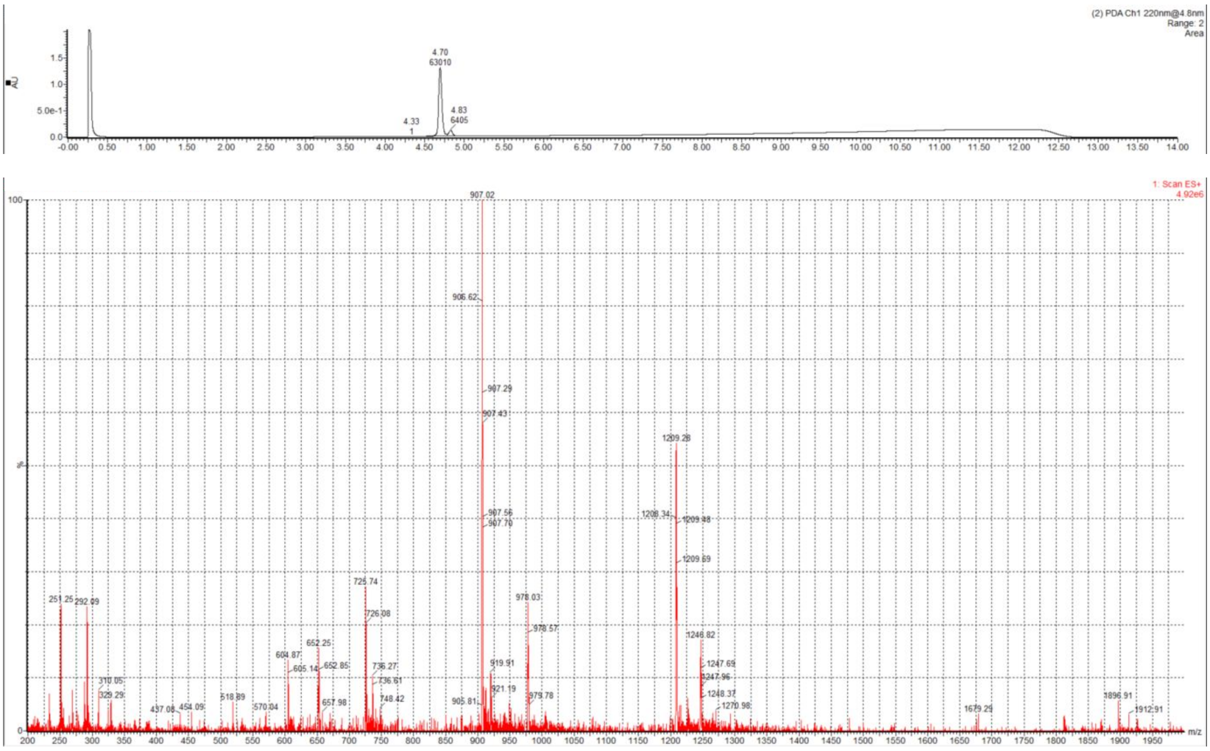
(2) PDA Ch1 220nm@4.8nm
Range: 2
Area
AU
4.70
63010
4.33
1
4.83
6405
1: Scan ES+
4.92e6
907.02
906.62
907.29
907.43
907.56
907.70
1209.28
1208.34
1209.48
1209.69
725.74
726.08
978.03
978.57
251.25
292.09
604.87
605.14
652.25
652.85
736.27
736.61
748.42
919.91
921.19
905.81
979.78
1246.82
1247.69
1247.96
1248.37
1270.98
318.05
329.29
437.08
454.09
518.89
570.04
657.98
1679.29
1896.91
1912.91
%
m/z

C1

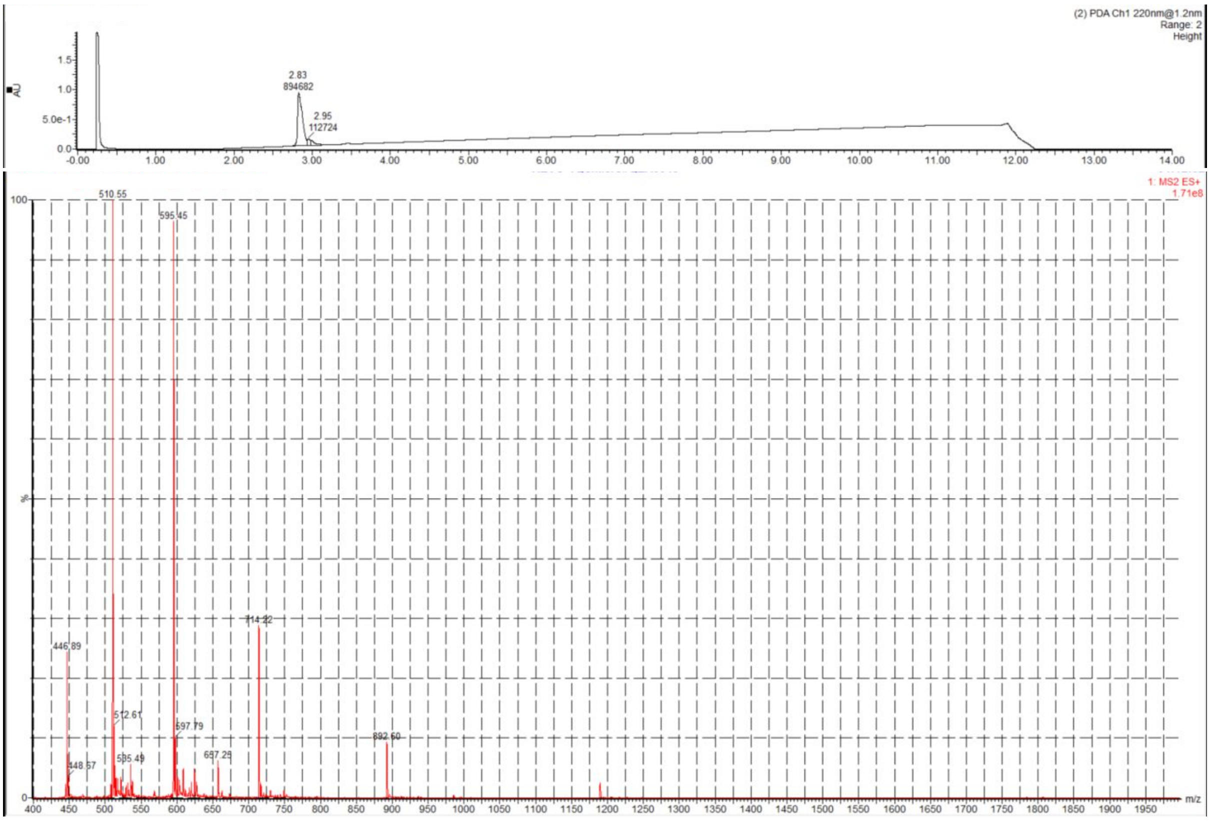
(2) PDA Ch1 220nm@1.2nm
Range: 2
Height
AU
2.83
894682
2.95
112724
1: MS2 ES+
1.71e8
510.55
595.45
714.22
446.89
512.61
597.79
892.60
448.57
535.49
657.25
%
m/z

C2

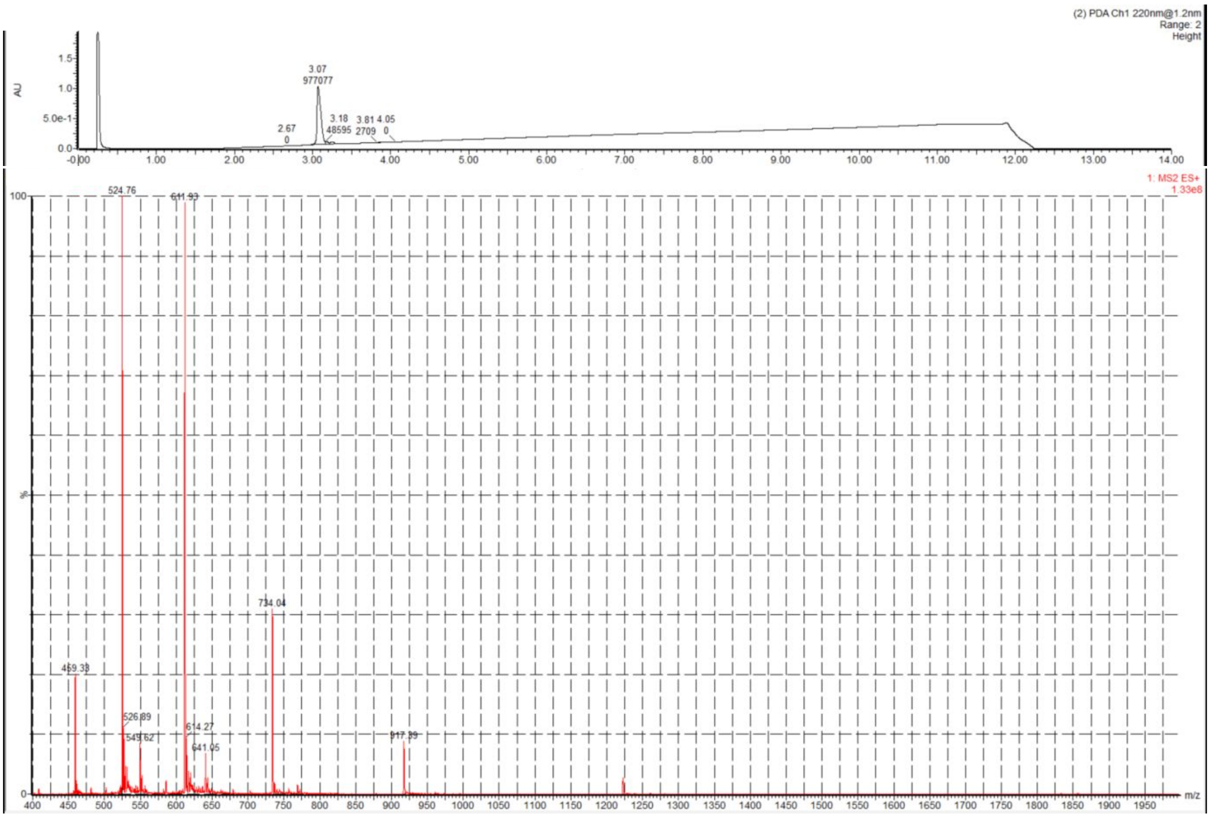
(2) PDA Ch1 220nm@1.2nm
Range: 2
Height
AU
3.07
977077
3.18
48595
2.67
0
3.81
2709
4.05
0
1: MS2 ES+
1.33e8
524.76
611.93
734.04
459.33
526.89
549.62
614.27
641.05
917.39
%
m/z

C3

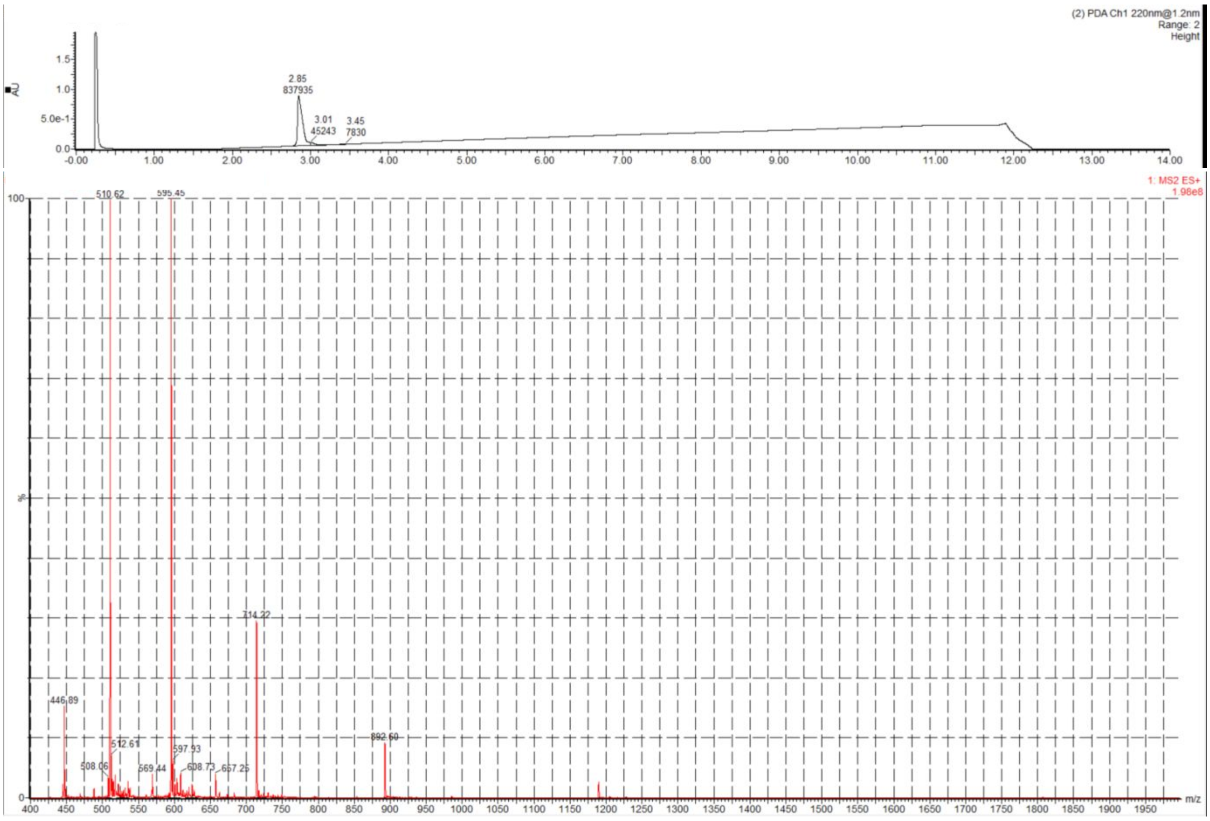
(2) PDA Ch1 220nm@1.2nm
Range: 2
Height
AU
2.85
837935
3.01
45243
3.45
7830
1: MS2 ES+
1.98e8
510.62
595.45
714.22
446.89
892.60
512.61
508.06
569.44
597.93
608.73
657.25
%
m/z

C4

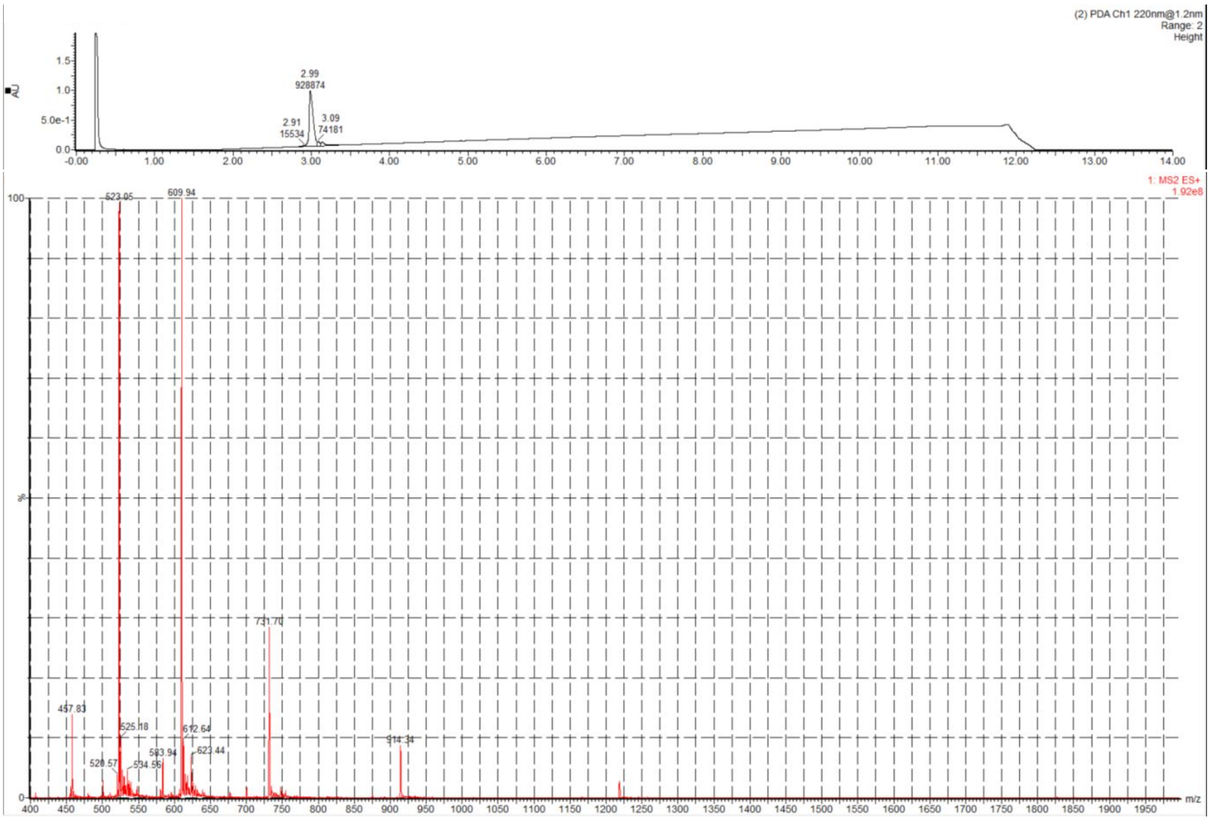

(2) PDA Ch1 220nm@1.2nm
Range: 2
Height
AU
2.99
928874
2.91
15534
3.09
74181
1: MS2 ES+
1.92e8
523.05
609.94
731.70
457.83
525.18
612.64
623.44
534.56
914.34
%
m/z

C6

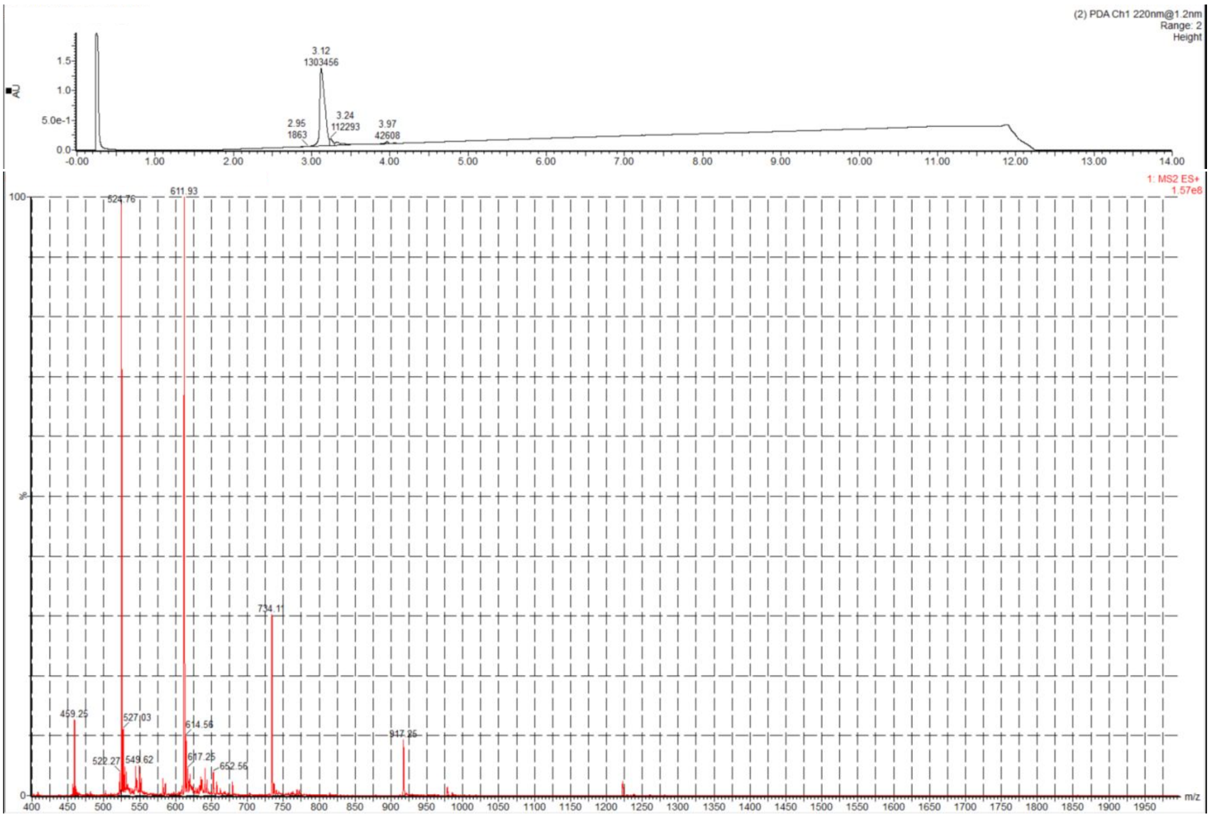

(2) PDA Ch1 220nm@1.2nm
Range: 2
Height
AU
3.12
1303456
2.95
1863
3.24
112293
3.97
42608
1: MS2 ES+
1.57e8
524.76
611.93
459.25
527.03
614.56
522.27
549.62
617.25
652.56
917.25
%
m/z

C12

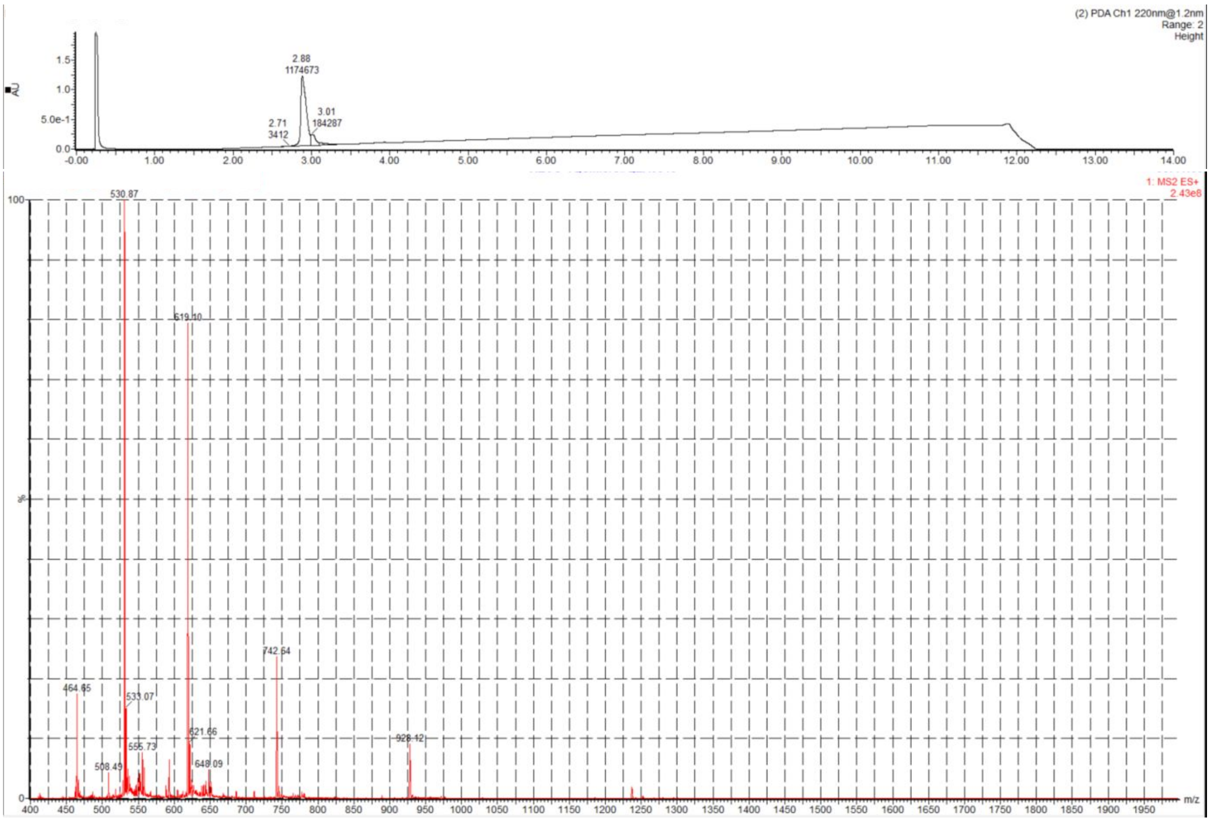
(2) PDA Ch1 220nm@1.2nm
Range: 2
Height
2.88
1174673
2.71
3412
3.01
184287
1: MS2 ES+
2.43e8
530.87
619.10
742.64
464.65
533.07
621.66
555.73
508.49
648.09
928.12
m/z

SD4

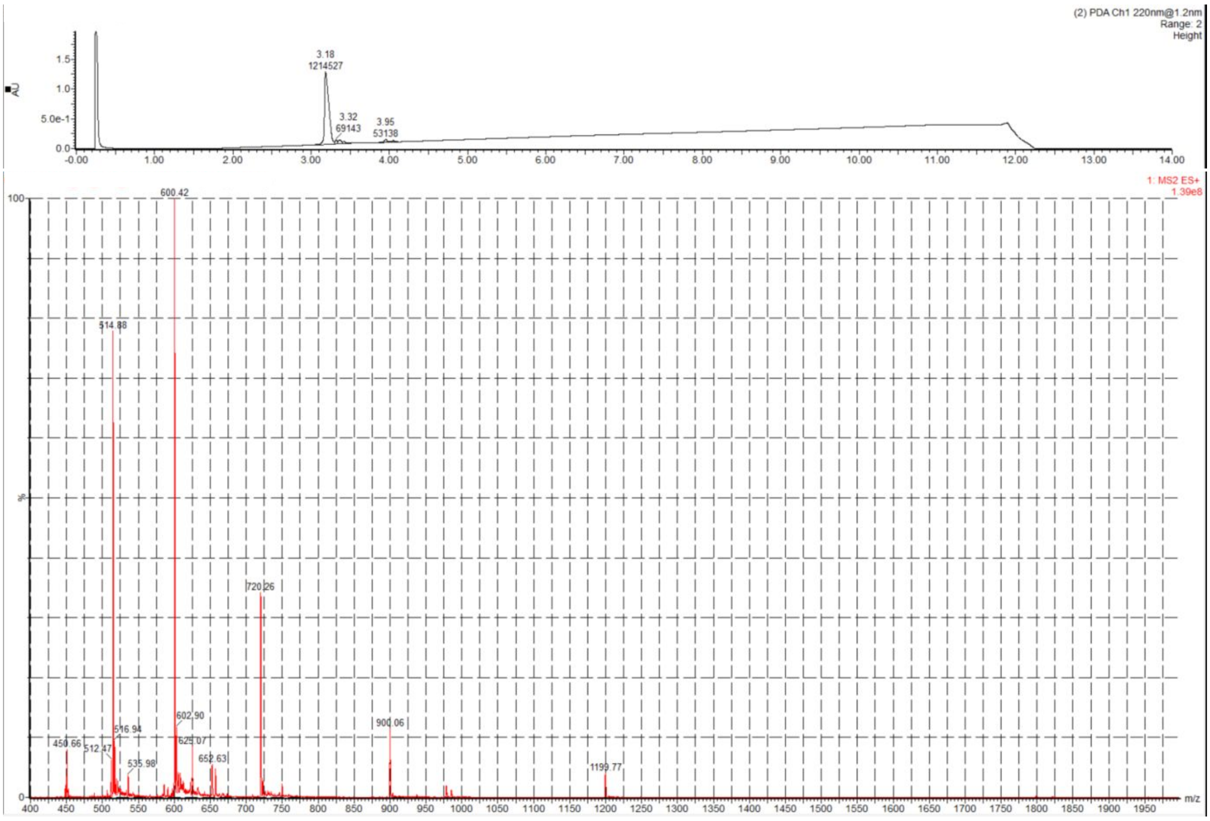
(2) PDA Ch1 220nm@1.2nm
Range: 2
Height
3.18
1214527
3.32
69143
3.95
53138
1: MS2 ES+
1.39e8
600.42
514.88
720.26
900.06
602.90
516.94
450.66
512.47
625.07
652.63
535.98
1199.77
m/z

SD3

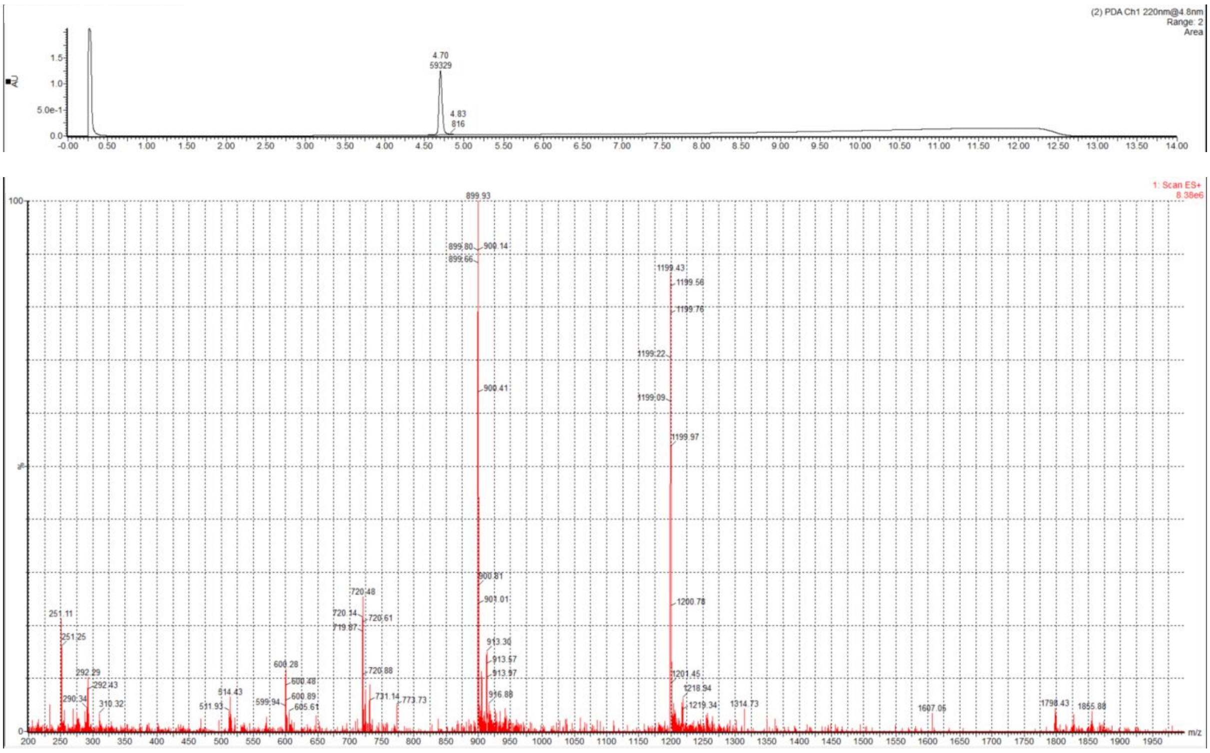
(2) PDA Ch1 220nm@4.8nm
Range: 2
Area
AU
4.70
59329
4.83
816
1: Scan ES+
8.38e6
899.93
1199.43
720.48
251.11
600.28
m/z

SD2

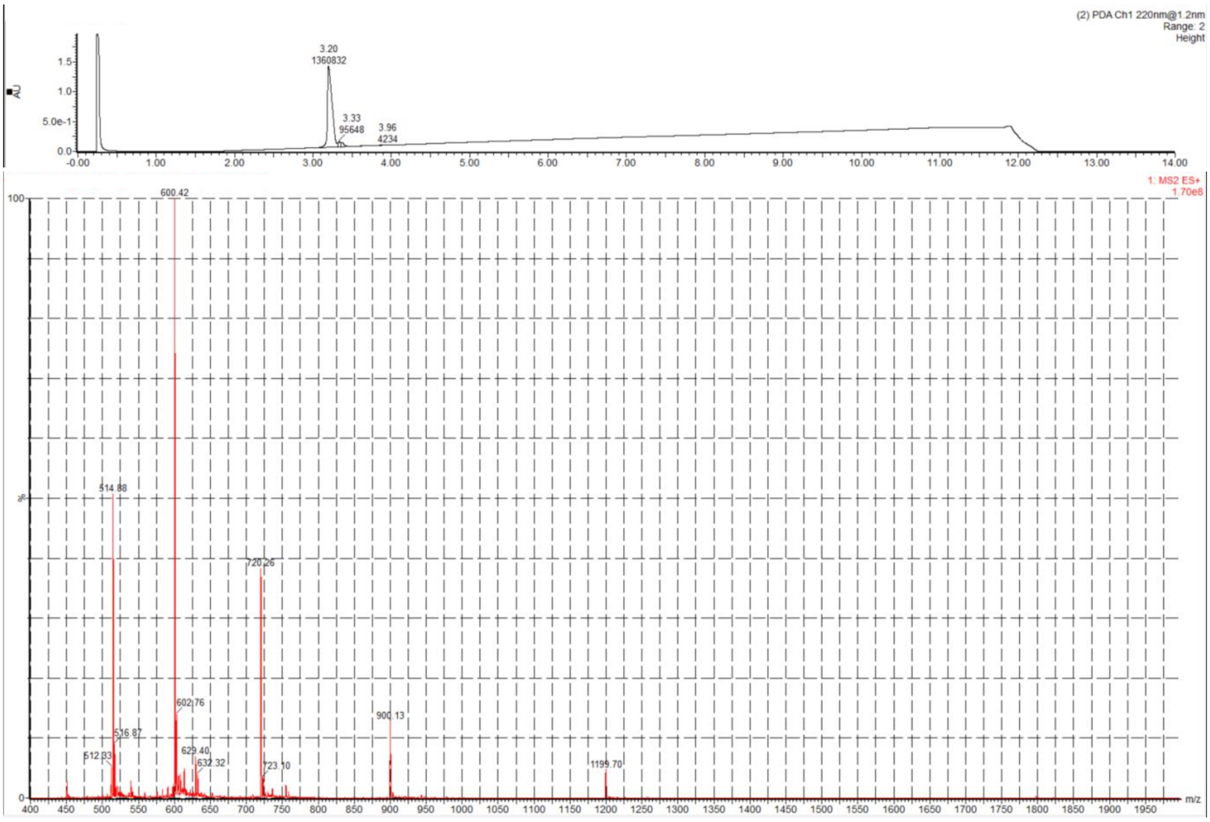
(2) PDA Ch1 220nm@1.2nm
Range: 2
Height
AU
3.20
1360832
3.33
95648
3.96
4234
1: MS2 ES+
1.70e8
600.42
514.88
720.26
602.76
516.87
512.33
629.40
632.32
723.10
900.13
1199.70
m/z

CD2

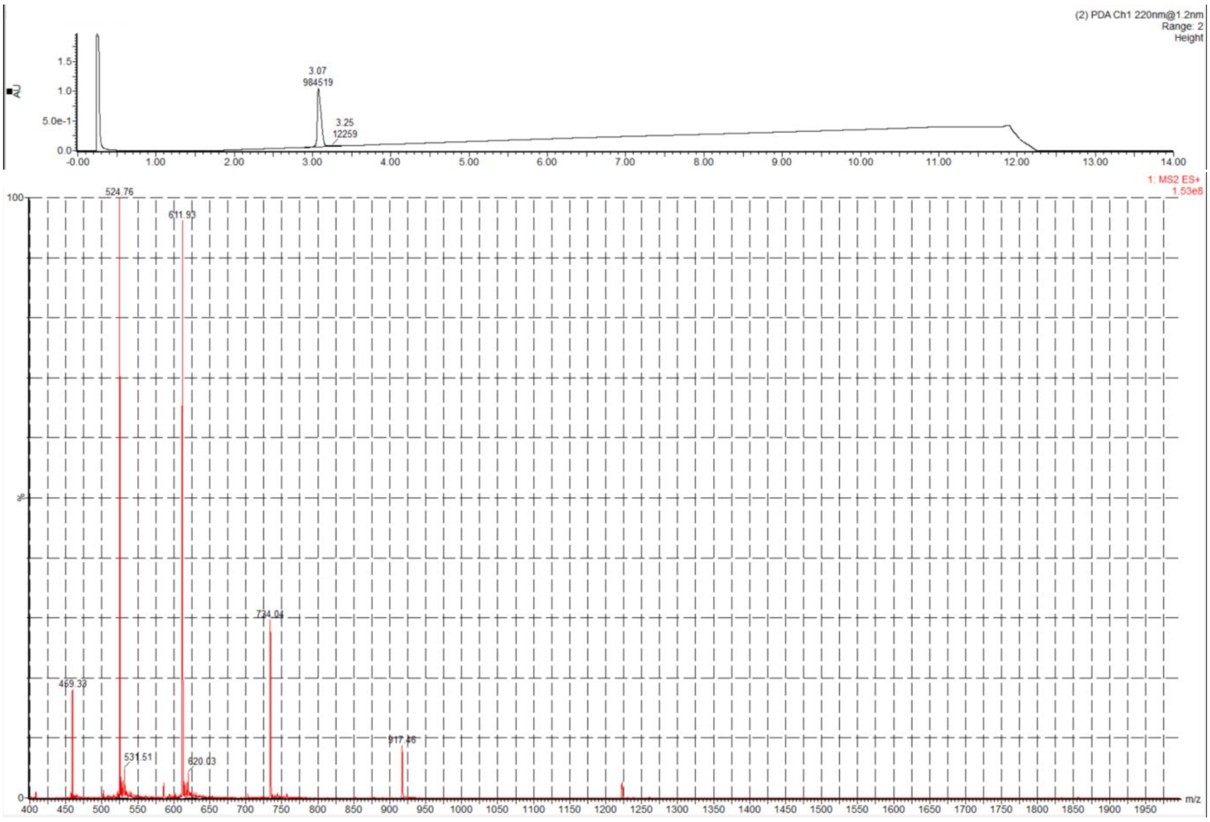
(2) PDA Ch1 220nm@1.2nm
Range: 2
Height
AU
3.07
984519
3.25
12259
1: MS2 ES+
1.53e8
524.76
611.93
724.04
459.33
531.51
620.03
917.46
m/z

CD4

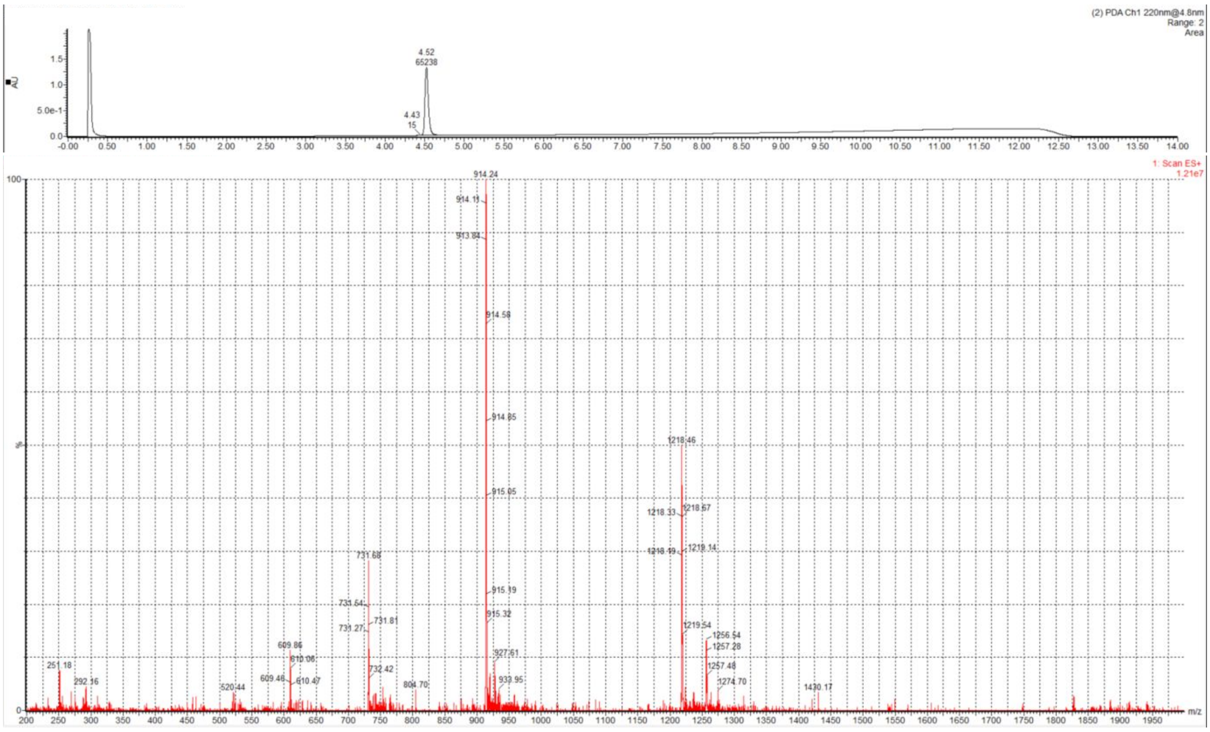
(2) PDA Ch1 220nm@4.8nm
Range: 2
Area
AU
4.52
65238
4.43
15
1: Scan ES+
1.21e7
914.24
914.11
913.84
914.58
914.85
915.05
915.19
915.32
927.61
933.95
1218.46
1218.33
1218.67
1218.19
1219.14
1219.54
1256.54
1257.28
1257.48
1274.70
1430.17
731.68
731.54
731.27
731.81
732.42
804.70
609.86
610.08
609.46
610.47
520.44
251.18
292.16
m/z

CD5

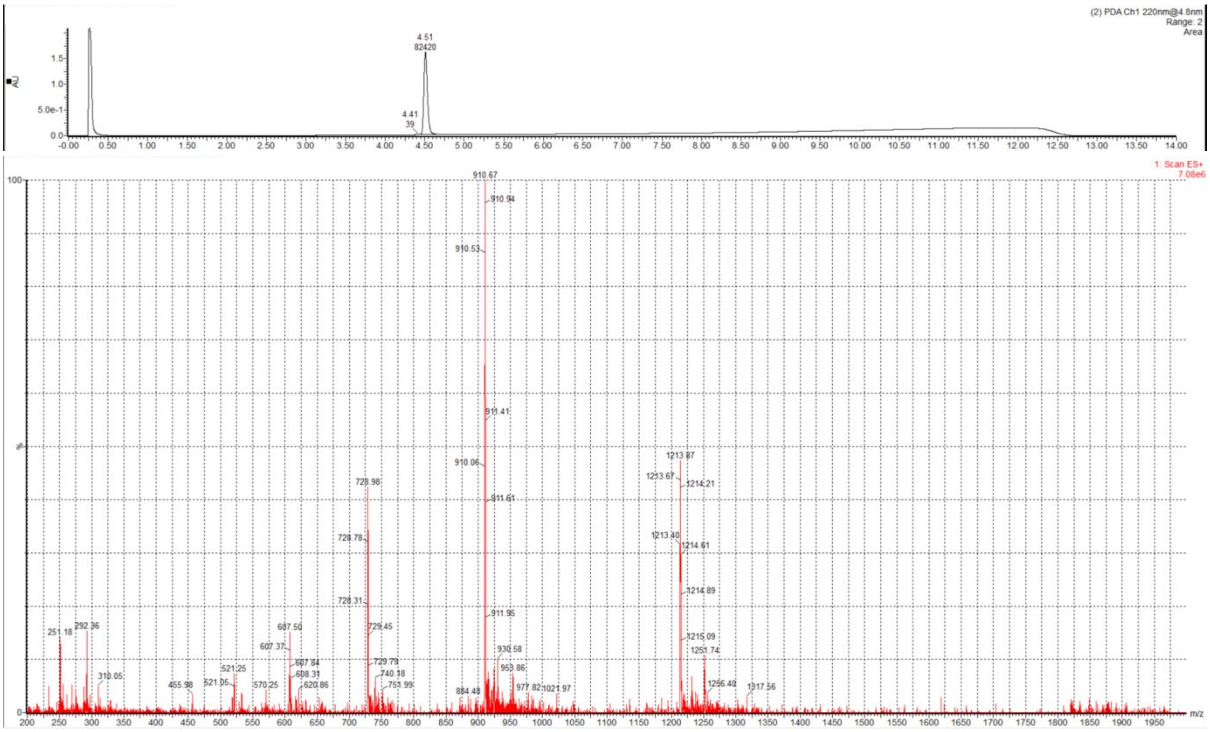
(2) PDA Ch1 220nm@4.8nm
Range: 2
Area
4.51
82420
4.41
39
1: Scan ES+
7.08e6
910.67
1213.87
728.98
m/z

CD11

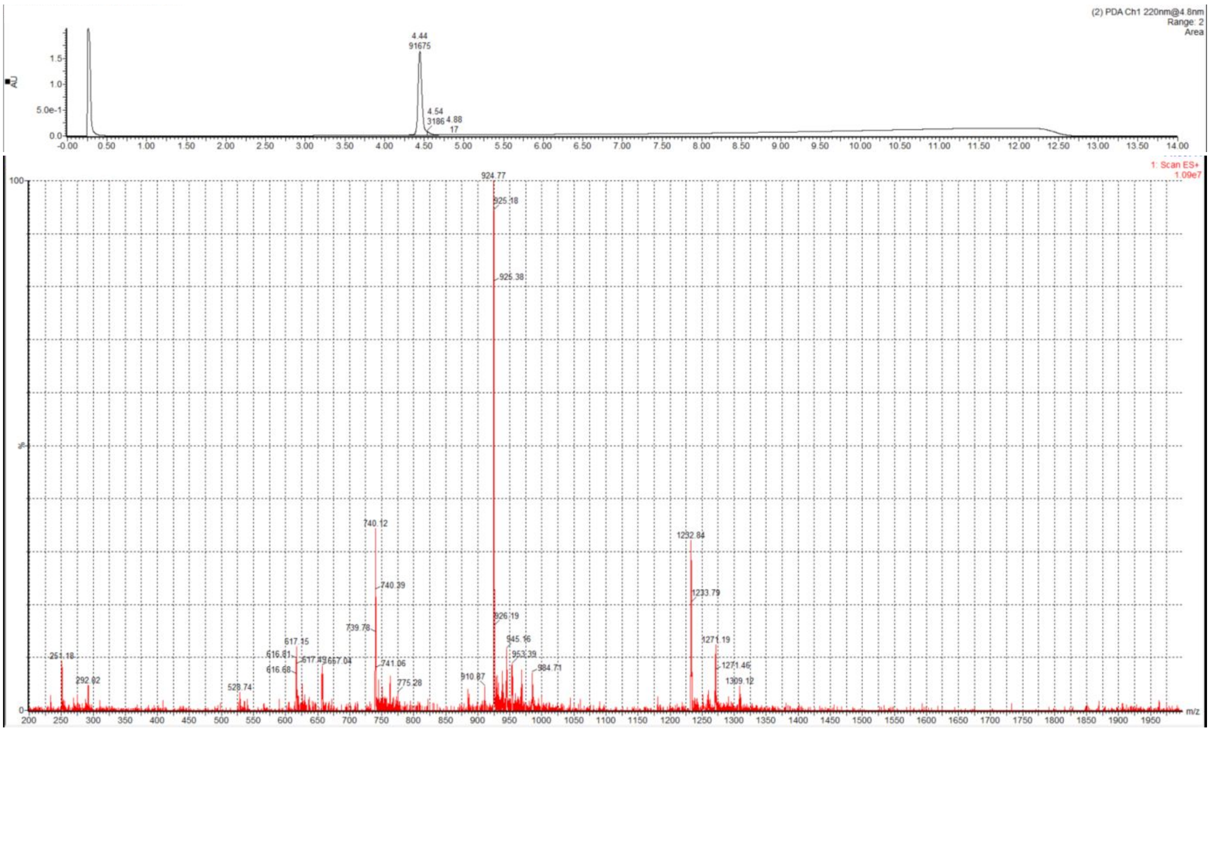
(2) PDA Ch1 220nm@4.8nm
Range: 2
Area
4.44
91675
1: Scan ES+
1.09e7
924.77
740.12
1232.84
m/z
